# NTnC-like genetically encoded calcium indicator with a positive and enhanced response and fast kinetics

**DOI:** 10.1038/s41598-018-33613-6

**Published:** 2018-10-15

**Authors:** Natalia V. Barykina, Danila A. Doronin, Oksana M. Subach, Vladimir P. Sotskov, Viktor V. Plusnin, Olga A. Ivleva, Anna M. Gruzdeva, Tatiana A. Kunitsyna, Olga I. Ivashkina, Alexander A. Lazutkin, Aleksey Y. Malyshev, Ivan V. Smirnov, Anna M. Varizhuk, Galina E. Pozmogova, Kiryl D. Piatkevich, Konstantin V. Anokhin, Grigori Enikolopov, Fedor V. Subach

**Affiliations:** 1 Moscow Institute of Physics and Technology, Dolgoprudny, 141701 Russia; 2 P.K. Anokhin Institute of Normal Physiology, Moscow, 125315 Russia; 3 National Research Center “Kurchatov Institute”, Moscow, 123182 Russia; 4 Lomonosov Moscow State University, Moscow, 119991 Russia; 5 Institute of Higher Nervous Activity and Neurophysiology of RAS, Moscow, 117485 Russia; 6 Pirogov Russian National Research Medical University, Moscow, 117997 Russia; 7 Federal Research and Clinical Center of Physical-Chemical Medicine of Federal Medical Biological Agency, Moscow, 119435 Russia; 8 Engelhardt Institute of Molecular Biology RAS, Moscow, 119991 Russia; 9 MIT Media Lab, Massachusetts Institute of Technology, Cambridge, MA USA; 10 Department of Anesthesiology, Stony Brook University Medical Center, Stony Brook, NY 11794 USA; 11 Center for Developmental Genetics, Stony Brook University, Stony Brook, NY 11794 USA

## Abstract

The NTnC genetically encoded calcium indicator has an advantageous design because of its smaller size, GFP-like N- and C-terminal ends and two-fold reduced number of calcium binding sites compared with widely used indicators from the GCaMP family. However, NTnC has an inverted and modest calcium response and a low temporal resolution. By replacing the mNeonGreen fluorescent part in NTnC with EYFP, we engineered an NTnC-like indicator, referred to as YTnC, that had a positive and substantially improved calcium response and faster kinetics. YTnC had a 3-fold higher calcium response and 13.6-fold lower brightness than NTnC *in vitro*. According to stopped-flow experiments performed *in vitro*, YTnC had 4-fold faster calcium-dissociation kinetics than NTnC. In HeLa cells, YTnC exhibited a 3.3-fold lower brightness and 4.9-fold increased response to calcium transients than NTnC. The spontaneous activity of neuronal cultures induced a 3.6-fold larger ΔF/F response of YTnC than previously shown for NTnC. On patched neurons, YTnC had a 2.6-fold lower ΔF/F than GCaMP6s. YTnC successfully visualized calcium transients in neurons in the cortex of anesthetized mice and the hippocampus of awake mice using single- and two-photon microscopy. Moreover, YTnC outperformed GCaMP6s in the mitochondria and endoplasmic reticulum of cultured HeLa and neuronal cells.

## Introduction

Genetically encoded calcium indicators (GECIs) based on green fluorescent protein-like (GFP-like) fluorescent proteins (FPs) are broadly utilized for the *in vivo* visualization of neuronal calcium activity^1^. As we previously described, FP-based calcium indicators may be classified into three major types by their molecular design (Supplementary Fig. 1a)^2^.

The first two types of GECIs, which include the fluorescence resonance energy transfer (FRET)-based and circularly permutated FP (cpFP)-based indicators, do not have the optimal design compared with the third type, the NTnC-like type of GECIs. The latter combines the advantages of the minimal troponin C (TnC) calcium (Ca^2+^)-binding domain from the toadfish *Opsanus tau*, utilized in the Twitch family of FRET indicators^3^, and the single fluorescent domain from the cpFP-based indicators. Thus, NTnC-like GECIs simultaneously possess the reduced number of Ca^2+^-binding sites (i.e., they bind two calcium ions per molecule instead of four) and the smaller molecular size of the indicator (i.e., they are composed of one FP instead of two FPs). The reduced number of Ca^2+^-binding sites in NTnC-like GECIs is thought to provide a more linear response to Ca^2+^ transients, thus facilitating the quantification of changes in the Ca^2+^ concentration^4^.

The NTnC-like design has additional advantages compared with other designs. For example, GCaMP6s and G-GECO1.2, representatives of the second type GECIs with green fluorescence, demonstrated reduced fluorescence recovered after photobleaching- (FRAP-) mobility in mammalian cells at both low and high Ca^2+^ concentrations^5^. The restricted mobility may be caused by undesired interactions of the calmodulin (CaM) and M13-peptide pair of these GECIs with intracellular environments. The CaM-based GCaMP6 indicator interferes with L-type calcium channels (Ca_v_1)/CaM-mediated excitation-transcription coupling in neurons and disrupts Ca^2+^ dynamics and gene expression^6^. Expression of GCaMP6 causes aberrant cortical activity in transgenic mouse lines^7^. The truncated version of TnC in NTnC-like GECIs has a smaller chance of having interacting partners in neurons^6,8^. Therefore, the NTnC-like type of GECIs is preferable among other types because it may be potentially inert to the neuronal environment and intermolecular interactions.

**Figure 1:**
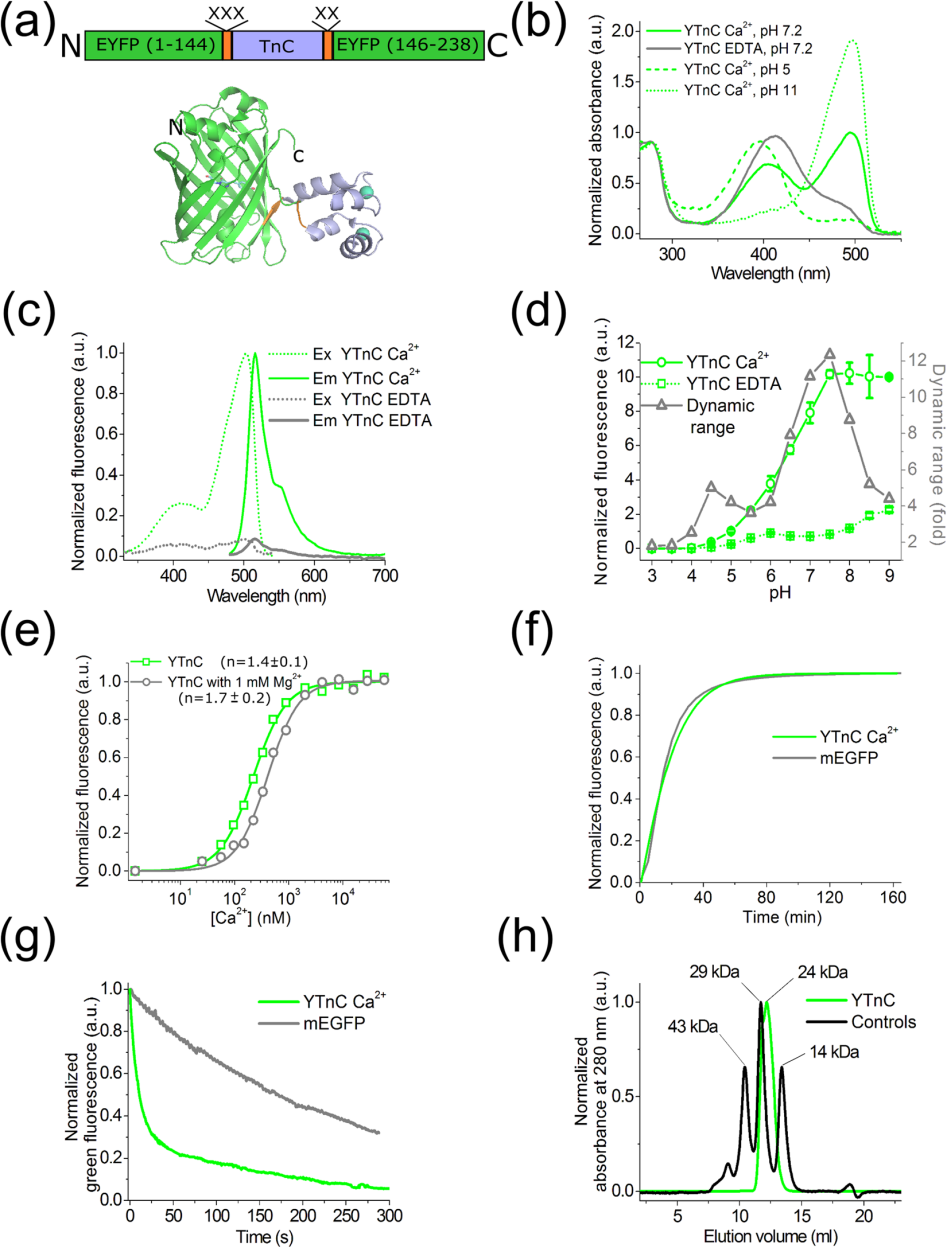
*In vitro* properties of the purified YTnC indicator. (**a**) A scheme of the original library for optimization of linkers in YTnC indicator and crystal structure of NTnC*2Ca^2+^ complex (pdb 5MWC) for the sensor having a similar design. (**b**) Absorbance spectra for YTnC in Ca^2+^-free and Ca^2+^-bound states at different pH values. (**c**) Excitation and emission spectra for YTnC in Ca^2+^-free and Ca^2+^-bound states. (**d**) Fluorescence intensity for YTnC in Ca^2+^-free and Ca^2+^-bound states as a function of pH. (**e**) Ca^2+^ titration curves for YTnC in the absence and presence of 1 mM MgCl_2_. (**f**) Maturation curves for YTnC in Ca^2+^-saturated state and mEGFP. (**g**) Photobleaching curves for YTnC in Ca^2+^-free state and mEGFP. The power of light before objective lens was 7.3 mW/cm^2^. (**h**) Fast protein liquid chromatography of YTnC (14 mg/ml) in 40 mM Tris-HCl (pH 7.5) and 200 mM NaCl buffer supplemented with 5 mM CaCl_2_. Error bars represent the standard deviation for the average of three records.

The recently published NTnC calcium indicator, a first representative of a novel NTnC-like design, consists of TnC from the swim bladder and white muscle of *O*. *tau* (tsTnC) as the Ca^2+^-binding moiety, inserted in the mNeonGreen FP^2^ (Fig. 1a). NTnC has a size of 311 amino acids, which is less than the size of cpFP- and FRET-based indicators by 105 and 245 a.a., respectively. In addition to the advantages of the NTnC-like design previously discussed, NTnC has a high fluorescence brightness and pH stability. Because of its inverted fluorescence response to calcium ions, NTnC makes non-active neurons or cells with low resting calcium concentrations visible, thus eliminating the need for the co-expression of a bright fluorescent cell marker. NTnC was successfully utilized for *in vivo* imaging of neuronal activity in awake mice^2^. Despite the listed benefits, NTnC has disadvantages, which are attributed to its low fluorescence change of 100% between its Ca^2+^-free and Ca^2+^-saturated states and slow Ca^2+^-dissociation kinetics.

As another green fluorescent GECI with the insertion of the Ca^2+^-binding domain, Camgaroo1, has a high 8-fold fluorescence contrast and utilizes enhanced yellow fluorescent protein (EYFP) as the fluorescent moiety^9^, we assumed that the replacement of the mNeonGreen fluorescent part in NTnC with EYFP may result in an indicator with an enhanced fluorescence response and faster kinetics. The previously reported NTnC indicator has an inverted phenotype, and GECIs with a positive fluorescence response to calcium are not currently available for the NTnC-like design, although GECIs with a positive phenotype have two major advantages over GECIs with an inverted phenotype. First, GECIs that have a positive Ca^2+^ response potentially enable three dimensional (3D) calcium imaging of the large-scale neuronal circuitry with high precision because neurons remain inactive most of the time^10^. Second, GECIs that have a positive phenotype should undergo less photobleaching during prolonged calcium imaging because their photobleaching rate in the Ca^2+^-unbound state is significantly reduced and they are not fully saturated with Ca^2+^ even during neuronal calcium activity (their ΔF/F response continues to increase at least up to 160 action potentials (APs)^3,11^).

Herein, we developed a single-fluorophore-based GECI that contains two Ca^2+^-binding sites, which is similar to the NTnC GECI but with the positive fluorescence response to Ca^2+^ and substantially improved fluorescence contrast and kinetics. This indicator, named YTnC, has TnC as the Ca^2+^-binding domain, which is inserted in the EYFP protein. We validated the main characteristics of the YTnC indicator *in vitro*. YTnC demonstrated an 11- and 3-fold higher fluorescence ΔF/F *in vitro* in the absence and presence of 1 mM Mg^2+^ ions, respectively, compared with the respective characteristics of NTnC *in vitro*. However, it had a 13.6- and 3.6-fold lower brightness and photostability, respectively, compared with NTnC *in vitro*. According to stopped-flow fluorimetry data, the Ca^2+^-dissociation kinetics for YTnC were 4-fold faster than for NTnC and 2-fold slower than for GCaMP6f. However, the kinetics of the Ca^2+^-association in the case of YTnC was up to 8-fold faster than that for GCaMP6f. During Ca^2+^ transients in HeLa mammalian cells, YTnC demonstrated a 4.9-fold higher ΔF/F than identified for the NTnC indicator. Using YTnC, we visualized Ca^2+^ dynamics during spontaneous activity in primary neuronal cultures with a 3.6-fold improvement in the ΔF/F values over NTnC. We also assessed the YTnC characteristics in neurons using patch clamp recordings and determined that YTnC robustly and linearly responded to the stimuli in the range of 2–100 APs; however, it had lower ΔF/F values than GCaMP6s. We successfully applied YTnC for the *in vivo* visualization of Ca^2+^ neuronal activity in the V1 cortical area of anesthetized mice and the hippocampus of freely moving mice using two-photon microscopy and an nVista miniscope, respectively.

## Results and Discussion

### Development of a novel green fluorescent calcium indicator with the NTnC-like design

To enhance the contrast and kinetics of NTnC GECI, we swapped its mNeonGreen fluorescent part with the EYFP protein and performed several rounds of further optimization using directed molecular evolution in a bacterial system^12^. We selected the EYFP protein as the fluorescent moiety for the new GECI because it was successfully used in the Camgaroo1 GECI and demonstrated high fluorescence contrast upon interaction with Ca^2+^ ions^9^. As a Ca^2+^-binding motif, we used the point mutated derivative of tsTnC excised from the NTnC GECI. We started with the generation of three libraries with the insertion of the TnC part between residues 144 and 146 (L1), 144 and 145 (L2), or 147 and 149 (L3) of EYFP (according to EGFP enumeration) and randomized both the 3- and 2-amino-acid-long linkers between the fluorescent EYFP and TnC components (Fig. 1a and Supplementary Fig. 2 and 3). The generated libraries were assessed using a two-step screening strategy. First, we performed imaging of the indicator’s libraries targeted to the *E*. *coli* periplasm on Petri dishes before and after treatment with a buffer that contained ethylenediaminetetraacetic acid (EDTA) to select clones with the highest fluorescence ratio. Second, the selected clones were analyzed in a bacterial suspension in a 96-well plate format. After the second step of screening, we confirmed that all three libraries have clones with a positive fluorescence response to elevation of the calcium concentration. In the L1 library, there were two clones with the maximal fluorescence contrast of 2.8-fold. This response was superior to the response observed for NTnC, thus demonstrating that our design strategy was valid.

Therefore, for further GECI development, we focused on the clones identified in the L1 library. We subjected two clones with the highest contrast to five sequential rounds of random mutagenesis followed by screening. During each round, we screened approximately 30,000 colonies to identify variants with the largest Ca^2+^-dependent changes in green fluorescence, according to the previously described protocol. After 5 rounds of random mutagenesis and selection, we chose a variant with the best performance in terms of contrast and brightness, named YTnC (EYFP derived TnC-based calcium indicator), which had 19 mutations relative to the original template (Supplementary Fig. 2). Among these mutations, 14 and 5 mutations were located in the fluorescent part and linkers, respectively. In the fluorescent domain, 6 mutations were internal and buried in the β-barrel, and 8 mutations were external. The six internal mutations L18M, F46L, K52E, F64L, V236A, and Y276T corresponded to the positions 18, 46, 52, 64, 163, and 203 in GFP, respectively (Supplementary Fig. 2 and 3). According to the crystal structure of EGFP, amino acids 64 and 203 are located close to the chromophore tripeptide^13^. The remaining internal mutations were identified outside of the immediate surroundings of the chromophore and are unlikely to significantly affect chromophore fluorescence.

### *In vitro* characterization of purified YTnC

First, we characterized the spectral and biophysical properties of the purified YTnC calcium indicator in the presence and in the absence of calcium ions (Table 1). At pH 7.2 in the Ca^2+^-saturated state, the absorbance of YTnC_sat_ exhibited two bands with peaks at 405 and 495 nm, respectively (Fig. 1b). When excited at the peaks, both bands of YTnC_sat_ fluoresced with a similar emission peak at 516 nm (Fig. 1c). In the Ca^2+^-free state, YTnC_apo_ exhibited one major absorption band with a maximum at 413 nm, and upon excitation, the emitted dim fluorescence peaked at 514 nm. In both the Ca^2+^-free and Ca^2+^-saturated states, the brightness of the 405–413-nm absorbing forms of YTnC was 9–17-fold lower than that of the fluorescent 495-nm form. In the Ca^2+^-saturated fluorescent state, the 495 nm band of the YTnC_sat_ indicator had a 6-fold lower brightness than EGFP (Table 1). The fluorescence ΔF/F dynamic range of the YTnC indicator was 1060 ± 37% (mean ± SD throughout the paper), which corresponded to a 10.6-fold improvement of the ΔF/F response over the NTnC GECI.

**Table 1:** *In vitro* properties of YTnC compared to NTnC.

Properties		Proteins			
		YTnC		GCaMP6fsat	NTnCapo
		apo	sat		
Absorbance maxima (nm)		413	495 (405)	497	505
Emission maxima (nm)		514	516 (516)	516	518
Quantum yield^a^		0.012	0.19 (0.03)	0.51	0.71 ± 0.05
ε (mM^−1^ cm^−1^)		28 ± 2^b^	29 ± 3 (20 ± 2)^c^	73.5^b^	108 ± 6^b^
Brightness vs EGFP (%)	Purified protein^d^	1	17 (2)	114	232
	HeLa cells^e^	ND	114 ± 23 (62 ± 3)	ND	188 ± 29 (202 ± 32)
ΔF/F (%)	0 mM Mg^2+^	1060 ± 37		2900 ± 97	100 ± 15
	1 mM Mg^2+^	290 ± 23		2600 ± 190	100 ± 35
p*K*a		5.2 ± 0.1, 8.2 ± 0.1	6.3 ± 0.1	6.34 ± 0.01	6.09 ± 0.07
K_d_ (nM)^f^	0 mM Mg^2+^	223 ± 10 [n = 1.4 ± 0.1]		375 ± 8 [n = 2.01 ± 0.08]	84 ± 6 [n = 1.9 ± 0.1]
	1 mM Mg^2+^	410 ± 19 [n = 1.7 ± 0.2]		492 ± 10 [n = 2.23 ± 0.09]	192 ± 40
K_d_^kin^ (nM)^f,g^		230 ± 200 [n = 1.4 ± 0.1]		450 ± 300 [2.4 ± 0.1]	94 ± 9 [n = 2.3 ± 0.1]
k_on_ (s^−1^ × M^−n^)^g^		1.9 ± 0.9 × 10^9^		3.5 × 10^15^	6 × 10^15^
k_obs_ (s^−1^)^h^		5.3 ± 0.3; 1.12 ± 0.06		0.63 ± 0.01	5.8 ± 0.1; 0.08 ± 0.001
k_off_ (s^−1^)^i^		0.96 ± 0.01		2.1 ± 0.1	0.8 ± 0.1; 0.05 ± 0.01^j^
t_1/2_^off^ (s)		0.78		0.35	3
Maturation half-time (min)^k^		ND	16	ND	23
Photobleaching half-time (s)^l^		ND	11 ± 4		40 ± 8

^a^QYs were determined at pH 7.20. EGFP (QY = 0.60^31^) and mTagBFP2 (QY = 0.64^32^) were used as reference standards for 495- and 405–413-nm absorbing states, respectively. ^b^Extinction coefficient was determined by alkaline denaturation. ^c^Extinction coefficient was estimated relative to YTnC_apo_ with the same absorbance at 280 nm. ^d^Brightness was calculated as a product of the quantum yield and extinction coefficient. ^e^Brightness was normalized to that of the control mCherry protein, which was equimolar expressed in HeLa cells using GFP-P2A-mCherry construct, where GFP was EGFP, YTnC_sat_ or NTnC_apo_ proteins, respectively (Supplementary Fig. 4). EGFP had a brightness of 100 ± 16% (100 ± 20%). Values in the brackets correspond to the conditions in the presence of DMEM medium supplemented with 20 mM HEPES, pH 7.40, 10% FBS, Glutamine, 50 U/ml penicillin, and 50 U/ml streptomycin. ^f^Hill coefficient is shown in square brackets. ^g^K_d_^kin^, Hill coefficients and k_on_ values were obtained by fitting the observed association rates (Fig. 2a–c) at 100–1300 nM Ca^2+^ concentrations (for YTnC and GCaMP6f) or 100–300 nM Ca^2+^ concentrations (for NTnC) to the equation k_obs_ = k_on_ × [Ca^2+^]^n^ + k_off_ (Fig. 2d). K_d_^kin^ = (k_off_/k_on_)^1/n^. ^h^k_obs_ values are shown for the Ca^2+^ concentration of 300 nM. First and second values correspond to fast and slow exponents, respectively. ^i^ Refined k_off_ values were determined from the dissociation kinetics records (Fig. 2f). ^j^In contrast to YTnC and GCaMP6f dissociation kinetics, NTnC kinetics do not agree with the two-state model. The NTnC dissociation curve was fitted to double exponential. k_off_ values were estimated from double exponential decay with individual exponent contributions of 0.48:0.52. ^k^EGFP had a maturation half-time of 14 min. ^l^EGFP had a photobleaching half-time of 170 ± 20 s.

To estimate different factors that may contribute to the value of the YTnC contrast, we investigated the spectral transitions of YTnC upon calcium binding. The binding of YTnC to Ca^2+^ ions was accompanied by a 4-fold increase in its absorbance at 495 nm and a 1.4-fold reduction in its absorbance at 413 nm (Fig. 1b). Thus, the absorbance response at 495 nm was approximately 3.5-fold lower than the fluorescence response of 1060% (Table 1). The latter difference is likely caused by the increase of the quantum yield of the 495 nm-absorbing form in the presence of Ca^2+^ ions. Therefore, the binding of YTnC to Ca^2+^ ions is simultaneously accompanied by a transition from one form of the chromophore (with absorbance at 413 nm) to another form (with absorbance at 495 nm) and by an increase of the quantum yield of the 495 nm-absorbing form. The 413 nm- and 495 nm-absorbing forms likely originate from different protonation states of the GFP chromophore. At pH 11, at alkaline conditions that favored the deprotonation of the GFP chromophore, YTnC_sat_ had one absorbance peak at 495 nm (Fig. 1b). At pH 5, in acidic conditions that facilitated the protonation of the GFP chromophore, the YTnC_sat_ protein had one absorbance peak at 405 nm. Consequently, the 495- and 405–413-nm absorbing forms with fluorescence maxima at 516 and 514 nm, respectively, may be attributed to the deprotonated (form A) and protonated (form B) forms of the GFP-like chromophore, similar to that observed for GFP^14^. Thus, the high fluorescence contrast of the YTnC indicator is mainly a result of the effective transition from the B form to the A form upon Ca^2+^ binding compared to the NTnC indicator for which the inverse A to B transition was incomplete^2^.

Given that the pH in neurons may vary up to 0.3 units^15,16^, it was important to characterize the pH stability of the YTnC indicator to ensure its proper utilization in neurons. YTnC exhibited a shift in p*K*_a_ values from two transitions with p*K*a 5.2 and 8.2 in the Ca^2+^-free state to 6.3 in the Ca^2+^-saturated state (Fig. 1d, Table 1). As a result, the fluorescence of both states, as well as the fluorescence dynamic range of YTnC, showed dependence on pH within the physiological range of pH 5–8. Similar to YTnC, the commonly used GCaMP6s indicator based on the pH-sensitive EGFP protein also had a pronounced pH-dependence of its fluorescence response within the pH range of 5–8^2^. In contrast, the NTnC indicator based on the pH-stable mNeonGreen FP had a lower sensitivity to pH changes (p*K*a 6.1 for NTnC_apo_ vs p*K*a 9.6 for GCaMP6s_apo_)^2,17^. Thus, sensitivity to pH was most likely inherited by YTnC and NTnC GECIs from their FP progenitors, and pH variations may contribute to the YTnC Ca^2+^ response to a greater extent than in the Ca^2+^ response of the NTnC indicator.

We further assessed the affinity of the YTnC indicator to Ca^2+^ ions. It is known that the free calcium concentration may vary in the range of 50–100 nM to 250–10000 nM in neuronal cytoplasm^18,19^. According to equilibrium binding titration experiments, YTnC demonstrated a K_d_ value of 223 ± 10 nM, which was 2.7-fold larger and 1.7-fold lower than the respective constants for NTnC and GCaMP6f (Fig. 1e and Table 1). The YTnC affinity to calcium ions was between 1.5-fold weaker to 2-fold stronger than the Ca^2+^ affinities of the Twitch-1/2/3 FRET indicators, which are based on the same TnC domain^3^. The equilibrium Hill coefficient for the YTnC indicator was lower than those for the NTnC and GCaMP6f GECIs, which provides evidence for the decreased cooperativity of Ca^2+^ binding by YTnC.

We subsequently characterized the response of YTnC to Ca^2+^ ions in the presence of 1 mM Mg^2+^, a concentration that resembles that in neuronal cytoplasm^20^. Following the addition of Mg^2+^ ions to buffers that contained different concentrations of Ca^2+^ ions, the K_d_ value for YTnC increases by 1.8-fold from 223 ± 10 nM at 0 mM Mg^2+^ to 410 ± 19 nM at 1 mM Mg^2+^ (Fig. 1e and Table 1); in turn, the K_d_ value for NTnC increased 2.3-fold under the same conditions. The Mg^2+^ addition practically did not affect the brightness of YTnC_sat_; however, it increased the brightness of YTnC_apo_ by 3.1-fold. Thus, at 1 mM Mg^2+^, the fluorescence ΔF/F value of YTnC was 3.7-fold lower than in the absence of Mg^2+^ ions; however, it was approximately 2.9-fold higher than that of the NTnC indicator.

We also characterized the maturation rate and photostability of YTnC using EGFP as a control. At 37 °C, the YTnC indicator in the Ca^2+^-saturated state had a similar maturation rate to EGFP (Fig. 1f), whereas NTnC_apo_ demonstrated a 1.4-fold slower rate than YTnC (Table 1). Under a wide-field blue light illumination (Ex: 470/40 BP, Em: 525/50BP, the power of light before 63 × 1.4 NA oil immersion objective lens: 7.3 mW/cm^2^), the YTnC photobleaching rate in the Ca^2+^-bound state was 15- and 3.6-fold faster than in the case of EGFP (Fig. 1g) and NTnC in the Ca^2+^-free state, respectively (Table 1).

In size-exclusion chromatography in the presence of Ca^2+^ ions, YTnC eluted as a monomer even at a high 14 mg/ml concentration (Fig. 1h) similar to NTnC and GCaMP6s^2^. Monomeric proteins are less cytotoxic during expression in mammalian cells^21^ and enable labeling of other proteins^22^.

Overall, the *in vitro* characterization indicated that YTnC had a 3-fold higher fluorescence ΔF/F response in the presence of 1 mM Mg^2+^ and a 1.4-fold faster maturation, but a 13.6-fold lower brightness, lower pH stability, 2.1-fold decreased affinity to Ca^2+^ ions in the presence of 1 mM Mg^2+^, and a 3.6-fold lower photostability than NTnC.

### Characterization of YTnC calcium indicator kinetics using stopped-flow fluorimetry

**Figure 2:**
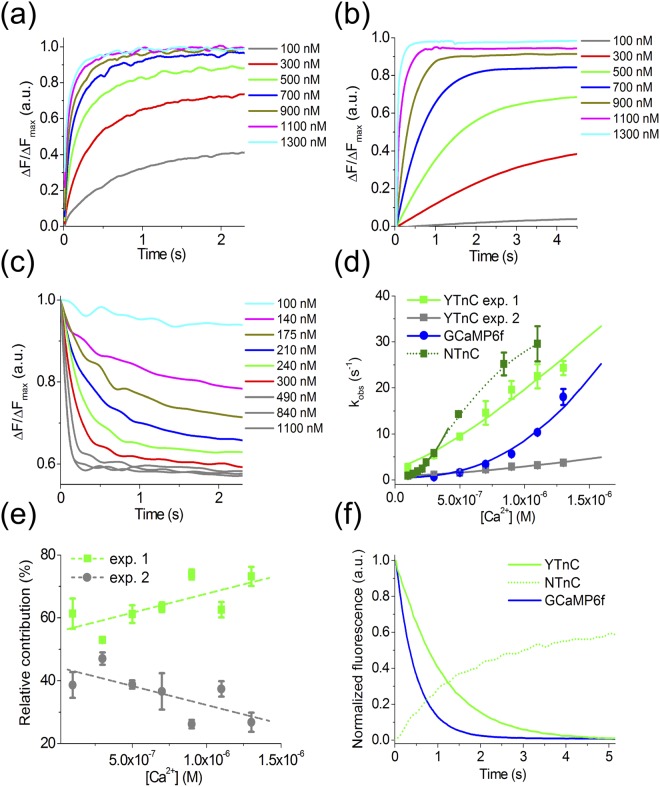
Calcium-association and -dissociation kinetics for the YTnC indicator investigated using stopped-flow fluorimetry. (**a**–**c**) Calcium-association kinetics curves for YTnC, GCaMP6f and NTnC, respectively. (**d**) Observed Ca^2+^-association rate constants determined from association curves for YTnC and control GCaMP6f and NTnC GECIs. For the YTnC indicator, fast (green) and slow (grey) exponents are shown. The solid lines represent the fitting curves to the equation k_obs_ = k_on_ × [Ca^2+^]^n^ + k_off._ (**e**) Relative contribution of monoexponents A_1_/(A_1_ + A_2_) and A_2_/(A_1_ + A_2_) for the YTnC indicator, where A_1_ and A_2_ are the pre-exponential factors in the association curve equation ΔFlu(t) = A_1_ × exp(−k^on^_obs1_ × t)−A_2_ × exp(−k^on^_obs2_ × t). The dots correspond to the A_1_ and A_2_ values, which were approximated by a linear equation (R^2^ = 5.083). (**f**) Calcium-dissociation kinetics for YTnC, GCaMP6f and NTnC GECIs. Starting concentration of free Ca^2+^ was 1000 nM. Three replicates were averaged for analysis. Whiskers correspond to SD error.

Neural activity causes rapid (hundreds of milliseconds) transients of intracellular calcium; thus, it is important to validate the temporal resolution of GECIs at physiological calcium concentrations. Therefore, we investigated the Ca^2+^-association and Ca^2+^-dissociation kinetics for the YTnC indicator *in vitro* using stopped-flow fluorimetry, with the NTnC and GCaMP6f GECIs used as references. The association curves for YTnC were well described by a bi-exponential function in the range of [Ca^2+^] = 100–1300 nM, which corresponds to the physiological calcium concentration in the neuronal cytoplasm (Fig. 2a). The association curves of the GCaMP6f and NTnC indicators with calcium ions were mono- and bi-exponential, respectively (Fig. 2b,c). Two exponents of the bi-exponential fit corresponded to the fast and slow observed Ca^2+^-association rate constants, denoted as k^on^_obs__1_ and k^on^_obs__2_, respectively (Fig. 2d,e; the NTnC slow component is not shown). The relative contribution of the fast exponent was predominant (53–73%) for all Ca^2+^ concentrations tested (Fig. 2e); therefore, all subsequent results correspond to the fast exponent. In the range of 300–1300 nM Ca^2+^ concentrations, which correspond to the minimal and medium elevated calcium concentrations in the cytoplasm of active neurons, YTnC demonstrated 8.4-fold (at 300 nM free Ca^2+^) to 1.3-fold (at 1300 nM free Ca^2+^) faster kinetics of Ca^2+^ binding than GCaMP6f GECI. In the similar free Ca^2+^ concentration range, YTnC had slightly slower calcium binding kinetics than NTnC (from 1.09–fold slower at 300 nM free Ca^2+^ to 1.3–fold slower at 1100 nM free Ca^2+^). Fitting the dependence of the observed Ca^2+^-association rate constants on the Ca^2+^ concentrations to the equation k_obs_ = k_on_ × [Ca^2+^]^n^ + k_off_ enabled us to estimate the Hill coefficient and the dissociation constant. Both the K_d_^kin^ and Hill coefficient values for the YTnC, NTnC and GCaMP6f indicators were rather similar to those determined from the equilibrium studies (Table 1). The half-time of YTnC-Ca^2+^-dissociation was 0.78 s, and it was 3.8-fold less than that for the NTnC-Ca^2+^ complex and 2.2-fold higher than for the complex of the commonly used indicator GCaMP6f with Ca^2+^ ions (Fig. 2f and Table 1). Thus, the YTnC indicator shows 1.3-fold slower association kinetics but 3.8-fold faster dissociation kinetics than the NTnC indicator and 8.4-fold faster association but 2.2-fold slower dissociation kinetics than the GCaMP6f indicator. The fast association-dissociation kinetics of the YTnC indicator with calcium ions is advantageous for monitoring calcium activity *in vivo* with better sensitivity and resolution of calcium transients during APs.

### Calcium-dependent response and brightness of the YTnC indicator in HeLa Kyoto mammalian cells

**Figure 3:**
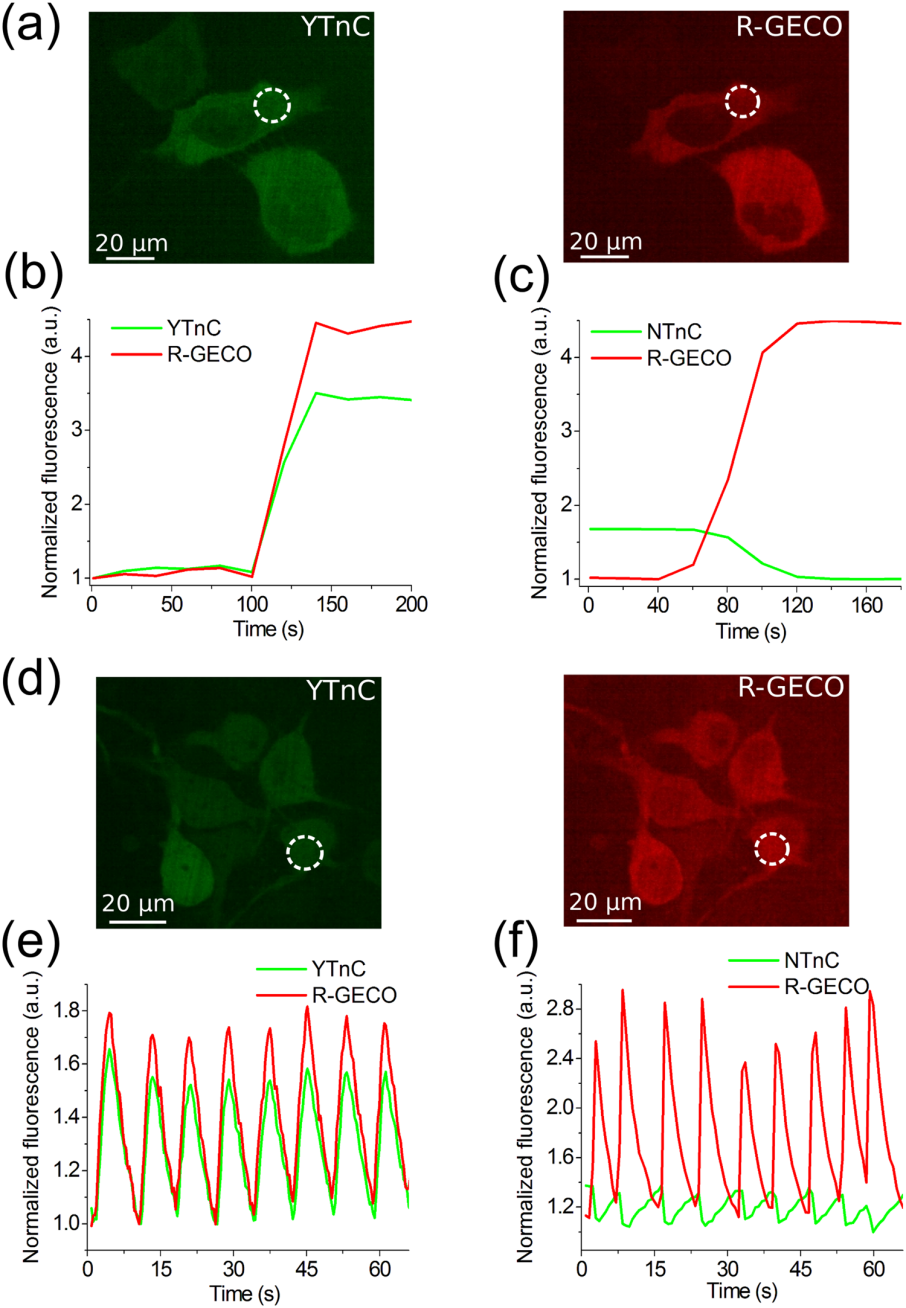
Response of the YTnC indicator to Ca^2+^ variations in HeLa Kyoto cells and cultured neurons. (**a**) Confocal images of HeLa Kyoto cells co-expressing green YTnC (panel a, left) and red R-GECO1 (panel a, right) calcium indicators. (**b**, **c**) The graphs illustrate changes in green fluorescence of YTnC (panel b) or control NTnC (panel c) indicators and in red fluorescence of the reference co-expressed R-GECO1 GECI in response to the addition of 2 mM CaCl_2_ and 2.5 μM ionomycin. The changes in panel b correspond to the area indicated with white circles in panel a. One example of three repeats is shown. (**d**) Dissociated neuronal culture co-expressing YTnC (panel d, left) and R-GECO1 (panel d, right) calcium indicators. (**e**,**f**) The graphs illustrate changes in red fluorescence of R-GECO1 (excitation 561 nm) and green fluorescence of YTnC (excitation 488 nm) (panel e) or control NTnC (panel f) as a result of spontaneous activity in neuronal culture. The graph in panel e illustrates changes in fluorescence in the area indicated with white circle in panel d. (**b**–**c**, **e**–**f**) The minimal fluorescence values were normalized to one.

To characterize the behavior of the YTnC indicator in mammalian cells, we investigated its response to the Ca^2+^ transients in HeLa Kyoto cells. After the addition of 2 mM CaCl_2_/2.5 µM ionomycin in Dulbecco’s phosphate-buffered saline (DPBS) with 20 mM 4-(2-hydroxyethyl)-1-piperazineethanesulfonic acid (HEPES), pH 7.40, the YTnC indicator demonstrated an average ΔF/F value of 198 ± 42% and a normalized ΔF/F value of 73 ± 33% relative to the red R-GECO1 in approximately 2 min. These values are 2.8 and 4.9-fold larger than the average and normalized ΔF/F values of 72 ± 5 and 15 ± 1% for NTnC, respectively, and 1.4-fold less than the response of R-GECO1 (Fig. 3a–c). When co-expressed in the same cells, the red calcium indicator R-GECO1 and YTnC demonstrated similar dynamics (Fig. 3b). Thus, in the cytoplasm of mammalian cells, the YTnC indicator demonstrated a 4.9-fold higher normalized fluorescence ΔF/F response to variations in the Ca^2+^ concentration than the NTnC GECI.

As the *in vitro* brightness of the YTnC indicator was 13.6-fold lower than that of NTnC, we compared its brightness with the NTnC and EGFP proteins in mammalian cells. We measured the green fluorescence intensities of GFPs in HeLa Kyoto cells transiently transfected with pCAG-NES-GFP-P2A-mCherry plasmids. The green fluorescence was normalized to the red fluorescence of mCherry that was co-expressed in the same cell in the equimolar amount to GFP ensured by the use of self-cleavable P2A peptide. For the EGFP and positive YTnC or inverted NTnC, we measured the fluorescence intensity of their brightest fluorescent states in DPBS with 20 mM HEPES, pH 7.40 in the presence of 2 mM CaCl_2_/2.5 µM ionomycin or 1 mM EDTA/2.5 µM ionomycin, respectively (Table 1 and Supplementary Fig. 4a). In these conditions in HeLa Kyoto cells, the YTnC indicator demonstrated a brightness that was similar or 1.65-fold dimmer than the EGFP and NTnC proteins, respectively. This relative improvement of the YTnC protein brightness in mammalian cells over bacterially expressed and purified protein might be associated with its better folding, maturation, and/or stability inside mammalian cells^23^.

To measure the brightness of YTnC under conditions that closely match *in vivo* conditions and estimate the potential impact from the presence of components of the cell culture medium, we determined the brightness and ΔF/F response of the YTnC indicator in HeLa Kyoto cells in Dulbecco’s modified eagle medium (DMEM) before and after the addition of 2.5 µM ionomycin (Table 1 and Supplementary Fig. 4b). In these conditions, the YTnC indicator co-expressed with mCherry via self-cleavable P2A peptide demonstrated brightness in the Ca^2+^-bound state that was 1.6- or 3.3-fold (p < 0.01, Mann-Whitney Rank Sum Test through the text) dimmer than the EGFP and NTnC proteins under identical expression and imaging conditions, respectively, and a ΔF/F response of 271 ± 33%. Taking into account that the brightness of the purified YTnC_sat_ protein was not affected by the presence of 1 mM Mg^2+^, the observed 2-fold decrease in YTnC_sat_ brightness in HeLa cells as a result of the DPBS to DMEM exchange may be explained by the influence of other factors but not the presence of Mg^2+^ ions. Thus, under the conditions most identical to *in vivo*, the YTnC indicator in fusion with P2A-mCherry has a 1.6- and 3.3-fold lower brightness than EGFP and NTnC proteins, respectively.

Overall, the results suggested that in terms of brightness, ΔF/F response, kinetics and calcium sensitivity, YTnC was appropriate for its further application in *in vivo* experiments.

### Visualization of spontaneous and induced neuronal activity in dissociated culture using YTnC indicator and confocal imaging

To validate the functionality of the YTnC indicator in neurons, we compared its response during spontaneous activity in dissociated neuronal cultures with those of the NTnC, GCaMP6s and R-GECO1 GECIs. Accordingly, we co-transduced neuronal cultures with recombinant AAV (rAAV) particles carrying YTnC, NTnC or GCaMP6s green indicators together with the reference R-GECO1 red calcium indicator under the control of the CAG promoter. The spontaneous activity of neurons in two- to three-week-old cultures was accompanied by an increase in the green fluorescence of the YTnC and GCaMP6s indicators and a decrease in the green fluorescence of the NTnC indicator with normalized ΔF/F values of 82 ± 12, 91 ± 115 and 23 ± 4%, respectively, relative to R-GECO1 (Fig. 3d–f, Supplementary Fig. 5 and Table 7). Thus, YTnC shows a similar and 3.6-fold larger normalized ΔF/F response during spontaneous neuronal activity than the GCaMP6s and NTnC indicators, respectively. The rise half-times for YTnC and R-GECO1 expressed in the same neurons were very similar, i.e., 1.0 ± 0.2 and 1.2 ± 0.2 s, respectively. The decay half-times for the YTnC and R-GECO1 indicators were approximately the same, i.e., 2.4 ± 0.3 and 2.4 ± 0.2 s, respectively. Thus, the kinetics of YTnC during neuronal spontaneous activity was approximately identical to that of R-GECO1. Overall, these data indicate that the YTnC indicator monitors the spontaneous activity of neurons with a substantially 3.6-fold higher or similar ΔF/F response compared with NTnC or GCaMP6s and R-GECO1 indicators, respectively.

Because purified YTnC protein showed poor photostability *in vitro*, we estimated its resistance to photobleaching during confocal microscope imaging. The imaging of neurons that expressed YTnC during 33 min under a 60x oil objective lens using 80% power of a 488 nm laser (17.3 µW/cm^2^ before objective lens) resulted in a 24 ± 5% loss of green fluorescence (Supplementary Fig. 6). Thus, the YTnC indicator has photostability that is acceptable for short-term confocal imaging but may hinder long-term imaging.

To further estimate the ΔF/F response of YTnC compared with GCaMP6s and R-GECO1 GECIs, we stimulated dissociated neuronal cultures transfected with CAG-NES-R-GECO1-P2A-NES-YTnC or CAG-NES-R-GECO1-P2A-NES-GCaMP6s constructs with an external electric field (Supplementary Fig. 7 and Supplementary Table 1). The normalized (relative to R-GECO1) ΔF/F responses of NES-YTnC in fusion with NES-R-GECO1-P2A were 1.9-fold higher than the same characteristics for NES-GCaMP6s in the same fusion. We determined that the brightness of the GCaMP6s indicator in neurons transfected with CAG-NES-R-GECO1-P2A-NES-GCaMP6s construct was very dim relative to the bright red fluorescence of the R-GECO1 indicator. We assumed that in neurons, the NES-GCaMP6s maturation/expression from the CAG-NES-R-GECO1-P2A-NES-GCaMP6s construct was inefficient. However, HeLa cells transfected with the same plasmid appeared bright green and bright red following the addition of ionomycin (data not shown). To address this issue, we co-transduced neuronal cultures with the mixture of rAAV particles carrying GCaMP6s and NES-R-GECO1 indicators. In this case, the normalized (relative to NES-R-GECO1) ΔF/F responses of GCaMP6s were 6.3-fold higher than the same characteristics for NES-YTnC in fusion with NES-R-GECO1-P2A (Supplementary Table 1). We concluded that the ΔF/F response amplitude of the YTnC indicator in fusion with R-GECO1-P2A during the stimulation of neuronal cultures with an external electric field was 1.9-fold larger or 6.3- and 5.4-fold lower than the response of GCaMP6s in the same fusion or the responses of non-fused GCaMP6s and R-GECO1 indicators, respectively.

### Characterization of the YTnC sensor in dissociated neuronal culture with whole-cell patch clamp

**Figure 4:**
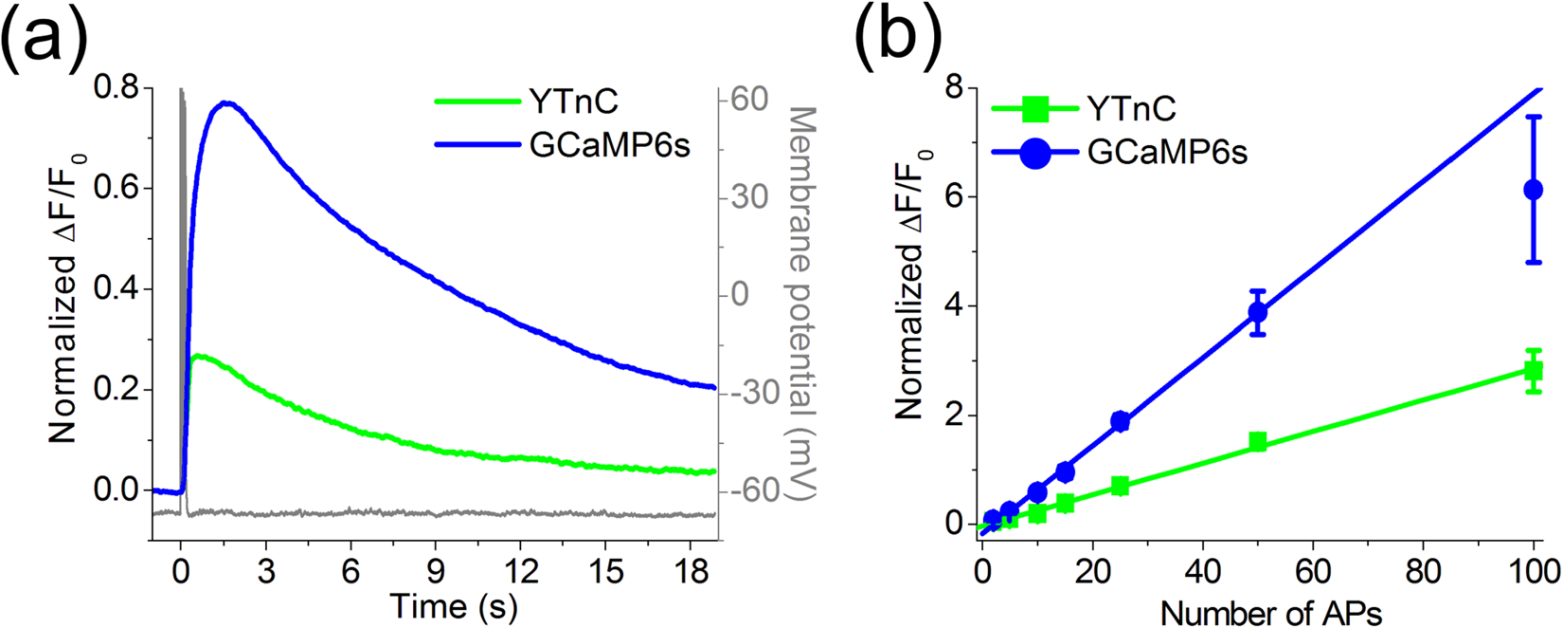
Fluorescence changes in neurons expressing YTnC and GCaMP6s indicators in dissociated culture in response to intracellularly induced APs. (**a**) Fluorescence changes in GECI-expressing cells to the train of 10 APs intracellularly induced with a frequency of 50 Hz. Ca^2+^ responses were averaged across representative recorded neurons from different wells (N = 6 and 7 neurons from 3 and 4 cultures for GCaMP6s and YTnC, respectively). Example of intracellular recording (grey) was obtained from one representative cell. (**b**) Dependence of the amplitudes of responses induced by different numbers of APs in neurons expressing YTnC and GCaMP6s. The linear regression shown in the figure was calculated for the 2–50 AP subset for both YTnC (R^2^ = 0.9959) and GCaMP6s (R^2^ = 0.9981). Note that in the range of 2 to 50 APs, dependence is linear for both indicators, whereas the amplitude of response to 100 APs in GCaMP6s-expressing neurons lies well below the linear regression line. At the same time, the response of YTnC to 100APs is located directly on the 2–50 regression line, i.e., dependence remains linear even for responses to strong stimulation. Values are shown as the means ± SEM.

To qualitatively characterize the performance of the YTnC sensor in neurons, we compared the fluorescence responses of cultured neurons that expressed YTnC, GCaMP6s, and R-GECO1 GECIs to intracellular stimulation using whole-cell patch recording. Neurons of the primary dissociated culture were co-transduced with YTnC or GCaMP6s green indicators together with reference R-GECO1 red GECI. We initially measured the green and red fluorescence changes in neurons in response to the train of 10 APs. The non-normalized responses of neurons that expressed YTnC and R-GECO1 to 10 APs are shown in Supplementary Fig. 8. For a more accurate comparison, the responses of R-GECO1 to 10 APs were subsequently used for the normalization of the YTnC and GCaMP6s responses in the same cell. Both indicators demonstrated fast and reliable changes in the fluorescence levels in response to the 10 AP train (Fig. 4a). The neurons that expressed YTnC showed faster kinetics of the Ca^2+^ responses than the GCaMP6s-positive cells. Thus, the half-rise and half-decay times for YTnC were 1.6- and 1.9-fold (p < 0.01) less than those for GCaMP6s (192 ± 6 ms vs 304 ± 11 ms, respectively, and 5.2 ± 0.7 s vs 9.7 ± 1.2 s, respectively; Supplementary Table 2). At the same time, the ΔF/F and signal-to-noise ratio (SNR) were 2.6- and 2.9-fold (p < 0.01) greater for the Ca^2+^ responses measured in GCaMP6s-expressing neurons than YTnC-expressing cells.

We also estimated the linearity of responses elicited by a different number of APs in the neurons that expressed YTnC and GCaMP6s GECIs using intracellular stimulation. We recorded the responses to a 50 Hz burst of 2–100 APs induced through the patch pipette. In the range of 2–50 APs, both indicators showed a linear dependence of the maximal ΔF/F values from the number of APs (Fig. 4b). However, the response of GCaMP6s to 100 APs showed notable saturation, whereas the responses of the YTnC indicator remained linear on the whole range tested (2–100 APs) (Fig. 4b). This difference may be related to the higher affinity of the GCaMP6s indicator to Ca^2+^ ions compared with that of YTnC.

Thus, in neurons, YTnC robustly and linearly responded to intracellular stimulations, with a 2.6-fold lower response amplitude and faster kinetics of association-dissociation with Ca^2+^ ions than the GCaMP6s indicator.

### *In vivo* two-photon imaging of neuronal activity in the visual cortex of awake mice using the YTnC indicator

**Figure 5:**
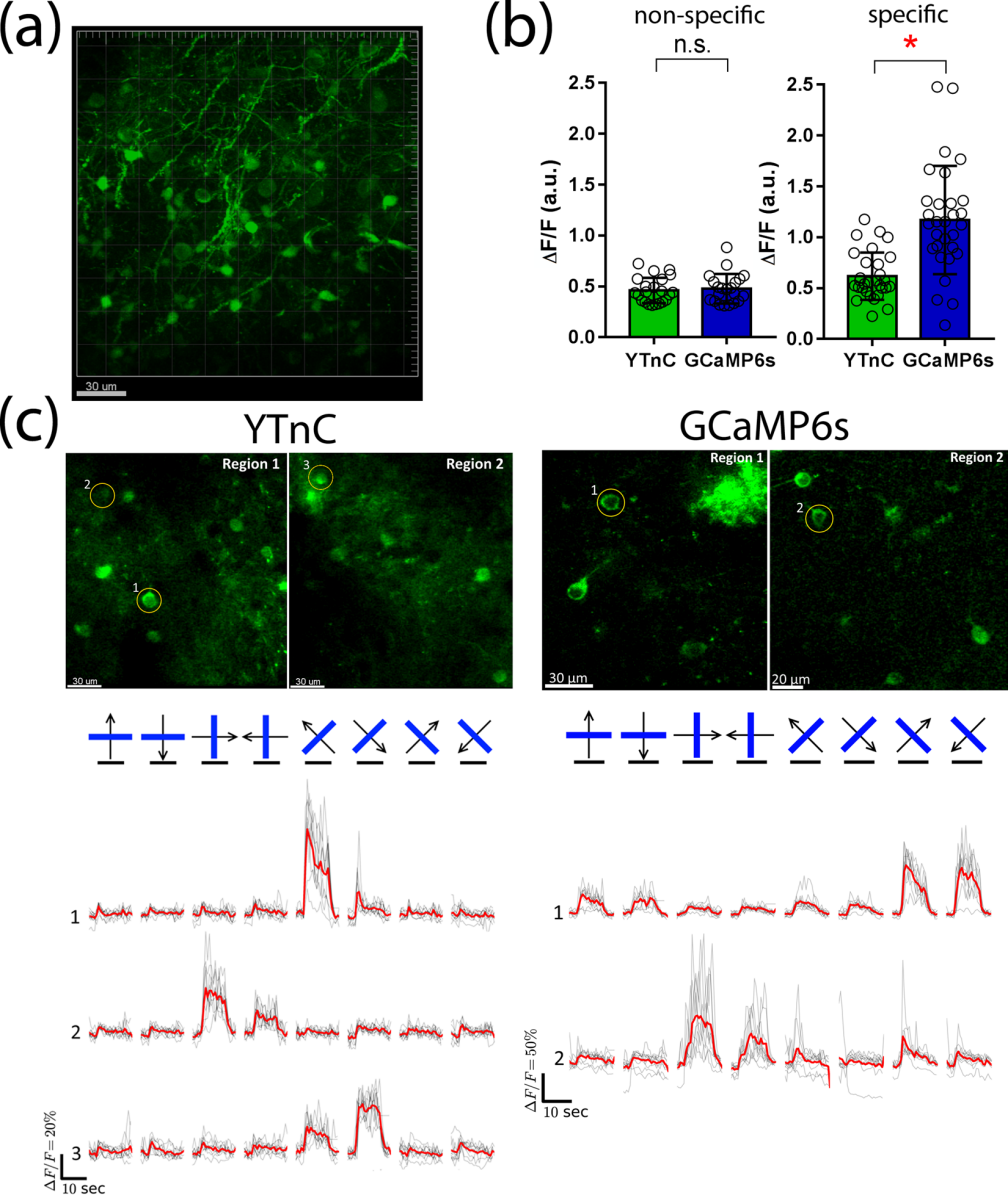
*In vivo* drifting grating-evoked neuronal activity in the mouse cortex visualized with YTnC and GCaMP6s calcium indicators using two-photon microscopy. (**a**) Example of the 3D reconstruction of YTnC-positive cells in the V1 visual cortex area using 960 nm excitation light. Block size, 317 × 317 × 350 μm. (**b**) Averaged ΔF/F responses corresponding to spontaneous (non-specific) and grating-evoked (specific) activity across neurons (n = 9, YTnC; n = 5, GCaMP6s and n = 3, YTnC; n = 2, GCaMP6s, respectively) in the V1 area for the YTnC and GCaMP6s indicators, respectively. (**c**) Two-photon images of two regions from V1 layer 2/3 neurons captured during presentation of drifting grating to the mouse for YTnC and GCaMP6s indicators. Averaged ΔF/F responses during presentation of drifting gratings (eight directions, 10 repetitions) are shown for the selected neurons. The directions of the drifting gratings (blue lines) are shown with arrows (in black). Grey and red lines correspond to the individual and averaged traces across ten repetitions, respectively. Black horizontal lines correspond to the time of grating presentation.

To compare the performance of the YTnC and GCaMP6s indicators in the classical two-photon *in vivo* application, we further performed two-photon calcium imaging of the YTnC and GCaMP6s indicators in the 2/3 layer (L2/3) of the V1 visual cortex of anesthetized head-fixed mice during the presentation of drifting grating as a visual stimulus. The YTnC and control GCaMP6s indicators were delivered to L2/3 of the primary visual cortex (V1) of the mice using rAAV particles. In the 6–7 weeks post infection, we identified cytoplasmic expression with additional nuclear expression of YTnC in neuronal bodies (15–25 μm in diameter) according to the green fluorescence (Fig. 5a). In the case of GCaMP6s-expressing neurons, the contrast between the cytoplasm and nucleus was greater. Using two-photon excitation with 960 nm light, the YTnC- and GCaMP6s-expressing neurons were visible at a depth up to 400 μm. The maximum density of the fluorescent neurons was identified in the outer part of L2/3 (60–150 μm from the brain surface). We subsequently imaged the calcium neuronal activity during the presentation of a PC monitor with black-white gratings moving to 8 different directions, as a visual stimulus for the YTnC and GCaMP6s-expressing mice. We recorded the activity from 15 neurons from 2 mice and 23 neurons from 2 mice for the YTnC and GCaMP6s expressing mice, respectively. The imaging was performed 3 months after the viral transduction.

We initially analyzed the spontaneous neuronal activity in the soma of neurons that was not specific to the grating presentation. The fractions of these non-specifically active neurons were similar: approximately 33 and 30% for the YTnC and GCaMP6s indicators, respectively. The averaged ΔF/F values for the YTnC and GCaMP6s spikes were approximately the same: 0.46 ± 0.12 and 0.48 ± 0.15 (n.s., p = 0.4379), respectively (Fig. 5b). The non-specific ΔF/F response of YTnC relative to GCaMP6s in the mouse cortex was similar to the ΔF/F response of YTnC during the spontaneous activity of the dissociated neuronal culture. Thus, YTnC exhibits spontaneous activity in the visual cortex of mice with the same ΔF/F sensitivity.

We subsequently compared the responses of the YTnC and GCaMP6s indicators that were specific to the direction of the grating (Fig. 5c and Supplementary Video 1). The fractions of these specifically activatable neurons related to all imaged neurons were approximately 20 and 9% for YTnC and GCaMP6s, respectively. The fractions of the grating-evoked neurons related to the neurons from the same fields of view (FOVs) were approximately 19 ± 4 and 10 ± 4% (each estimated from the two FOVs) for YTnC and GCaMP6s, respectively. According to published data, the fractions of the responsive NTnC- and GCaMP6s-expressing cells^2,24^ were 1.4-fold lower or 3.5-fold higher than for YTnC. The fraction of specifically evoked neurons expressing GCaMP6s in this study was also 6.3-fold lower than the published data^24^. This discrepancy may be explained by the significant variation of the fractions of specifically responding cells depending on the imaging region in the visual cortex (in our study for YTnC and GCaMP6s, it varied from 0 to 22% and 0 to 13%, respectively) and the different types of anesthesia (in our study, it was urethane). YTnC showed a 1.9-fold lower (p < 0.0001) averaged ΔF/F response of 0.62 ± 0.23 compared with the ΔF/F value of 1.17 ± 0.53 for the GCaMP6s indicator. The decreased response of YTnC during stimulus-evoked neuronal activity correlates with its 2.6-fold lower ΔF/F values than GCaMP6s on patched cultured neurons (Fig. 4). Thus, the YTnC indicator exhibits stimulus-evoked neuronal activity with a 1.9-fold lower ΔF/F response than GCaMP6s.

Because the YTnC indicator has a smaller size than GCaMP6s, the YTnC indicator may have better sensitivity in spines than GCaMP6s. From another perspective, the poor one-photon photostability of the purified YTnC protein *in vitro* may prevent its two-photon imaging in spines, which prompted us to compare the sensitivity and photostability of the YTnC and GCaMP6s indicators in neuronal spines. During spontaneous neuronal activity in the L2/3 of the visual cortex, the averaged ΔF/F responses of the YTnC and GCaMP6s indicators in spines were similar, i.e., 0.80 ± 0.30 and 0.78 ± 0.30 (n.s., p = 0.1014), respectively (Supplementary Fig. 9). Compared with the ΔF/F responses of the YTnC indicator relative to GCaMP6s during spontaneous activity in the soma, there was no improvement in the YTnC response over GCaMP6s in the neuronal spines. The fluorescence of the YTnC indicator was photobleached by 24% after a 200 sec imaging session of the spines, which was 16-fold higher than the 1.5% photobleaching of the GCaMP6s indicator (Supplementary Fig. 9d). Thus, using two-photon microscopy *in vivo*, YTnC visualizes spontaneous calcium activity in spines with a similar response as GCaMP6s but with a lower photostability.

Overall, these data show that the YTnC indicator is appropriate for at least 3-month long *in vivo* two-photon calcium imaging in the mouse brain and enables the detections of spontaneous and stimulus-evoked calcium transients in neurons with similar and 1.9-fold lower ΔF/F responses than the GCaMP6s indicator, respectively.

### *In vivo* recording of neuronal activity in the hippocampus of freely moving mice using YTnC and an nVista miniaturized microscope

**Figure 6:**
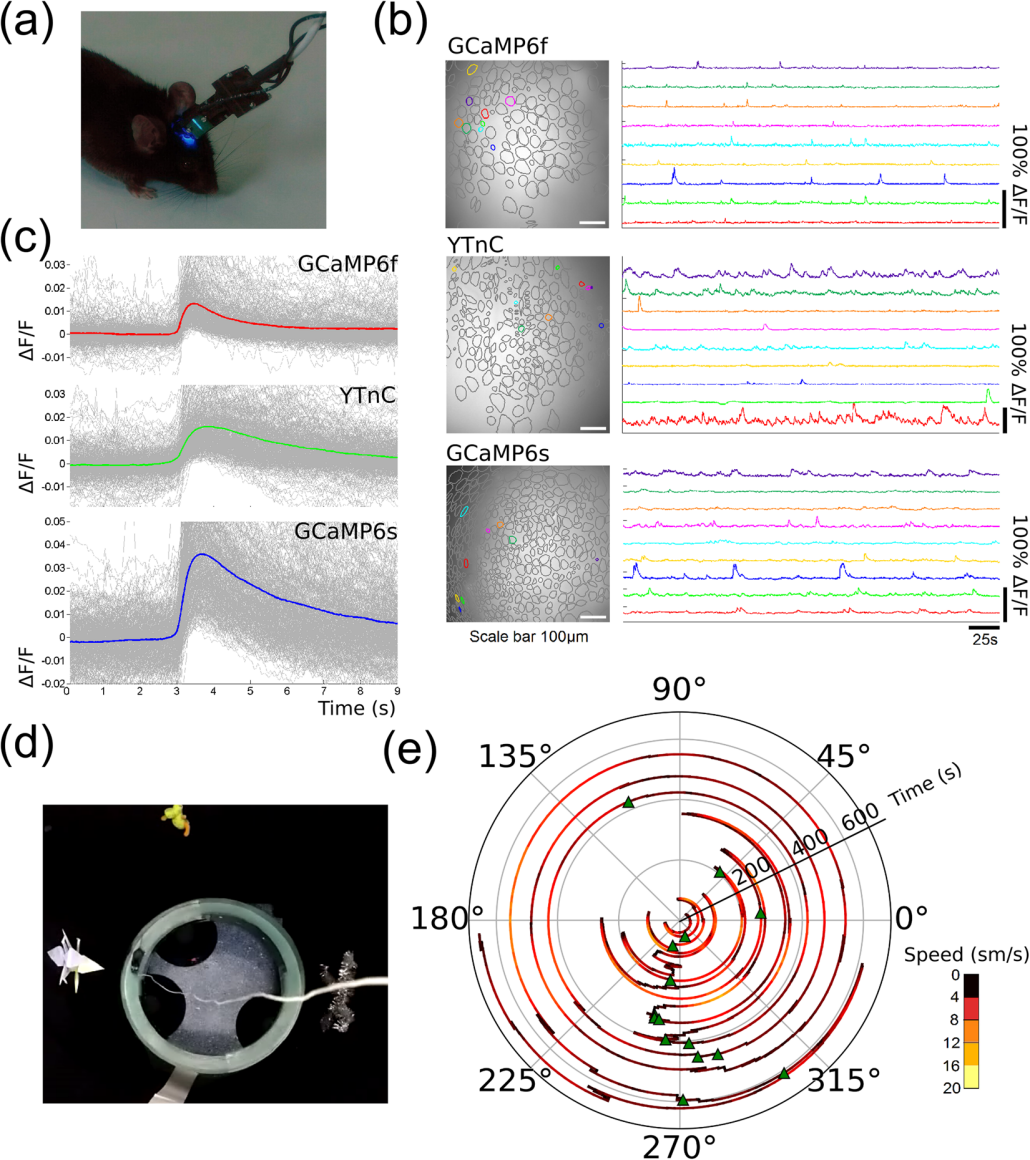
*In vivo* neuronal Ca^2+^ activity in hippocampus of freely behaving mice visualized with calcium indicators YTnC, GCaMP6f, GCaMP6s and an nVista HD system. (**a**) Photo of an nVista HD miniature microscope mounted to the head of mice. (**b**) Spatial filters and sample traces obtained from a 10-min imaging session with freely behaving mice expressing GCaMP6f, YTnC and GCaMP6s. (**c**) Mean spikes for calcium indicators GCaMP6f, YTnC and GCaMP6s; spikes exceeding the 4 MAD threshold were aligned at the moment of the very start of the peak (3 s). (**d**) Photo of O-shaped track with landmarks. (**e**) Circular plot of mouse trajectory during the exploration of circular track, synchronized with the spikes of a place cell (green triangles). The sensors GCaMP6f, YTnC and GCaMP6s were delivered to the hippocampus with rAAV (AAV-CAG-NES-GCaMP6f, AAV-CAG-NES-YTnC and AAV-CAG-GCaMP6s, respectively) particles.

To examine whether the YTnC indicator may be used for *in vivo* recording of neuronal activity with an nVista head-mounted miniaturized microscope, we visualized neuronal calcium activity in the CA1 field of the hippocampus of freely moving mice during the exploration of a novel context (custom made circular track, Fig. 6). This behavioral paradigm is intended to be used for the evaluation of the dynamic properties of establishing place cells. We installed the nVista microscope over the microendoscope lens implanted in the hippocampus of mice previously transduced with rAAV particles carrying YTnC or control GCaMP6f and GCaMP6s green calcium indicators under the control of the CAG promoter (Fig. 6a). In the YTnC-expressing mouse under anesthesia, we registered the neuronal activity in the CA1 and dentate gyrus (DG) areas of the hippocampus (Supplementary Video 2). To confirm the expression of the YTnC and GCaMP6f indicators in the CA1 and DG areas of the hippocampus, we performed a *post mortem* analysis of the brain tissue using immunohistochemistry (Supplementary Fig. 10).

In awake mice, we successfully imaged and assigned 562, 306, and 1617 cells (or 187, 153, and 404 cells per one mouse) to YTnC, GCaMP6f, and GCaMP6s, respectively (Supplementary Table 3). Identified cells and examples of their temporal dynamics are presented in Fig. 6b. Based on the spike detection routine (Supplementary Fig. 11), we determined that 30, 49, and 58% of the assigned cells were active for YTnC, GCaMP6f, and GCaMP6s-expressing neurons, respectively. On average, YTnC-expressing neurons demonstrated 1.3-fold and 2.6-fold fewer spikes per second than GCaMP6f and GCaMP6s-expressing neurons, respectively. This difference in the detected neuronal activity is likely related to the lower brightness and smaller ΔF/F values for the YTnC indicator compared with the GCaMP6s and GCaMP6f indicators. We suggest that the faster dissociation kinetics for GCaMP6f and higher affinity to calcium ions in the case of the GCaMP6s indicator also contribute to this difference. As expected, the mean spike of YTnC had rise and decay half-times of 0.7 and 2.4 s, respectively, which were similar to those for GCaMP6s and larger than those for GCaMP6f (Fig. 6c and Supplementary Table 3). The averaged peak ΔF/F value of 0.016 ± 0.01 and the SNR value of 9.2 for YTnC were similar to the values for GCaMP6f. The averaged peak ΔF/F and SNR values for YTnC were 1.4-fold (p < 0.01) lower and 1.5-fold larger than the respective values for GCaMP6s.

The photostability of YTnC during a single 10-minute imaging session was slightly lower than that for GCaMP6s (Supplementary Fig. 12a and b). The YTnC indicator also showed slight photochromic behavior because its green fluorescence recovered between imaging sessions (Supplementary Fig. 12c). However, the YTnC lower photostability and photochromic behavior did not hinder the fast calcium imaging using the NVista miniscope for at least fourteen 10-min imaging sessions during two days.

We subsequently correlated the neuronal calcium activity exhibited by the YTnC indicator in one field of view of the hippocampus with the mouse movement in the O-shaped track (Fig. 6d and Supplementary Video 3). As a result, we identified the neuron that was specifically activated in the certain portion of the track between 225 and 315° (Fig. 6e). This space specific activity of place neuronal cells has previously been registered in the CA1 area of the mouse hippocampus with the nVista miniscope and the GCaMP3 GECI^25^. Thus, we demonstrated that YTnC could be successfully used for the visualization of hippocampal neuronal dynamics during the exploration of a novel context and for the identification of place cells in freely moving mice using the nVista HD miniscope.

As the YTnC indicator remains suboptimal compared to the widely utilized GCaMP6s indicator, we further attempted to identify the potential advantages or applications for the YTnC indicator for which it would be better than GCaMP6s.

### YTnC targeting to the different cellular compartments of HeLa cells

The Ca^2+^-binding domain of the YTnC indicator is inserted in the middle of the protein molecule so its N- and C-termini are the same as in EGFP, in contrast to GCaMP6s that has an M13-peptide and Ca^2+^-binding domain on its N- and C-terminal ends, respectively. We assumed that this difference in the indicator’s design may be beneficial for YTnC in terms of its tolerance to the fusions with different proteins for targeting it to the different cellular compartments. To examine this hypothesis, we created different N- and C-terminal fusions of YTnC and compared their localization, brightness and dynamic range with the same fusions of the GCaMP6s indicator in HeLa cells.

**Figure 7:**
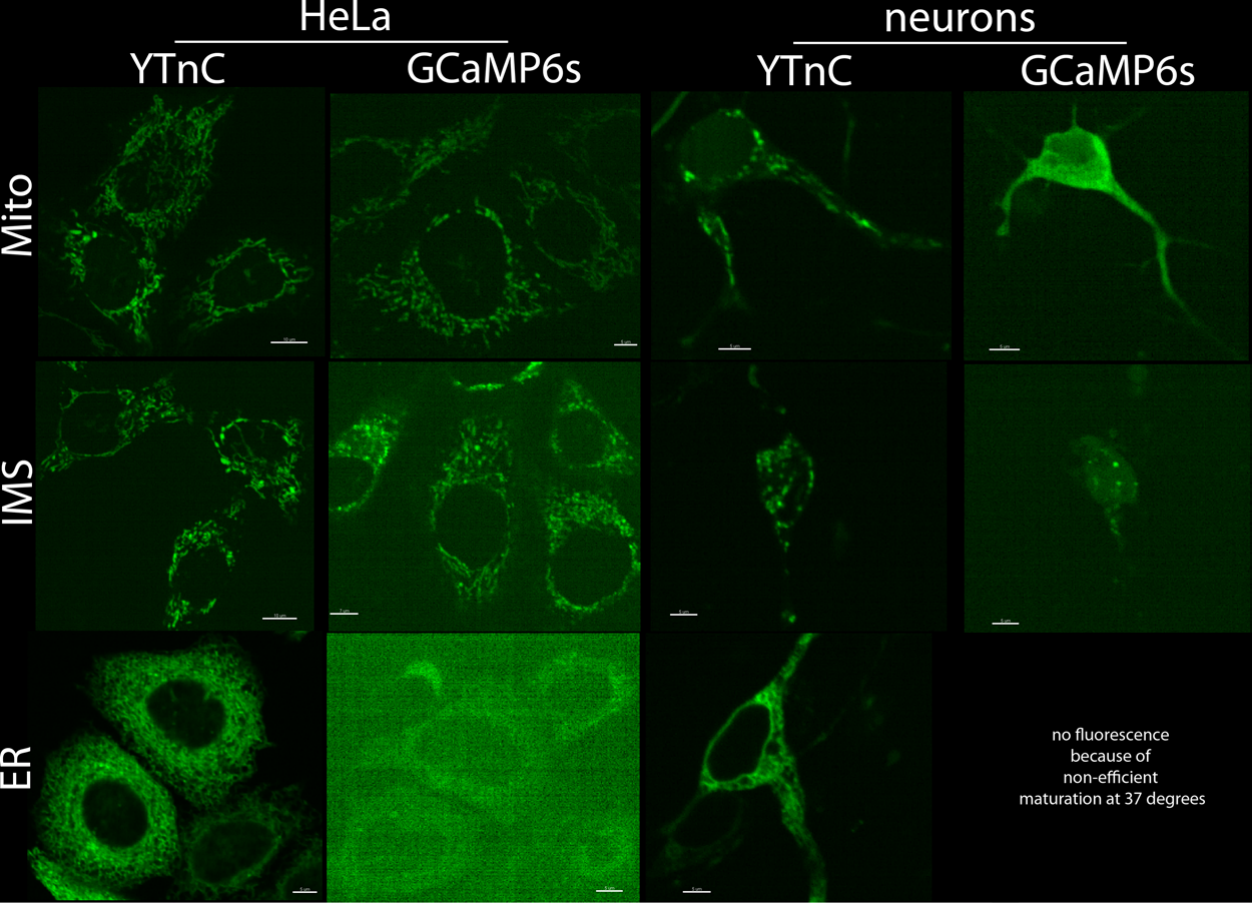
Localization of YTnC and GCaMP6s calcium indicators targeted to the different compartments of HeLa and neuronal cells. Confocal images of cells transiently expressing the listed fusions. The DNAs coding YTnC and GCaMP6s sensors were delivered to the HeLa and neuronal cells using transient transfection with lipofectamine or calcium-phosphate precipitation, respectively.

According to the confocal imaging of the HeLa cells that transiently expressed different fusions, YTnC demonstrated good localization for N-terminal fusions that nicely targeted it to the mitochondrial lumen (Mito), mitochondrial intermembrane space (IMS), endoplasmic reticulum (ER) (Fig. 7, Supplementary Fig. 13 and Supplementary Table 4), nuclei (H2B) and plasma membrane (Lyn) (Supplementary Fig. 14). N-terminal fusions of GCaMP6s also targeted it to the mitochondrial lumen (Mito) and mitochondrial intermembrane space (IMS) but less efficiently, and compared with the same YTnC fusions, more cells demonstrated cytoplasmic mislocalization in the case of GCaMP6s (Supplementary Fig. 13). GCaMP6s with an N-terminal ER-targeting signal practically did not mature in HeLa cells at 37 °C (Fig. 7 and Supplementary Fig. 13). However, its incubation at room temperature resulted in the appearance of green fluorescence in ER-like structures (Supplementary Fig. 15). Compared with GCaMP6s_sat_ in the same fusions, following the addition of 2.5 μM ionomycin, the brightness of YTnC_sat_ normalized to the brightness of NES-mCherry protein was 4.8-, 22- and 4.2-fold (p < 0.01) higher for the Mito, ER and Lyn fusions, respectively, and 2.7-fold (p = 0.0113) higher for the IMS fusion (Supplementary Table 5). One exception was the N-terminal fusion with H2B, in which YTnC was 3.1-fold (p < 0.01) dimmer than H2B-GCaMP6s. The brightness of NES-YTnC_sat_ in the cytoplasm was 4.7-fold (p < 0.01) dimmer than the brightness of GCaMP6s_sat_. The brightness of YTnC_sat_ N-terminally fused to the Mito, IMS, and ER signals was 3.1-, 3.4-, and 2.6-fold (p < 0.01) higher than the brightness of NES-YTnC_sat_, respectively. The YTnC indicator in fusion with H2B and Lyn signals had a brightness similar to NES-YTnC_sat_. The brightness of GCaMP6s_sat_ N-terminally fused to the Mito, IMS, ER, and Lyn signals was 7.1-, 3.8-, 40-, and 9.3-fold (p < 0.01) lower than the brightness of non-fused GCaMP6s_sat_, respectively. GCaMP6s_sat_ in fusion with H2B had a similar brightness to non-fused GCaMP6s_sat_. Thus, YTnC in N-terminal fusions shows proper localization and preserves the high brightness in contrast to GCaMP6s, which demonstrated a higher percent of cytoplasmic mislocalizations and lost its brightness by 3.8–40-fold in most N-terminal fusions examined.

We also compared the ΔF/F responses of the YTnC and GCaMP6s indicators in N-terminal fusions in HeLa cells to the calcium increase induced by a 2.5 μM ionomycin addition. YTnC outperformed GCaMP6s in this parameter by 3.4- and 2.5-fold (p < 0.01) for the Mito and IMS fusions, respectively (Supplementary Table 5). The YTnC ΔF/F response in the ER was significantly larger than that for GCaMP6s. We could not quantitatively compare the ΔF/F responses of YTnC and GCaMP6s in the ER because we were not able to detect a response in the case of the GCaMP6s indicator. Nuclei- and membrane-localized GCaMP6s demonstrated 3.5-fold (p < 0.01) larger and similar ΔF/F responses than YTnC, respectively. In N-terminal fusions, YTnC demonstrated similar or 1.3-fold larger ΔF/F responses than NES-YTnC, with the exception of fusion with the ER for which the ΔF/F significantly decreased by 9-fold (p < 0.01). In the latter case, the low response of YTnC is likely connected with the high calcium affinity of YTnC, which is improper for high calcium concentrations in the ER. The GCaMP6s indicator in N-terminal fusions with the Mito, IMS, ER and Lyn showed 14.5-, 14.5-, significantly and 3.6-fold (p < 0.01) lower ΔF/F responses than non-fused GCaMP6s, with the exception of the fusion with H2B in which GCaMP6s demonstrated the same ΔF/F response. Thus, most N-terminal fusions do not greatly affect the ΔF/F response of the YTnC indicator and substantially reduce the ΔF/F response of GCaMP6s to ionomycin-induced calcium transients in HeLa cells by 3.6–14.5-fold.

We also compared the ΔF/F responses of the Mito and IMS N-terminal fusions of the YTnC and GCaMP6s indicators in HeLa cells to the calcium transients induced by 10 μM thapsigargin, which induces Ca^2+^ ion release from intracellular Ca^2+^ stores, such as the ER and its subcompartments, via inhibition of Ca^2+^-ATPases in the sarco-endoplasmic reticulum^26^. In this case, Mito-YTnC and IMS-YTnC showed 2.2-fold (p < 0.01) larger or similar normalized (relative to R-GECO1) ΔF/F responses compared with the respective fusions for GCaMP6s (Supplementary Table 6 and Supplementary Fig. 16). Compared with GCaMP6s_sat_, following the addition of thapsigargin, the brightness of YTnC_sat_ normalized to the brightness of the NES-R-GECO1_sat_ indicator was 4.2- and 1.7-fold (p < 0.01) higher for the Mito and IMS fusions, respectively (Supplementary Table 6). Thus, in terms of the localization, brightness and ΔF/F response to ionomycin or thapsigargin addition, YTnC outperforms GCaMP6s in the lumen and IMS of mitochondria and in the ER of HeLa cells.

C-terminal fusions of YTnC and GCaMP6s with β-actin and α-tubulin were aimed to target indicators to cytoskeletal microtubules (Supplementary Table 4). According to confocal imaging of HeLa cells that transiently expressed YTnC and GCaMP6s in C-terminal fusions, the most cells expressing YTnC with β-actin showed improper cytoplasmic mislocalization with some exceptions (Supplementary Fig. 17). In the case of GCaMP6s-β-actin fusion, we also identified a sufficient number of cells with improper cytoplasmic mislocalization; however, the percent of cells with proper localization was higher than with YTnC-β-actin fusion. All imaged cells that expressed the fusions of YTnC and GCaMP6s with α-tubulin had improper cytoplasmic mislocalization, and in both cases, we could not identify a single cell with microtubule-like structures (Supplementary Fig. 17). In fusions with β-actin and α-tubulin, the brightness of YTnC_sat_ was 4.3- and 17.2-fold (p < 0.01) reduced, respectively. In fusions with β-actin and α-tubulin, the brightness of GCaMP6s_sat_ was also 3.3- and 10.3-fold (p < 0.01) reduced, respectively. C-terminal fusions with β-actin and α-tubulin also reduced the ΔF/F response of YTnC by 1.8- and 2.9-fold (p < 0.01), respectively. The GCaMP6s indicator in fusion with β-actin and α-tubulin demonstrated the same and 2.4-fold (p < 0.01) lower ΔF/F response than the non-fused GCaMP6s. Thus, in C-terminal fusions with β-actin, GCaMP6s outperforms YTnC in terms of the proper localization, brightness and ΔF/F response; however, in fusion with α-tubulin, both indicators demonstrated improper localization, and GCaMP6s-α-tubulin showed a higher brightness and ΔF/F response than with YTnC-α-tubulin.

Overall, compared with GCaMP6s in the most N-terminal fusions tested, YTnC demonstrates better localization and preserves a higher brightness and ΔF/F response in HeLa cells.

### YTnC targeting to the different cellular compartments of neuronal cells

As the YTnC indicator demonstrated better performance in N-terminal fusions in HeLa cells compared with the same fusions for GCaMP6s, we decided to compare the properties of the YTnC and GCaMP6s indicators in the same fusions and two additional neuron-specific N-terminal fusions during their transient expression in cultured neurons. Furthermore, we aimed to compare the behavior of the YTnC and GCaMP6s indicators in C-terminal fusions with different neuron-specific motifs for subcellular targeting during their transient expression in cultured neurons.

Confocal imaging of cultured neurons transiently transfected by calcium-phosphate precipitation with N-terminal fusions of YTnC and GCaMP6s indicated that the Mito-YTnC, IMS-YTnC and ER-YTnC fusions successfully targeted YTnC into respective neuronal compartments (Fig. 7 and Supplementary Fig. 18). Fusions of GCaMP6s with the Mito and IMS were primarily mislocalized in the cytoplasm of neurons or exhibited a high level of cytoplasmic mislocalization, respectively. Similar to the data concerning the expression of indicators in HeLa cells, the ER-GCaMP6s fusion was non-fluorescent in neurons, as well as the N-terminal fusions of both GCaMP6s and YTnC with PSD95-FingR^27^ (Supplementary Fig. 19). The N-terminal fusions of YTnC and GCaMP6s with Homer targeted both indicators to the synapses inefficiently, and the majority of indicators were cytoplasmically mislocalized (Supplementary Fig. 18).

We further compared the ΔF/F responses of the YTnC and GCaMP6s N-terminal fusions during the spontaneous activity of neuronal cultures transduced with the mixture of rAVV particles carrying green indicator and red R-GECO1 indicator for normalization. The normalized ΔF/F responses of the YTnC indicator in the fusion with the Mito, IMS and Homer were statistically similar to the ΔF/F values for the same GCaMP6s fusions (Supplementary Table 7). The normalized ΔF/F responses of the YTnC indicator in the fusion with the Mito and Homer or IMS were similar or 1.7-fold (p < 0.01) lower than the normalized ΔF/F values for NES-YTnC, respectively. The normalized ΔF/F responses of the GCaMP6s indicator in the fusion with the Mito, IMS and Homer were similar to the ΔF/F values for the non-fused GCaMP6s indicator. Thus, during the spontaneous activity of neuronal cultures, the YTnC indicator in the N-terminal fusions tested demonstrates an ΔF/F response similar to the response for the GCaMP6s in the same fusions.

Overall, taking into account the similar ΔF/F responses but better localization of YTnC in mitochondria compared with GCaMP6s, YTnC is an indicator of choice for calcium transient studies in the mitochondria of neurons.

We subsequently created fusions of the YTnC and GCaMP6s indicators with the cytoplasmic C-terminal end of voltage-gated K^+^ channel 2.1 (KV2.1 motif), the myosin-binding domain of melanophilin (MBD or MLPH motif), the ankyrin-binding domain from voltage-gated Na^+^ channel 1.6 (AIS or Nav1.6 motif) and the N-terminal end from glutamate receptor kainate type2 KA2 protein (KA2 motif) (Supplementary Table 4). The KV2.1, MBD, AIS and KA2 motifs were shown to successfully target ChR2-GFP or CoChR-GFP fusions to the soma and proximal dendrites, the soma and dendrites, the axon initial segment, and the soma of neurons, respectively^28,29^. According to the confocal imaging of the cultured neurons, fusion of the C-terminal end of the YTnC and GCaMP6s indicators with neuron-specific KV2.1, MBD, and AIS motifs did not significantly affect their brightness in cultured neurons transiently transfected by calcium-phosphate precipitation with respective fusions (Supplementary Fig. 20). The exception was the KA2 motif that substantially reduced the brightness of both indicators in neurons. For all motifs examined, the expression of both YTnC and GCaMP6s indicators was not restricted to the soma and proximal dendrites (KV2.1), soma and dendrites (MBD), axon initial segment (AIS) or soma (KA2) as anticipated (Supplementary Fig. 20). We concluded that the KV2.1, MBD, AIS and KA2 motifs cannot efficiently target the YTnC and GCaMP6s indicators to the subcellular sites of neurons.

We subsequently compared the ΔF/F responses of the YTnC and GCaMP6s indicators in C-terminal fusions with KV2.1, MBD, AIS and KA2 motifs during the spontaneous activity of neuronal cultures transduced with the mixture of rAAV particles carrying green indicator and control R-GECO1. The YTnC and GCaMP6s indicators in the same fusions showed statistically similar normalized ΔF/F responses during spontaneous neuronal activity (Supplementary Table 7). Compared with NES-YTnC, the YTnC in the C-terminal fusions with the MBD, AIS and KA2 motifs demonstrated similar normalized ΔF/F response values, with the exception of fusion with the KV2.1 motif where its ΔF/F response was decreased by 1.5-fold (p < 0.01). The GCaMP6s fusions with the MBD and KA2 motifs had normalized ΔF/F response values similar to the response of the non-fused GCaMP6s. However, the KV2.1 and AIS motifs reduced the GCaMP6s ΔF/F response by 1.9- and 1.6-fold (p = 0.024), respectively, compared with the non-fused GCaMP6s. Thus, during the spontaneous neuronal activity, the C-terminal motifs did not show an impact on the ΔF/F responses of the YTnC and GCaMP6s indicators or worsened them.

Overall, the C-terminal neuron-specific motifs examined in this study could not efficiently localize the YTnC and GCaMP6s indicators specifically in the respective subcellular sites of neurons, and some of them affected the brightness or ΔF/F response of the indicators.

### Comparison of cytotoxicity and distribution of the YTnC and GCaMP6s indicators in neurons

The YTnC indicator binds twice less calcium ions than GCaMP6f and has an “inert” TnC Ca^2+^-binding domain that does not have proteins-partners in neurons; thus, we assumed that YTnC may possess less cytotoxicity than GCaMP6f. Suggesting that the cytotoxic expression of the indicator in neurons may affect their glial surrounding, we compared the number of microglia and astrocytes in the dentate gyrus area of the hippocampus around neurons that expressed the YTnC and GCaMP6f indicators in the areas distant from the injection site (Supplementary Fig. 21a). We determined that the number of cells immunohistochemically stained for ionized calcium-binding adapter molecule 1 (IBA1), a marker of microglial cells, was the same for the YTnC (averaged across 5 mice) and GCaMP6f (averaged across 5 mice) indicators (Supplementary Fig. 21b). The number of cells stained for glial fibrillary acidic protein (GFAP), a marker for astrocytes, was also the same. Thus, we did not identify a difference in the glial surrounding of neurons that expressed the YTnC and GCaMP6f indicators assuming similar cytotoxicity for the indicators.

As YTnC has a smaller size than the GCaMP6f indicator, we reasoned that YTnC may have an improved passive diffusion to subcellular compartments of neurons, such as axons and spines. With this idea, we compared the distribution of the YTnC and GCaMP6f indicators in neurons of the dentate gyrus region of the hippocampus using immunohistochemistry. According to confocal imaging of brain slices stained with antibodies against GFP, we imaged axons and spines with only slightly better contrast in the case of the YTnC indicator than in the case of the GCaMP6f indicator (Supplementary Fig. 22 and 23). The YTnC indicator had both cytoplasmic and nuclear localization in contrast to GCaMP6f, which had dim staining inside nuclei. Thus, despite the smaller size of the YTnC indicator vs GCaMP6f, we did not identify a significant difference in their distribution in the subcellular compartments of neurons with the exception of nuclei.

## Summary

In summary, using directed molecular evolution in bacteria, we developed a new genetically encoded green calcium indicator, called YTnC, that had a NTnC-like design and positive fluorescence response; YTnC had substantially increased the fluorescence ΔF/F response and had faster kinetics than NTnC.

We first characterized the main properties of the newly developed YTnC indicator as a purified protein *in vitro*. YTnC had a positive Ca^2+^ response, i.e., its green fluorescence increased following the binding of Ca^2+^ ions, in contrast to the NTnC progenitor. Compared with other commonly used indicators, its size was ~100 amino acids smaller and it had half the number of Ca^2+^-binding sites, similar to the NTnC indicator (Fig. 1a and Supplementary Fig. 1a). In the presence of Mg^2+^ ions *in vitro*, the YTnC indicator had a fluorescence ΔF/F response that was 3-fold higher than that of the NTnC indicator. YTnC had a significantly lower brightness and photostability (13.6- and 3.6-fold, respectively) and a faster maturation rate than NTnC *in vitro*. The limited brightness of YTnC as a purified protein *in vitro* correlated with its relative brightness in mammalian cells. Moreover, in HeLa cells, the YTnC indicator in fusion with P2A-mCherry had a 1.6- and 3.3-fold lower brightness than EGFP and NTnC in the same fusion, respectively (Table 1 and Supplementary Fig. 4b). Under the same conditions, NES-YTnC_sat_ had a 4.7-fold lower brightness than non-fused GCaMP6s_sat_ (Supplementary Table 5). We assume that this relative improvement in the YTnC brightness in mammalian cells may be connected with its better folding and maturation inside mammalian cells, as well as its higher resistance to degradability by the machinery of mammalian cells. Notably, the low brightness and photostability of YTnC as a pure protein did not constrain its application for short-term confocal imaging of neuronal cultures and one- and two-photon *in vivo* experiments with YTnC calcium imaging in the mouse brain.

According to stopped-flow experiments *in vitro*, YTnC had Ca^2+^-dissociation kinetics that were 3.8-fold faster and 2.2-fold slower than those of NTnC and GCaMP6f, respectively (Fig. 2). Depending on the Ca^2+^ concentration, which was in the range of 300–1300 nM, the Ca^2+^-association kinetics of YTnC were 8.4–1.3-fold faster or 1.09–1.3-fold slower than those of GCaMP6f and NTnC, respectively. Moreover, the fast *in vitro* kinetics of YTnC enabled it to serve as an indicator to reveal spontaneous neuronal calcium activity with a resolution of spikes similar to that of the R-GECO1 indicator (Fig. 3e and Supplementary Fig. 5).

We expressed the YTnC indicator and characterized its response in cultured mammalian and neuronal cells. YTnC reliably responded to the variations in the Ca^2+^ ion concentration induced by ionomycin in mammalian cells, and according to the ΔF/F values, YTnC outperformed the NTnC indicator by 4.9-fold and approximately reached the ΔF/F values of R-GECO1 (Fig. 3b,c). YTnC visualized the spontaneous activity of neurons in dissociated neuronal cultures with approximately the same spike rise/decay kinetics as R-GECO1, but with a 3.6-fold higher or similar ΔF/F dynamic range compared to that of NTnC or R-GECO1 and GCaMP6s GECIs, respectively (Fig. 3e,f and Supplementary Table 7). Improvements in the ΔF/F values for YTnC were closer to the same parameters for the purified YTnC and NTnC proteins *in vitro* in the presence of 1 mM Mg^2+^ ions (Table 1). The ΔF/F response of YTnC in fusion with R-GECO1-P2A during the stimulation of neuronal cultures with an external electric field was 1.9-fold higher or 5.6- and 4.5-fold lower than that of GCaMP6s in the same fusion with R-GECO1-P2A or non-fused GCaMP6s and R-GECO1 indicators, respectively. These data assume that the performance of calcium indicators in neurons may be affected by the N-terminal fusion even through a P2A-cleavable peptide.

Using patch-clamp recording, we showed that YTnC linearly responded to 2–100 APs (Fig. 4b); however, the NTnC and GCaMP6s indicators showed saturations above 10 and 50 APs, respectively^2^. This linearity border correlated well with the *in vitro* K_d_ values for these indicators, which were in the order of NTnC < GCaMP6s < YTnC. Because of this non-linearity, in the range of 2–10 APs, YTnC showed slightly larger ΔF/F values, 1.1–1.3-fold, than NTnC, and in the range of 15–20 APs, this difference increased to 2.2–2.9-fold^2^. Thus, YTnC is undoubtedly preferable over the NTnC indicator for monitoring neuronal calcium activity because of its more linear and higher amplitude response. However, the ΔF/F response of the YTnC indicator in patched neurons in the range of 2–100 APs was 1.5–2.6-fold lower than that of GCaMP6s (Fig. 4), which translated into a lower ΔF/F response relative to that of GCaMP6s in the soma of neurons *in vivo*.

We further validated the performance of the YTnC indicator in a classical *in vivo* application, using two-photon imaging of neuronal calcium activity in the L2/3 primary visual cortex (V1) of YTnC-infected mice with a fixed head. Using the YTnC indicator, we identified spontaneous and drifting grating-evoked neuronal activity in the soma of neurons located in the mouse visual cortex (Fig. 5). The averaged grating-specific ΔF/F response of the YTnC indicator was 1.9-fold lower than that of the GCaMP6s indicator (Fig. 5b,c). The YTnC indicator had spontaneous activity in the spines of neurons, with an ΔF/F response similar to that of GCaMP6s (Supplementary Fig. 9). In a similar brain structure with a similar stimulus and two-photon imaging conditions, the YTnC indicator had a 3-fold larger average grating-evoked ΔF/F response in the soma than NTnC (the latter had an average grating-evoked ΔF/F response of 20 ± 6%)^2^. Thus, the YTnC indicator could be successfully used for *in vivo* two-photon imaging experiments, and it significantly outperformed the NTnC indicator in the soma or was similar to the GCaMP6s indicator in the soma or spines during spontaneous activity or inferior to GCaMP6s in soma during grating-specific activity in this type of *in vivo* application.

We also probed the YTnC indicator for its ability to image neuronal calcium activity in the hippocampus of freely moving mice using an nVista miniscope. In the hippocampus of freely moving mice, the YTnC indicator had a similar and 1.4-fold lower averageΔF/F response than the GCaMP6f and GCaMP6s indicators, respectively (Fig. 6). These *in vivo* responses were in accordance with the similar K_d_ values for the YTnC and GCaMP6f indicators and the ~2-fold lower K_d_ value for GCaMP6s determined *in vitro* in the presence of 1 mM Mg^2+^ (Table 1)^5^. The average ΔF/F response of the NTnC indicator in the visual cortex of freely moving mice was 2.5-fold lower than that of GCaMP6s^2^. Under similar conditions, the average GCaMP6s response in the hippocampus was slightly lower, 1.3-fold, than that in the visual cortex, i.e., 0.023 ± 0.03 vs 0.03 ± 0.02, respectively. Taking into account this difference, in *in vivo* experiments with a nVista miniscope, the YTnC indicator demonstrated an average ΔF/F response that was 1.7-fold larger than that of the NTnC indicator. The ΔF/F response and SNR of the YTnC indicator were also 1.6- and 2.1-fold larger than those for the recently published iYTnC2 indicator under the same conditions^30^. Using the YTnC indicator, we showed place specific neuronal activity in the hippocampus of freely moving mice during the exploration of a novel context. Thus, compared with the NTnC and iYTnC2 indicators, YTnC had a better response during *in vivo* tasks with nVista miniscope. Overall, the YTnC indicator is currently the best NTnC-like GECI for *in vivo* calcium imaging experiments; however, it remains suboptimal compared to the GCaMP6s indicator, which prompted us to search for other applications in which the YTnC indicator would be advantageous compared with GCaMP6s.

We determined that YTnC outperformed the GCaMP6s indicator in the presence of N-terminal fusions, which targeted it to the lumen or the IMS of the mitochondria or ER, in terms of proper localization, brightness and ionomycin-induced ΔF/F response in cultured HeLa cells (Fig. 7 and Supplementary Tables 4 and 5). The thapsigargin-induced ΔF/F responses of the YTnC in the lumen and IMS of the mitochondria were 2.2-fold larger than and similar to the respective responses of GCaMP6s in the same organelles of HeLa cells (Supplementary Table 6), respectively. The YTnC indicator N-terminally targeted to the lumen or IMS of the mitochondria or ER also demonstrated better localization than GCaMP6s in neurons (Fig. 7). The YTnC indicator localized in the lumen of mitochondria showed spontaneous activity of neurons with a similar ΔF/F response as that of GCaMP6s (Supplementary Table 7). The C-terminal KV2.1, MBD, AIS and KA2 neuron-specific motifs did not efficiently target both the YTnC and GCaMP6s indicators to the subcellular sites of neurons and, in some cases, worsened the ΔF/F response of the indicators (Supplementary Fig. 20 and Supplementary Table 7). The better tolerance of the YTnC indicator to N-terminal fusions compared with GCaMP6s may be related to their different designs. The NTnC-like design has both N- and C-termini that are the same as in the EGFP protein; however, the GCaMP-like design suggests the presence of a short M13-like peptide on its N-terminus through which the N-terminally fused partner-protein may affect the properties of the GCaMP indicator.

Despite the decreased number of Ca^2+^-binding sites and “inert” TnC domain, the YTnC indicator did not exhibit an advantage over GCaMP6s in its expected lower cytotoxicity because we did not identify a difference in the glia surrounding neurons expressing the YTnC or GCaM6s indicators in the dentate gyrus area of the hippocampus (Supplementary Fig. 21). Despite the smaller size of YTnC, we did not identify a significant difference between the distribution of the YTnC and GCaMP6f indicators in the branches and spines of neurons (Supplementary Fig. 22 and 23).

We anticipate that further enhancement of the brightness and ΔF/F sensitivity of the YTnC indicator would bring it to the next performance level, superior to that of GECIs with a conventional design, and would provide calcium imaging tools with additional advantages of the NTnC-like design, such as efficient targeting to cellular compartments.

## Materials and Methods

Mutagenesis and library screening, protein purification and characterization, stopped-flow fluorimetry, mammalian plasmid construction, cell culture and transfection, rAAV particle production and isolation, mammalian live-cell imaging, isolation, transduction, and imaging of neuronal cultures, whole-cell electrophysiology and calcium imaging were performed as described in ref.^30^ with modifications (Supplementary Table 8 and Supplementary Methods).

Surgeries for V1 *in vivo* two-photon imaging and two-photon *in vivo* mouse imaging in V1 were performed on 14 and 10 C57BL/6 mice (Jackson Laboratory, USA) infected with rAAV particles carrying AAV-*CAG*-NES-YTnC or AAV-*CAG*-GCaMP6s, respectively, and through an installed cranial window as described in ref.^2^ with modifications (Supplementary Methods).

Animals and surgeries for imaging with an nVista HD miniature microscope and Ca^2+^ *in vivo* imaging with the nVista HD miniature microscope were accomplished on twelve adult male C57BL/6 mice (Jackson Laboratory, USA), aged 20 weeks at the beginning of the experiments, and transduced with rAAV particles carrying AAV-*CAG*-NES-YTnC, AAV-*CAG*-GCaMP6f, or AAV-*CAG*-GCaMP6s as described in ref.^30^ with modifications (Supplementary Methods).

### Immunohistochemistry

After the experiments, the mice were anesthetized and transcardially perfused with 30 mL of phosphate buffered saline (PBS) and 30 mL of 4% paraformaldehyde in PBS (pH 7.4). The extracted brains were further post-fixed in 4% paraformaldehyde in PBS overnight at 4 °C and stored in PBS with 0.1% sodium azide at 4 °C until sectioning. The brains were sagittally or coronally sectioned at a thickness of 50 µm with a vibratome (Leica). After rinses with PBS, the sections were incubated for blocking and permeabilization in PBS with 2% Triton X-100 and 5% goat serum at room temperature for 2 h. After rinses with washing solution (PBS with 0.2% Triton X-100), the sections were incubated at 4 °C overnight in antibody solution (PBS with 0.2% Triton X-100 and 5% goat serum) that contained primary antibodies: chicken anti-GFP (1:500 dilution; GFP-1020, Aves Labs), mouse anti-NeuN (1:500 dilution, AB-377, Millipore), rabbit anti-glial fibrillary acidic protein (GFAP, 1:500 dilution; 180063, Invitrogen) and rabbit anti-Iba1 (1:1000 dilution; 019-19741, Wako). The primary antibody reaction was extended for 2 h at room temperature, and the sections were rinsed with washing solution. The sections were subsequently incubated for 2 h at room temperature with Alexa Fluor (AF) fluorescent dye-conjugated goat secondary antibody AF-488, AF-568, or AF-647 (1:500 dilution; Invitrogen). After rinses with washing solution, the sections, mounted on poly-L-lysine-coated slide glasses, were cover-slipped over fluorescence mounting media (Dako) for confocal microscopy.

The mosaic Z-stack images were collected with the spinning disk confocal microscope Andor Revolution WD (Andor Technology, UK) and analyzed using Imaris (Bitplane AG, Switzerland) software. Representative images were imported into ADOBE PHOTOSHOP 13.0 (Adobe Systems) and were minimally processed to adjust the brightness, contrast, and background.

### Comparison of cytotoxicity and distribution of YTnC and GCaMP6f indicators in neurons

To compare the cytotoxicity effects and distribution of the YTnC and GCaMP6f indicators in neurons, rAAV viral particles carrying AAV-CAG-NES-YTnC or AAV-CAG-NES-GCaMP6f were injected as previously described into the left and right hemispheres of the same mouse. The animals were perfused four weeks after the injections. The sagittal sections were immunochemically stained as previously described and captured under a 60x objective lens with the scanning confocal microscope Olympus Fluoview10i (Olympus, Japan). The quantification was produced in Imaris (Bitplane AG, Switzerland) software on three sections for each hemisphere and animal into two 210 × 210 μm frames using a blind method. The total numbers of astrocytes (GFAP^+^) and microglia (Iba^+^) were counted. The mean number of cells per frame was obtained by successively averaging the number of cells in frames, sections, and animals.

### Statistics

To estimate the significance of the difference between two values, we used the Mann-Whitney Rank Sum Test and provided p-values (throughout the text in the brackets) calculated for the one-tailed hypothesis.

### Ethical approval and animal care

All methods for animal care and all experimental protocols were *approved* by the National Research Center “Kurchatov Institute” Committee on Animal Care (protocol No. 1, 09 July 2015) and were *in accordance* with the *Russian* Federation Order Requirements N *267* МЗ and the National Institutes of Health Guide for the Care and Use of Laboratory Animals. Nineteen and five C57BL/6 mice were used in this study, ages ~2-4 months and P0-P2 old, respectively. Mice were used without regard to gender.

## Electronic supplementary material

Supplementary Information

Supplementary Video 1

Supplementary Video 2

Supplementary Video 3

## Data Availability

All data generated or analyzed during this study are included in this published article (and its Supplementary Information files).
