# Supplementary material for: NTnC-like genetically encoded calcium indicator with a positive and enhanced response and fast kinetics
Source: Sci Rep. 2018 Oct 15;8:15233. doi: 10.1038/s41598-018-33613-6 (PMC6189086; doi:10.1038/s41598-018-33613-6)
Supplement: Supplementary file 1 — Supplementary Information [file 41598_2018_33613_MOESM1_ESM.docx]

## NTnC-like genetically encoded calcium indicator with a positive and enhanced response and fast kinetics

**Natalia V. Barykina,^1,2 *^ Danila A. Doronin,^1 *^ Oksana M. Subach,^1,3^ Vladimir P. Sotskov,^1,4^ Viktor V. Plusnin,^1,3^ Olga A. Ivleva,^1,4^ Anna M. Gruzdeva,^2,3,4^ Tatiana A. Kunitsyna,^3^ Olga I. Ivashkina,^2,3,4^ Alexander A. Lazutkin,^1,2^ Aleksey Y. Malyshev,^5^ Ivan V. Smirnov,^5,6^** **Anna M. Varizhuk,^7,8^ Galina E. Pozmogova,^7^ Kiryl D. Piatkevich,^9^ Konstantin V. Anokhin,^2,3,4^ Grigori Enikolopov,^1,10,11^ ** and Fedor V. Subach^1,3^ ****

^1^Moscow Institute of Physics and Technology, Dolgoprudny 141701, Russia

^2^P.K. Anokhin Institute of Normal Physiology, Moscow 125315, Russia

^3^National Research Center “Kurchatov Institute”, Moscow 123182, Russia

^4^Lomonosov Moscow State University, Moscow, 119991, Russia

^5^Institute of Higher Nervous Activity and Neurophysiology of RAS, Moscow, 117485, Russia

^6^Pirogov Russian National Research Medical University, Moscow, 117997, Russia,

^7^Federal Research and Clinical Center of Physical-Chemical Medicine of Federal Medical Biological Agency, Moscow 119435, Russia

^8^Engelhardt Institute of Molecular Biology RAS, Moscow 119991, Russia

^9^MIT Media Lab, Massachusetts Institute of Technology, Cambridge, MA, USA

^10^Department of Anesthesiology, Stony Brook University Medical Center, NY 11794, USA

^11^Center for Developmental Genetics, Stony Brook University, NY 11794, USA

*** These authors contributed equally to this work.

** Correspondence and requests for materials should be addressed to F.V.S. (email: [subach.fv@mipt.ru](mailto:subach.fv@mipt.ru)) or G.N.E. (email: Grigori.Enikolopov@stonybrookmedicine.edu)

**Supplementary Information**

**Supplementary Figures 1-25, Videos 1-3, Tables 1-8, and Methods**

**Supplementary Figure 1. Schematic representation of composition and function of FRET-based, cpFP-based, and NTnC-like indicator families in the Ca^2+^-bound state.**

**Supplementary Figure 2. Alignment of the amino acid sequences for the original library, YTnC and NTnC calcium indicators.**

**Supplementary Figure 3. Alignment of the amino acid sequences of EGFP and EYFP.**

**Supplementary Figure 4. Relative brightness of the NTnC, YTnC and EGFP proteins in mammalian cells.**

**Supplementary Figure 5. Confocal images of fluorescence and morphology of neurons and traces for spontaneous activity of neurons co-expressing YTnC and R-GECO1 indicators.**

**Supplementary Figure 6. Estimation of YTnC photostability in neurons from dissociated culture under confocal microscope**

**Supplementary Figure 7. Electric circuit of neuronal stimulator.**

**Supplementary Figure 8.** **Fluorescence changes in cultured neurons co-expressing the YTnC and R-GECO1 indicators to intracellularly induced train of 10 APs.**

**Supplementary Figure 9. Comparison of the YTnC and GCaMP6s indicators in the spines of neurons during spontaneous activity in the L2/3 of visual mice cortex.**

**Supplementary Figure 10. Confocal images of brain slices from YTnC- or GCaMP6f-expressing mice immunohistochemically stained with antibodies against GFP, NeuN, Iba1 or GFAP.**

**Supplementary Figure** **11.** **Spike detection scheme.**

**Supplementary Figure** **12.** **Comparison of the YTnC and GCaMP6s indicators photostability during single (a, b) and multiple 10-min imaging sessions (c) of mouse hippocampus using NVista miniscope.**

**Supplementary Figure 13. Localization of the YTnC and GCaMP6s calcium indicators targeted to the lumen and IMS of mitochondria and ER of the HeLa cells.**

**Supplementary Figure 14. Localization of the YTnC and GCaMP6s calcium indicators targeted to the nucleus and plasma membrane of the HeLa cells.**

**Supplementary Figure 15. Localization of the GCaMP6s calcium indicator targeted to the ER of the HeLa cells after incubation at room temperature.**

**Supplementary Figure 16. Comparison of the YTnC and GCaMP6s calcium indicators response in the lumen and IMS of mitochondria and ER of the HeLa cells.**

**Supplementary Figure 17. Localization of the YTnC and GCaMP6s calcium indicators targeted to the β-actin and α-tubulin microtubules of the HeLa cells.**

**Supplementary Figure 18. Localization of the YTnC and GCaMP6s calcium indicators targeted through their N-terminus to the different compartments of the neuronal cells.**

**Supplementary Figure 19. Localization of the YTnC and GCaMP6s calcium indicators targeted through their N-terminal fusion with PSD95.FingR intrabody to the spines of the neuronal cells.**

**Supplementary Figure 20. Localization of the YTnC and GCaMP6s calcium indicators targeted through their C-terminus to the different compartments of the neuronal cells.**

**Supplementary Figure 21. Comparison of the YTnC and GCaMP6f indicators cytotoxicity in dentate gyrus using immunohistochemistry to Iba1 and GFAP.**

**Supplementary Figure 22. Comparison of the YTnC and GCaMP6f indicators distribution in the cell bodies, axons and spines in dentate gyrus of hippocampus across 4 mice.**

**Supplementary Figure 23. Zoomed comparison of the YTnC and GCaMP6f indicators distribution of in the cell bodies, axons and spines in dentate gyrus of hippocampus across 1 mouse.**

**Supplementary Figure 24. Optical configuration of spinning-disk module CSU-W1 from the Andor confocal imaging system.**

**Supplementary Figure 25. Optical configuration of the two-photon imaging system.**

**Supplementary Video 1**. ***In vivo* visual stimuli evoked neuronal Ca^2+^ activity in the mouse cortex as visualized with the YTnC calcium indicator and two-photon microscopy.**

**Supplementary Video 2. *In vivo* neuronal calcium activity in hippocampus of anesthetized mouse visualized with the YTnC calcium indicator and nVista HD system**.

**Supplementary Video 3. *In vivo* neuronal calcium activity in hippocampus of mouse visualized with the YTnC calcium indicator and nVista HD system synchronized with mouse movement**.

**Supplementary Table 1.** **Characteristics of calcium responses to external field stimulation of neurons expressing the YTnC and GCaMP6s indicators in dissociated neuronal culture.**

**Supplementary Table 2. Characteristics of calcium responses to intracellular stimulation with 10 APs in neurons expressing the YTnC and GCaMP6s indicators in dissociated neuronal culture.**

**Supplementary Table 3. Characteristics of calcium responses in neurons expressing the YTnC, GCaMP6f and GCaMP6s indicators in hippocampus of freely moving mice registered with nVista HD.**

**Supplementary Table 4.** **Sequences of the targeting motifs tested in this study.**

**Supplementary Table 5.** **Comparison of the YTnC and GCaMP6s indicators brightness and response to the addition of ionomycin in different compartments of HeLa cells.**

**Supplementary Table 6.** **Comparison of the YTnC and GCaMP6s indicators brightness and response to the addition of thapsigargin in different compartments of HeLa cells.**

**Supplementary Table 7.** **Comparison of the YTnC and GCaMP6s indicators response during spontaneous activity of neuronal cultures.**

**Supplementary Table 8. List of primers.**

**Supplementary Methods**

**Supplementary References**

**
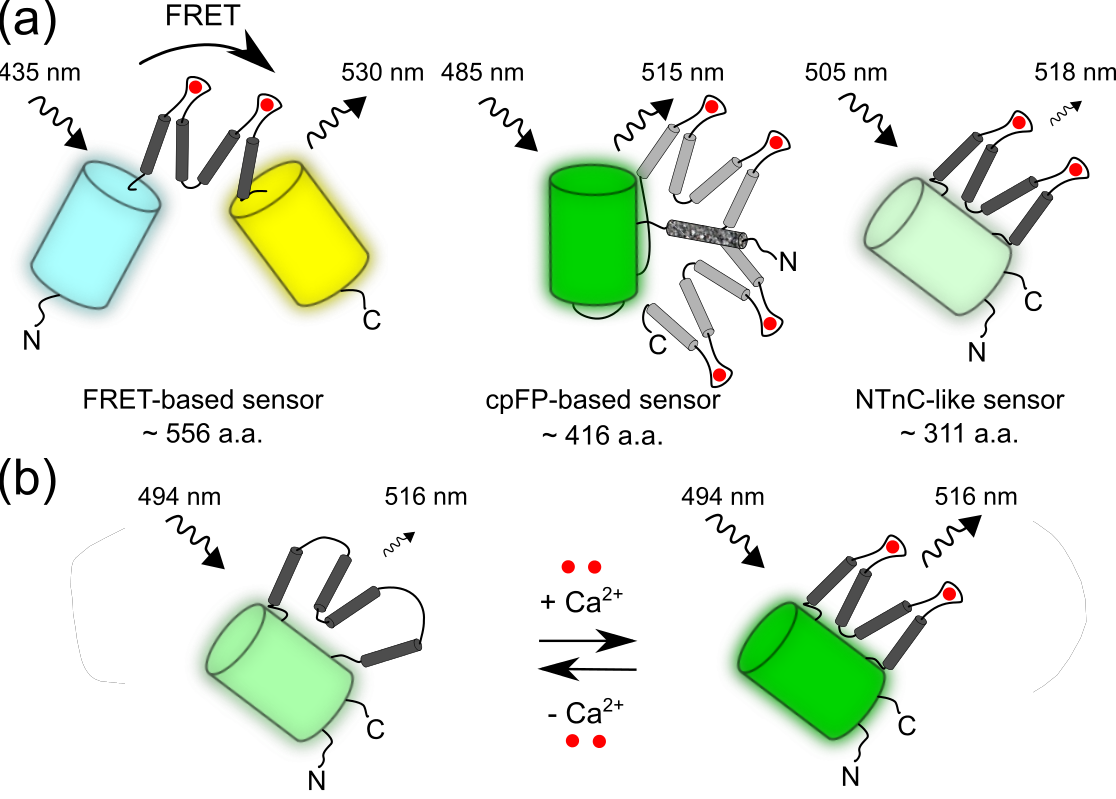
**

**Supplementary Figure 1. Schematic representation of composition and function of FRET-based, cpFP-based, and NTnC-like indicator families in the Ca^2+^-bound state.** **(a)** CFP, GFP, and YFP are shown as cyan, green, and yellow cylinders, respectively, and tsTnC, CaM and M13-peptide are shown in dark grey, light grey, and speckled grey, respectively. **(b)** Schematic representation of the YTnC indicator function. The EYFP fluorescent part is shown as light green or intense green cylinders before or after binding of Ca^2+^ ions, respectively; tsTnC domains are shown as grey small tubes; Ca^2+^ ions are shown as red dots.

-1 10        20        30        40        50

          |       |         |         |         |         |

Library MVSKGEELFTGVVPILVELDGDVNGHKFSVSGEGEGDATYGKLTLKFICTTGKLPVPW

YTnC MVSKGEELFTGVVPILVEMVGDVNGHRFSVSGEGEGDATYGKLTLKLICTTGELPVPW

NTnC    MVSKGEEDNMASLPATHELHIFGSINGVDFDMVGQGSGNPNVGYEELNLKSTKGDLQFSP

        60        70        80        90       100       110

          |    ***  |         |         |         |         |

Library PTLVTTFGYGLQCFARYPDHMKQHDFFKSAMPEGYVQERTIFFKDDGNYKTRAEVKFEGD

YTnC  PTLVTTLGYGLQCFARYPDHMKQHDFFKSAMPEGYVQERTIFFKDDGNYKTRAEVKFEGD

NTnC    WILVPHIGYGFHQYLPYPDGMS-PFQAAMVDGSGYQVHRTVQFEDGASLTVNYRYTYEGS

       120       130       140       150       160       170

         |        |         |         |         |         |

Library TLVNRIELKGIDFKEDGNILGHKLEYNXXXSEEELSECFRTFDKDGDGFIDREEFGGIIR

YTnC TLVNRIELKGFDFKEDGNILGHKLGYNFDLSEEELSECFRTFDKDGDGFIDREEFGGIIR

NTnC HIKGEAQVKGTGFPADGPVMANSLT-AMVPSEEELSECFRTFDKDGDGFIDREEFGGIIR

   180       190       200       210       220       230

          |        |         |         |         |         |

Library LTGEQLTDEDPDEIFGDSDTDKNGRIDFDEFLKMVENVQXX--NSHNVYIMADKQKNGIK

YTnC  LTGEQLTDEDPDEIFGDSDTDKNGRIDFDEFLKMVENVQGV--NSHNVYITADKQKNGIK

NTnC    LTGEQLTDEDPDEIFGDSDTDKNGRIDFDEFLKMVENVQLSMADWCRSKMACP-NDKTLI

          240    250       260       270       280       290

            |      |         |         |         |         |

Library VNFKIRHNIEDGSVQLADHYQQNTPIGDG---PVLLPDNHYLSYQSALSKDPNEKRDHMV

YTnC  AHFKIRHNIEDGSVQLADHYQQNTPIGDG---PVLLPDNHYLRTQSALSKDPNEKRDHMV

NTnC    STLKWSYTTGNGKRYRSTARTTYTFAKPMAANYLKNQP-MYVFRKTELKHS----KTEMD

          300 310

            | |

Library LLEFVTAAGITLGMDELYK

YTnC LLEFVTAAGITHGMDELYK

NTnC    FKEWQKAFTDVMGMDELYK

**Supplementary Figure 2. Alignment of the amino acid sequences for the original library, YTnC and NTnC calcium indicators.** Alignment numbering follows that of YTnC. Residues from fluorescent part buried in β-can are highlighted with green. Residues that are forming chromophore are selected with asterisk. Mutations in YTnC related to the initial library including linkers between fluorescent and indicator parts are highlighted in red. Residues that are forming Ca^2+^-binding loops are highlighted in blue.

10 20 30 40 50

| | | | |

GFP mskgeelftgvvpilveldgdvnghkfsvsgegegdatygkltlkficttg---klpvpwp

EYFP MVSKGEELFTGVVPILVELDGDVNGHKFSVSGEGEGDATYGKLTLKFICTTG---KLPVPWP

60 70 80 90 100 110

| *** | | | | |

GFP TlvttfSygvqcfsrypdhmkqhdffksampegyvqertiffkddgnyktraevkfeGDt

EYFP TLVTTFGYGLQCFARYPDHMKQHDFFKSAMPEGYVQERTIFFKDDGNYKTRAEVKFEGDT

120 130 140 150 160 170

| | | | | |

GFP lvnrielkgidfkedgnilghkleynynshnvyimadkqkngikvnfkirhniedgSVQL

EYFP LVNRIELKGIDFKEDGNILGHKLEYNYNSHNVYIMADKQKNGIKVNFKIRHNIEDGSVQL

180 190 200 210 220 230

| | | | | |

GFP adhyqqntpigdg---pvllpdnhylstqsalskdpnekrdhmvllefvtaagithgmdelyk

EYFP ADHYQQNTPIGDG---PVLLPDNHYLSYQSALSKDPNEKRDHMVLLEFVTAAGITLGMDELYK

**Supplementary Figure 3. Alignment of the amino acid sequences of EGFP and EYFP.** Alignment numbering follows that of avGFP. Residues buried in β-can are shaded. Stars indicate residues that are forming chromophore. 18L, 46F, 52K, 64F, 163V, and 203Y residues of EYFP are highlighted in yellow color and correspond to L18M, F46L, K52E, F64L, V236A, and Y276T mutations in YTnC (Supplementary Figure 2). Site of insertion in the EYFP protein is indicated with arrow.

**
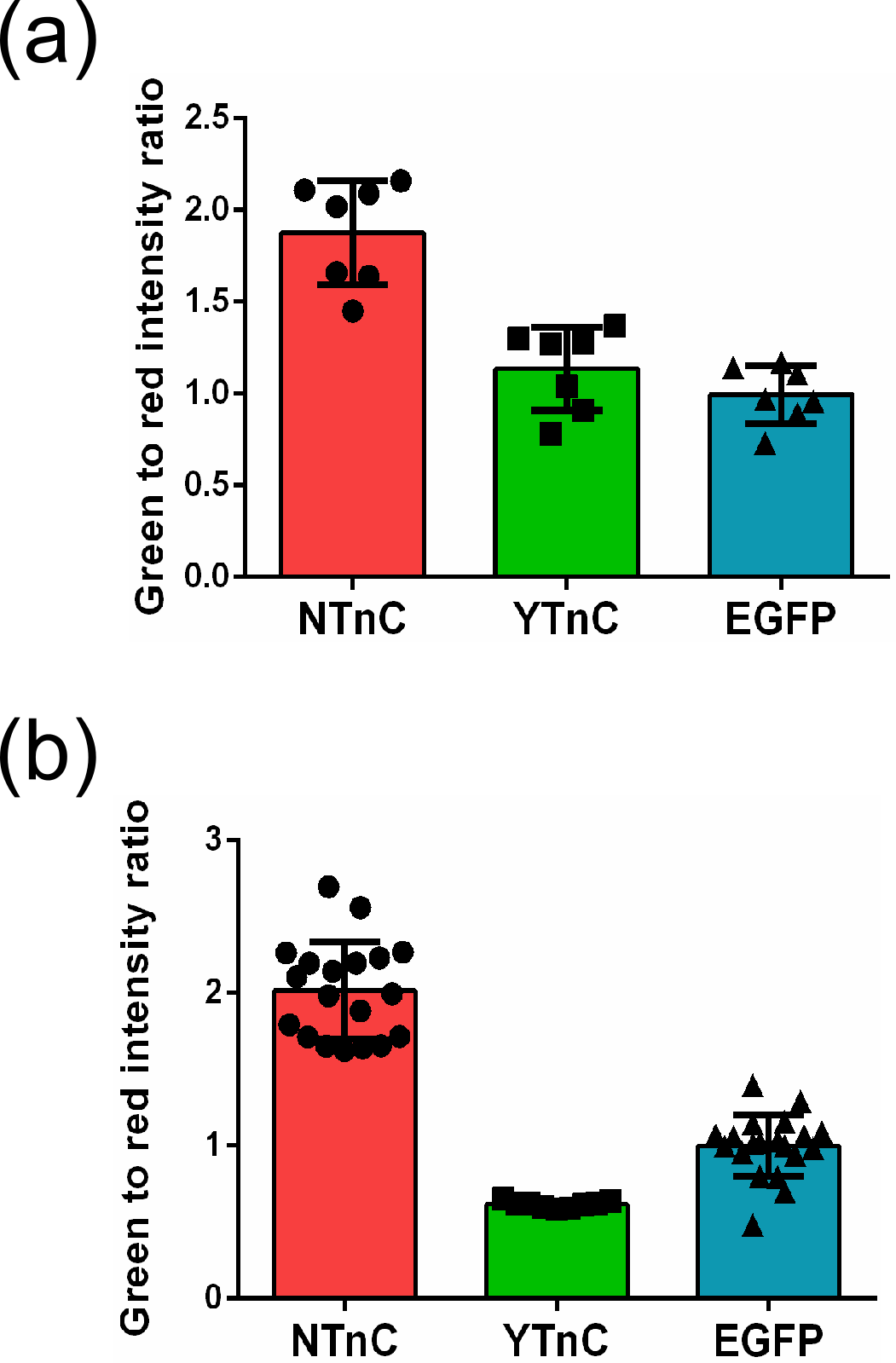
**

**Supplementary Figure 4. Relative brightness of the NTnC, YTnC and EGFP proteins in mammalian cells.** Green fluorescence of calcium indicators and EGFP was normalized to the red fluorescence intensity of mCherry RFP in HeLa Kyoto cells. Imaging was performed within 5 min to avoid cell shrinking. Dots correspond to individual cells, whiskers show standard deviations. **(a)** HeLa cells transiently expressing NES-NTnC-P2A-mCherry, NES-YTnC-P2A-mCherry and NES-EGFP-P2A-mCherry were imaged in DPBS in 2-4 min after addition of 1 mM EDTA/2.5 µM ionomycin, 2 mM CaCl_2_/2.5 µM ionomycin or 2 mM CaCl_2_/2.5 µM ionomycin, respectively, when indicator fluorescence reached plateau. **(b)** HeLa cells transiently expressing NES-NTnC-P2A-mCherry, NES-YTnC-P2A-mCherry and NES-EGFP-P2A-mCherry were imaged in DMEM supplemented with 20mM HEPES, pH 7.40, 10% FBS, Glutamine, 50 U/ml penicillin, and 50 U/ml streptomycin before and after addition of 2.5 µM ionomycin till indicator fluorescence reached plateau.


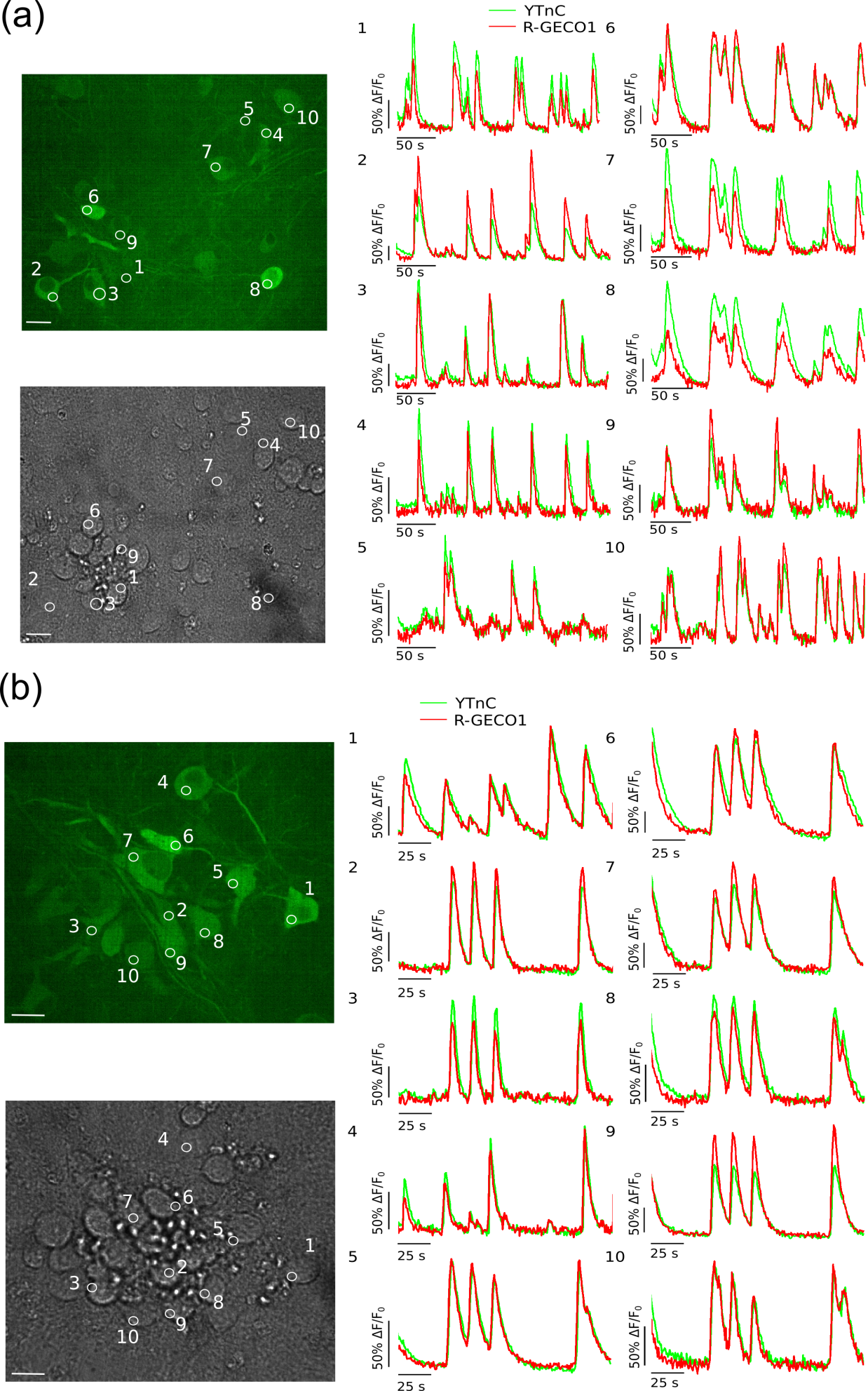


**Supplementary Figure 5. Confocal images of fluorescence and morphology of neurons and traces for spontaneous activity of neurons co-expressing YTnC and R-GECO1 indicators. (a)** and **(b)** panels correspond to the two fields of view of one z-plane on confocal microscope. Upper and lower images on the panels correspond to green fluorescent and transillumination channels. Individual traces are shown for each of the cells in the field of view that revealed activity.


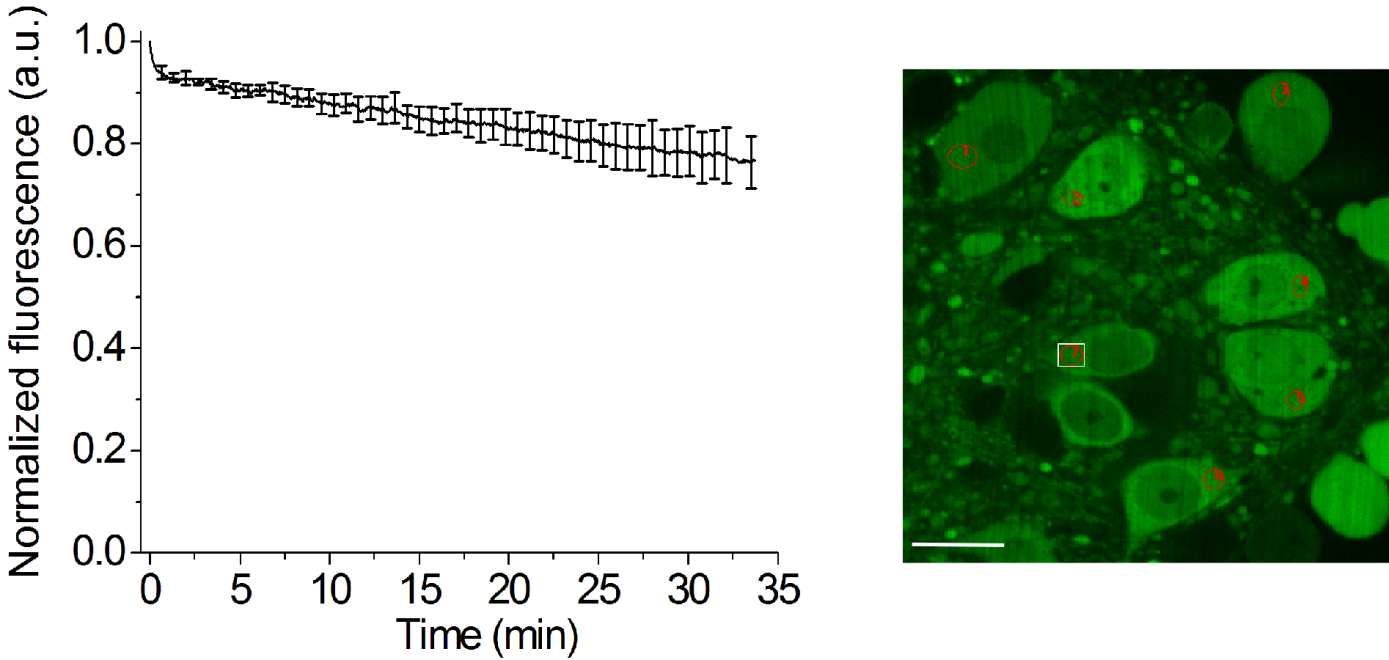


**Supplementary Figure 6. Estimation of YTnC photostability in neurons from dissociated culture under confocal microscope.** Fluorescence time dependence was averaged across areas of 7 cells selected with red color. Error bars show standard deviations for each of the 20^th^ dot.


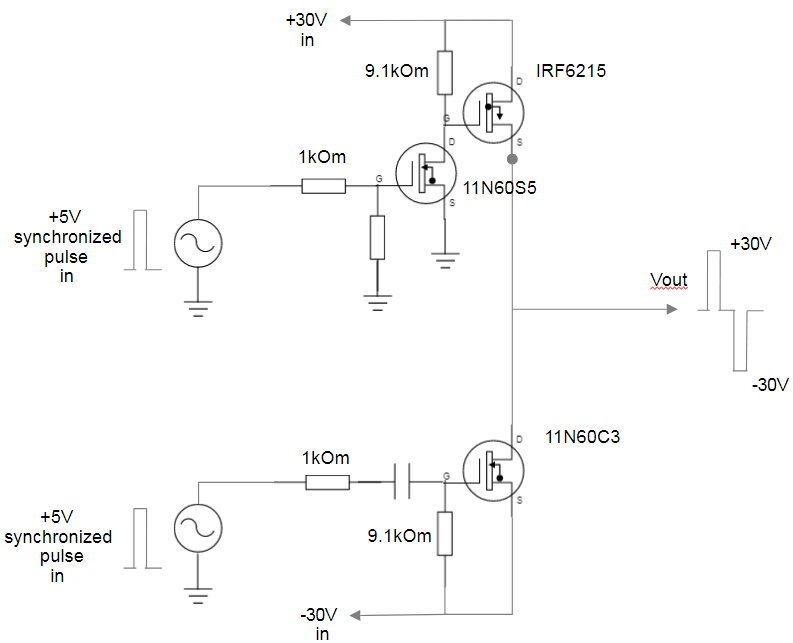


**Supplementary Figure 7. Electric circuit of neuronal stimulator.** Synchronized positive incoming pulses of +5V from generator (Rigol DG1022, Germany) are shifted from each other on 0.46 ms. To make charge balanced biphasic outcoming pulse we slightly shortened the time-phase of the positive pulse. Scheme was drawn online using SmartDraw Software.

**
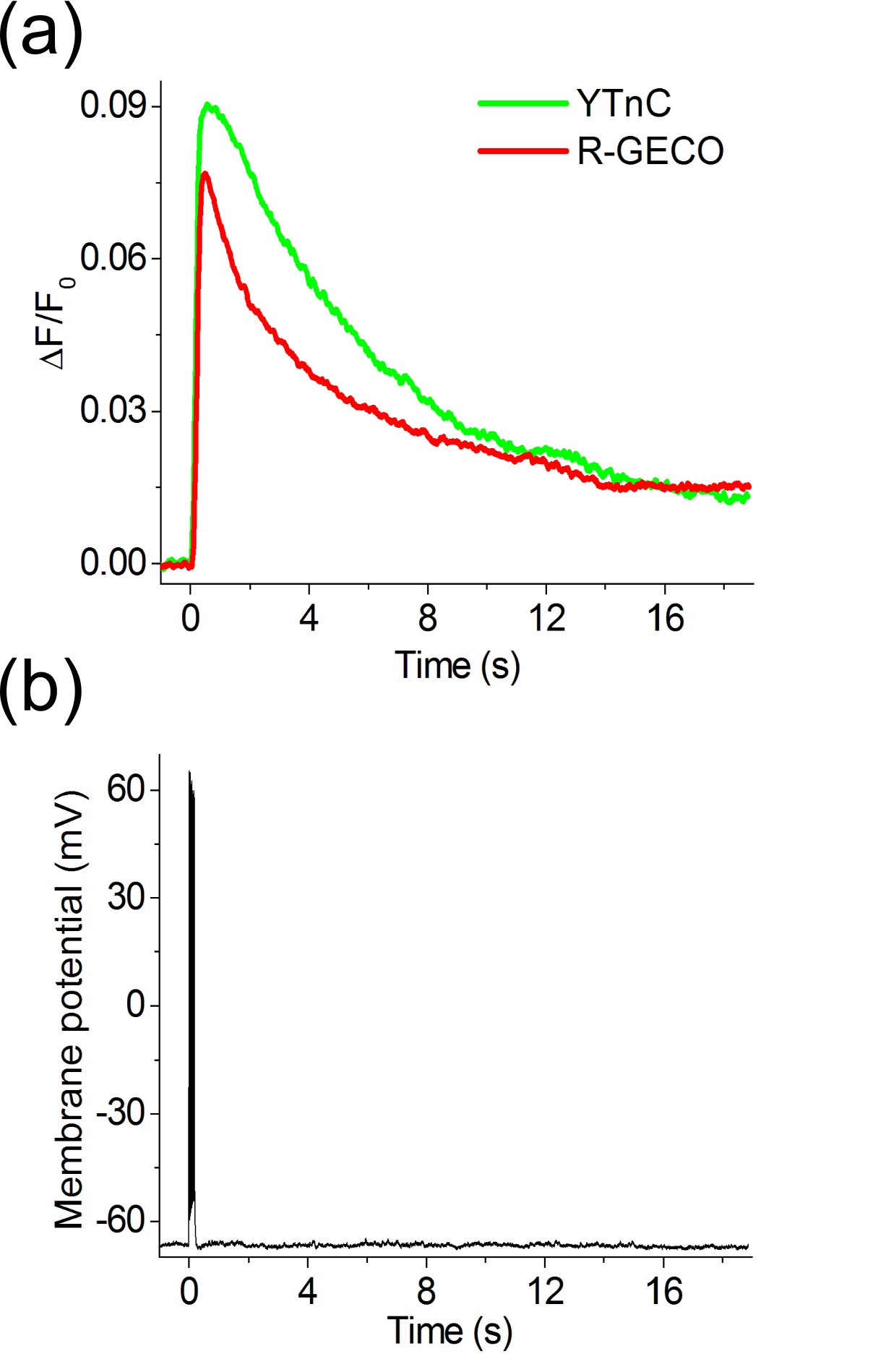
**

**Supplementary Figure 8.** **Fluorescence changes in cultured neurons co-expressing the YTnC and R-GECO indicators to intracellularly induced train of 10 APs.** **(a)** Ca^2+^ responses were averaged across representative recorded neurons in different wells (N=8 for R-GECO and N=9 for YTnC). **(b)** Example of intracellular recording was taken from the one representative cell.

**
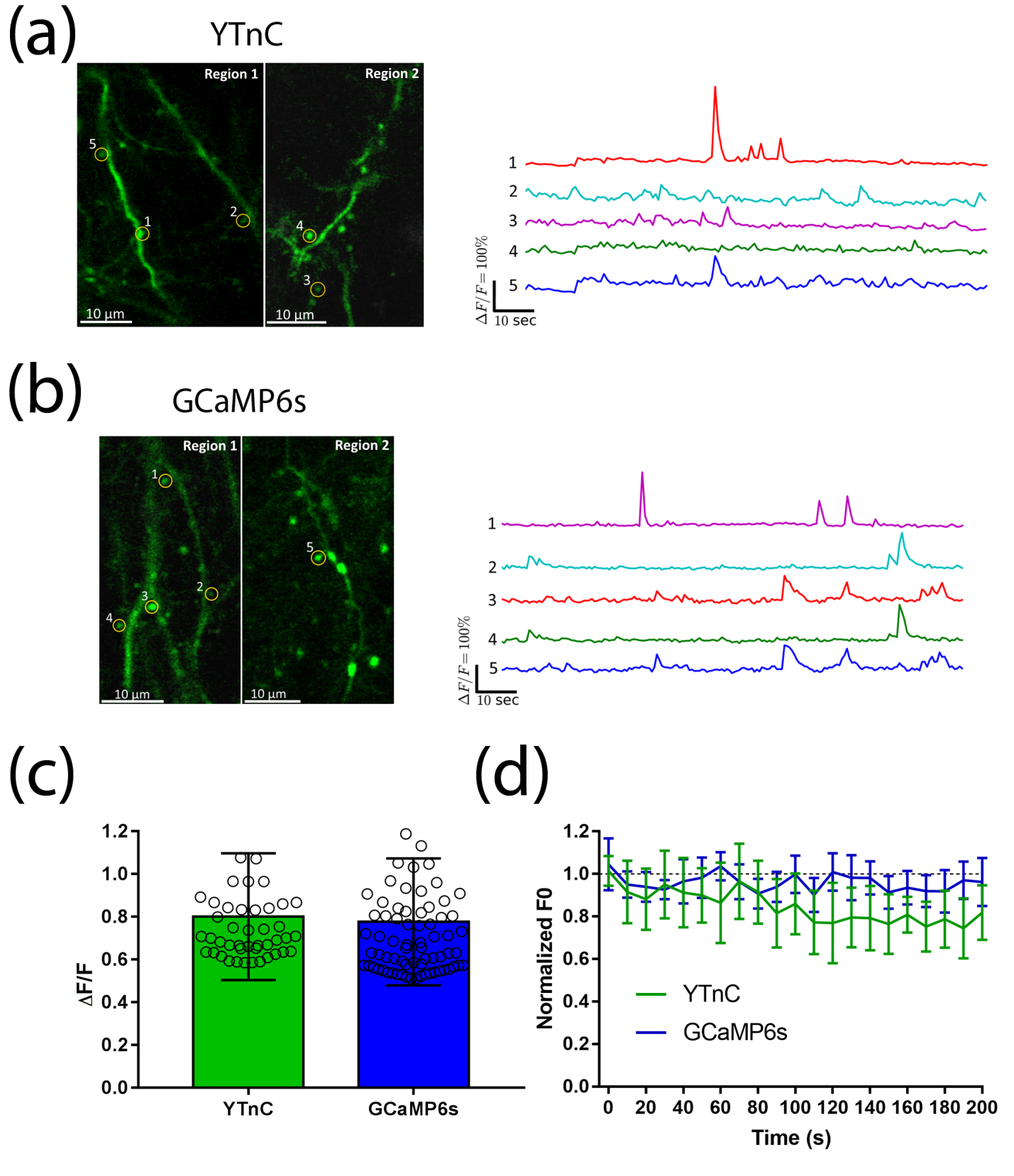
**

**Supplementary Figure 9. Comparison of the YTnC and GCaMP6s indicators in the spines of neurons during spontaneous activity in the L2/3 of visual mice cortex. (a)** Two-photon images of the branches with spines expressing the YTnC indicator from the two regions in the L2/3 of visual mice cortex with traces of calcium activity for the selected spines. **(b)** Two-photon images of the branches with spines expressing the GCaMP6s indicator from the two regions in the L2/3 of visual mice cortex with traces of calcium activity for the selected spines. **(c)** The ΔF/F values for the YTnC and GCaMP6s indicators averaged across 15 and 23 spines, respectively. Error bar, STD. **(d)** Time dependence of the normalized averaged fluorescence intensity during imaging of the spines expressing YTnC and GCaMP6s indicators. Error bar, STD.

**
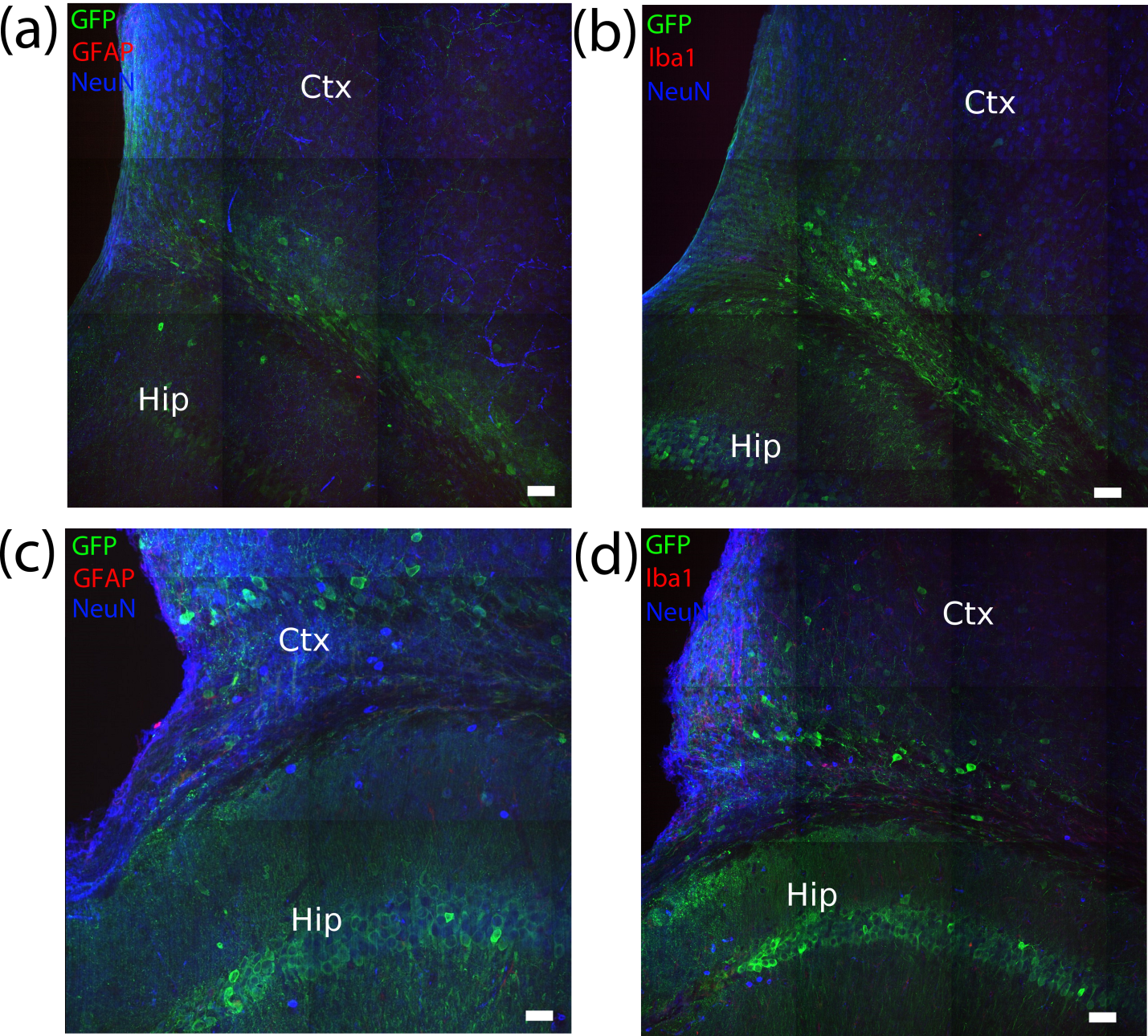
**

**Supplementary Figure 10. Confocal images of brain slices from YTnC- or GCaMP6f-expressing mice immunohistochemically stained with antibodies against GFP, NeuN, Iba1 or GFAP.** Images show an area around site of insertion of lens probe; the site of insertion looks like dark area in a left upper corner. Brain slices of YTnC-**(a, b)** and GCaMP6f**-(c,d)**-expressing mice. Scalebar, 50 µm. Confocal images were acquired using Andor confocal spinning disk system. Ctx – cortex; Hip – hippocampus.


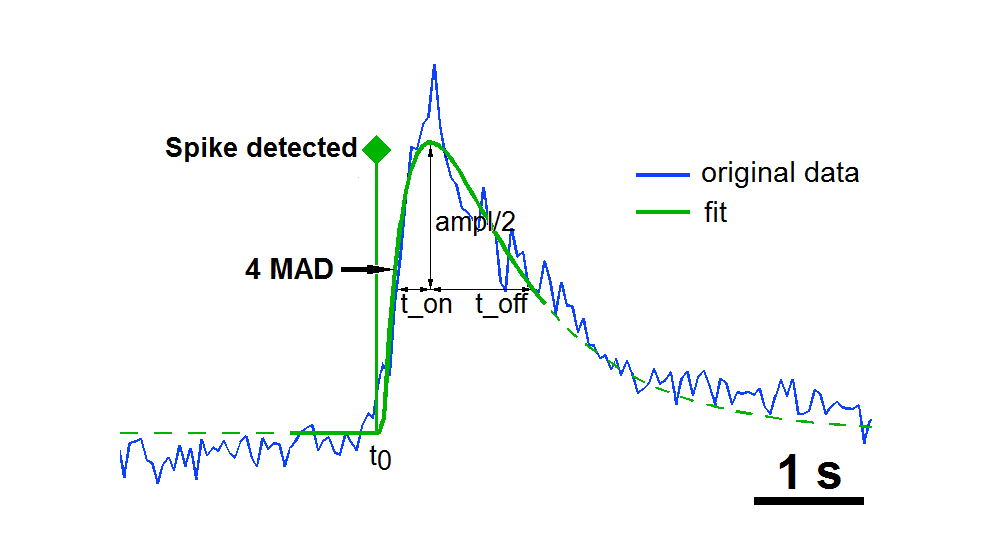
**Supplementary Figure** **11.** **Spike detection scheme.** *t_on* and *t_off* are rise and decay half-times.


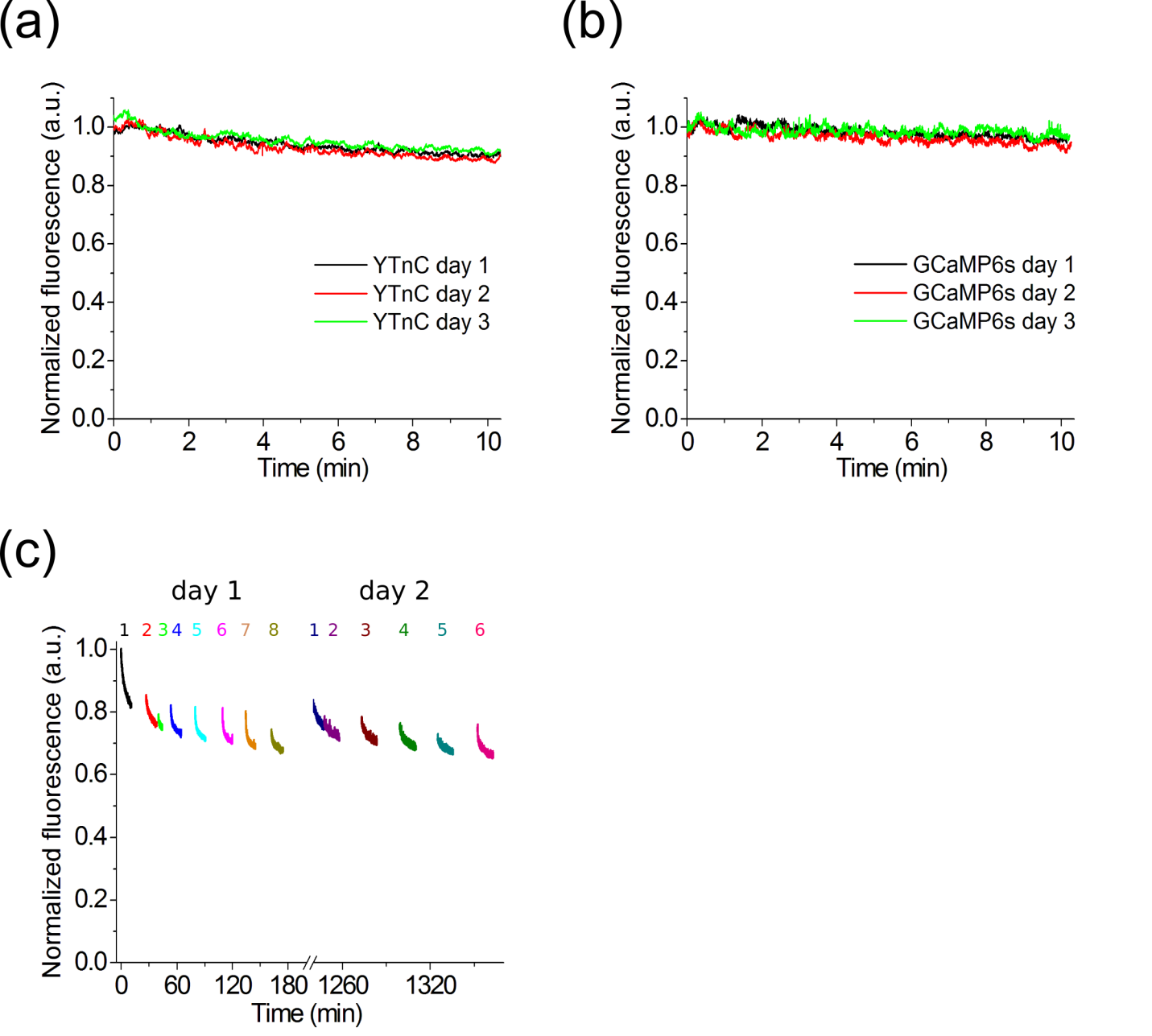


**Supplementary Figure 12. Comparison of the YTnC and GCaMP6s indicators photostability during single (a, b) and multiple 10-min imaging sessions (c) of mouse hippocampus using NVista miniscope.**


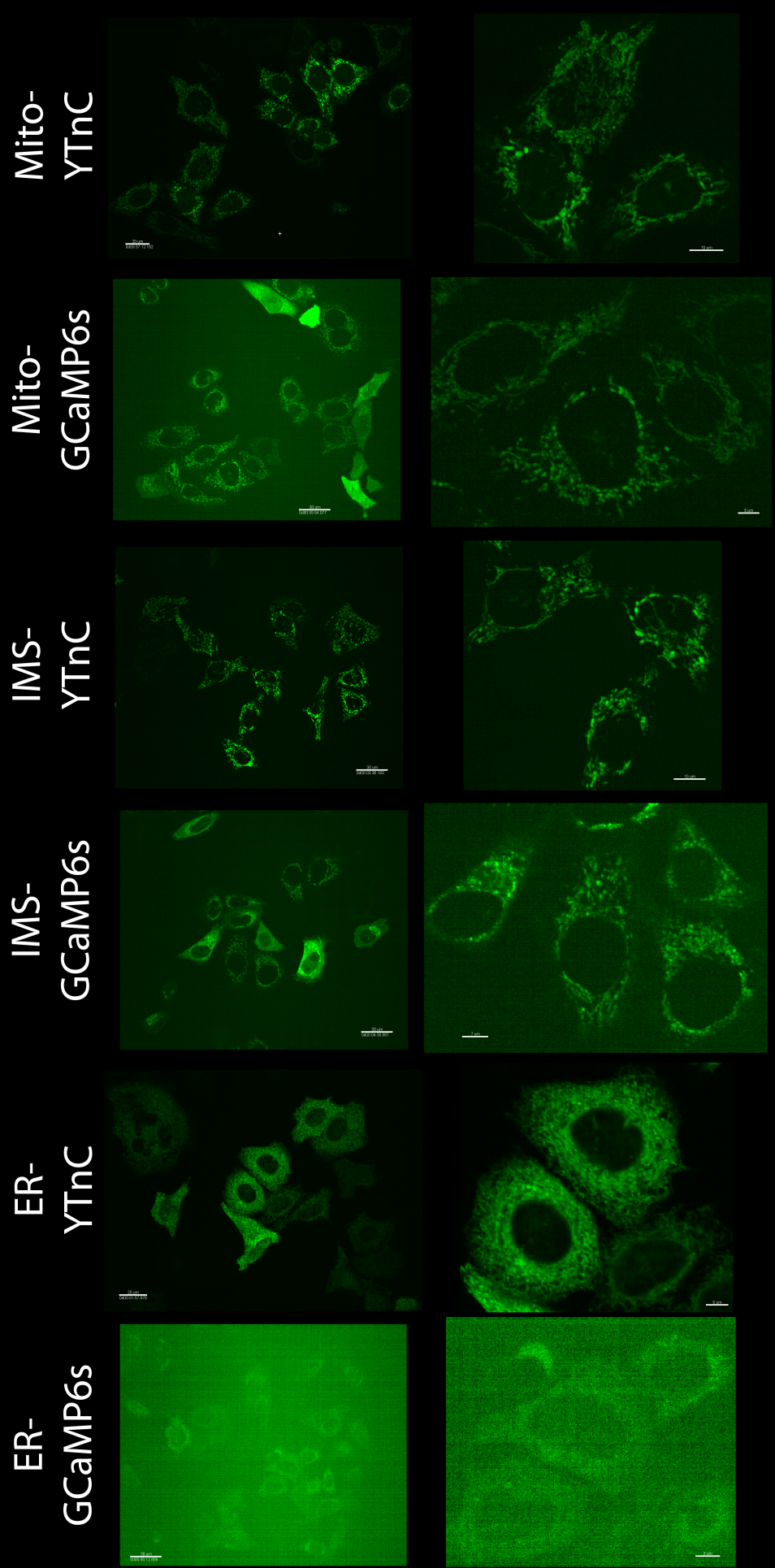


**Supplementary Figure 13. Localization of the YTnC and GCaMP6s calcium indicators targeted to the lumen and IMS of mitochondria and ER of the HeLa cells.** Confocal images of the cells transiently expressing listed fusions. The DNAs coding YTnC and GCaMP6s sensors were delivered to the HeLa cells using transient transfection with lipofectamine.


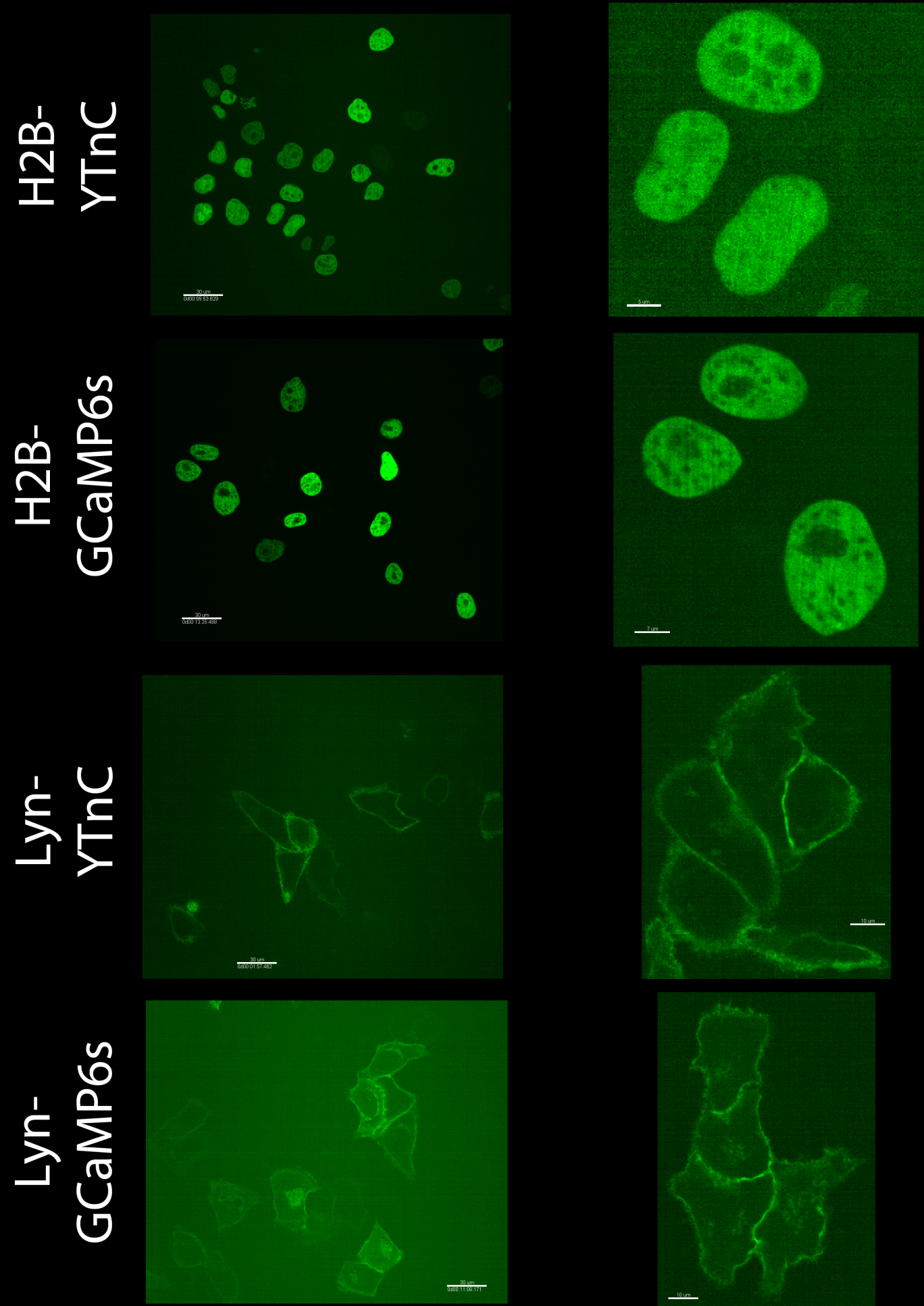


**Supplementary Figure 14. Localization of the YTnC and GCaMP6s calcium indicators targeted to the nucleus and plasma membrane of the HeLa cells.** Confocal images of the cells transiently expressing listed fusions. The DNAs coding YTnC and GCaMP6s sensors were delivered to the HeLa cells using transient transfection with lipofectamine.

**
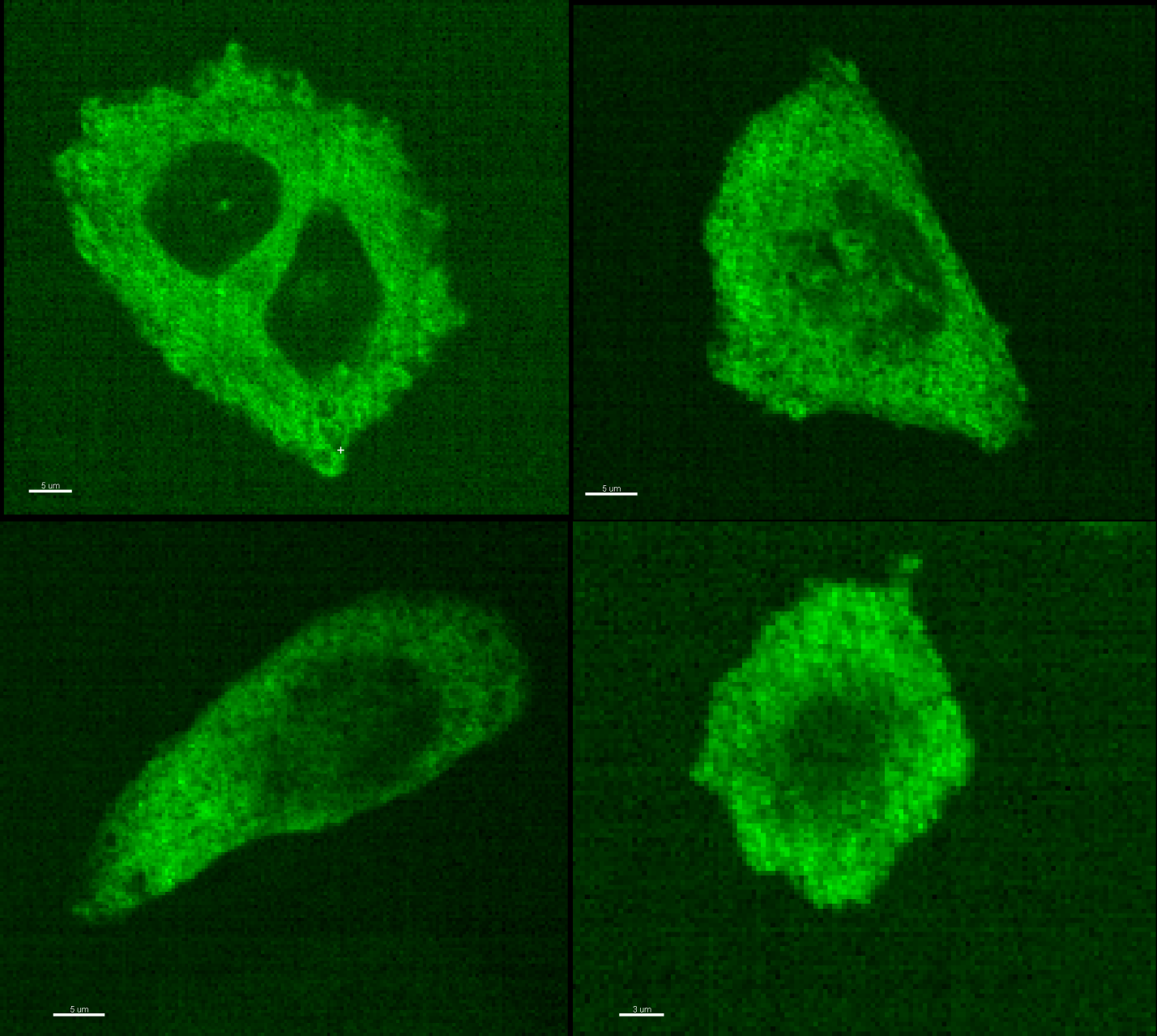
**

**Supplementary Figure 15. Localization of the GCaMP6s calcium indicator targeted to the ER of the HeLa cells after incubation at room temperature.** Confocal images of the cells transiently expressing ER-GCaMP6s fusion after incubation at r.t. for about 2-3 hours. The DNAs coding GCaMP6s sensor was delivered to the HeLa cells using transient transfection with lipofectamine.

**
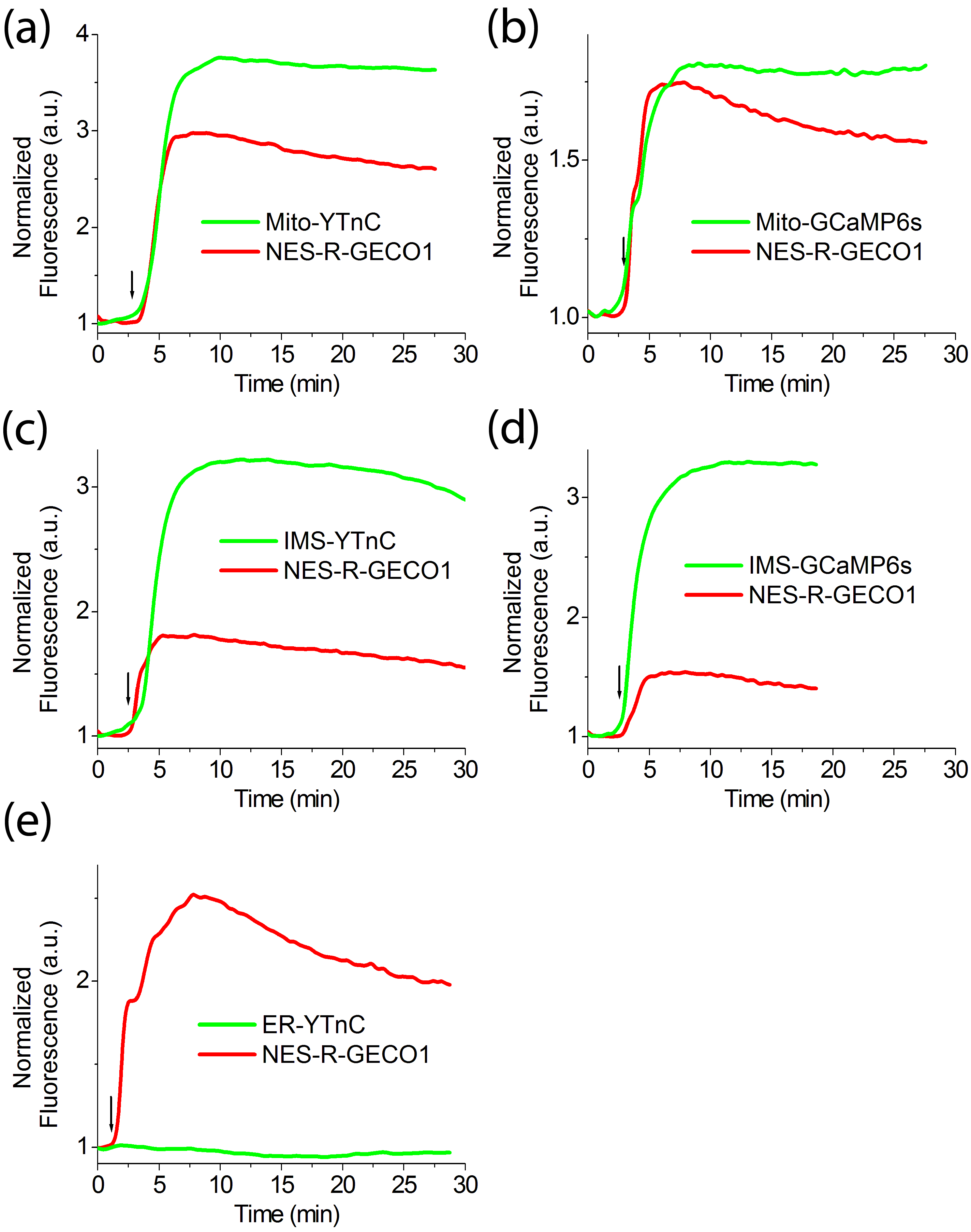
**

**Supplementary Figure 16. Comparison of the YTnC and GCaMP6s calcium indicators response in the lumen and IMS of mitochondria and ER of the HeLa cells.** Examples of time dependences were extracted from the series of confocal images of the cells transiently co-expressing listed fusions with NES-RGECO1. Addition of thapsigargin (10 µM final concentration) to DMEM supplemented with 20mM HEPES, pH 7.40, 10% FBS, Glutamine, 50 U/ml penicillin, and 50 U/ml streptomycin is shown with arrow. The DNAs coding YTnC and GCaMP6s sensors were mixed with DNA coding NES-R-GECO1 (3:1) and delivered to the HeLa cells using transient transfection with lipofectamine.


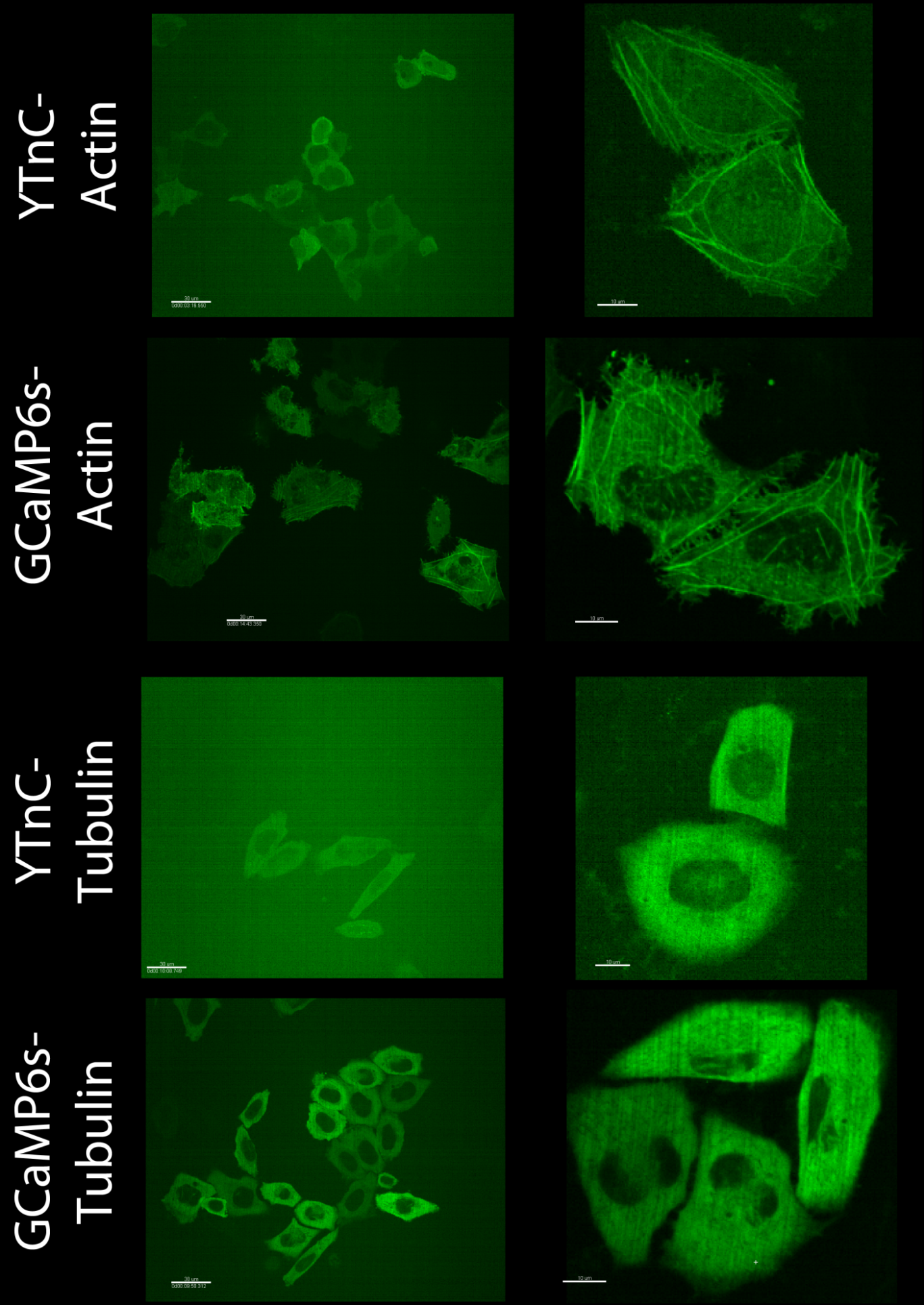


**Supplementary Figure 17. Localization of the YTnC and GCaMP6s calcium indicators targeted to the β-actin and α-tubulin microtubules of the HeLa cells.** Confocal images of the cells transiently expressing listed fusions. The DNAs coding YTnC and GCaMP6s sensors were delivered to the HeLa cells using transient transfection with lipofectamine.


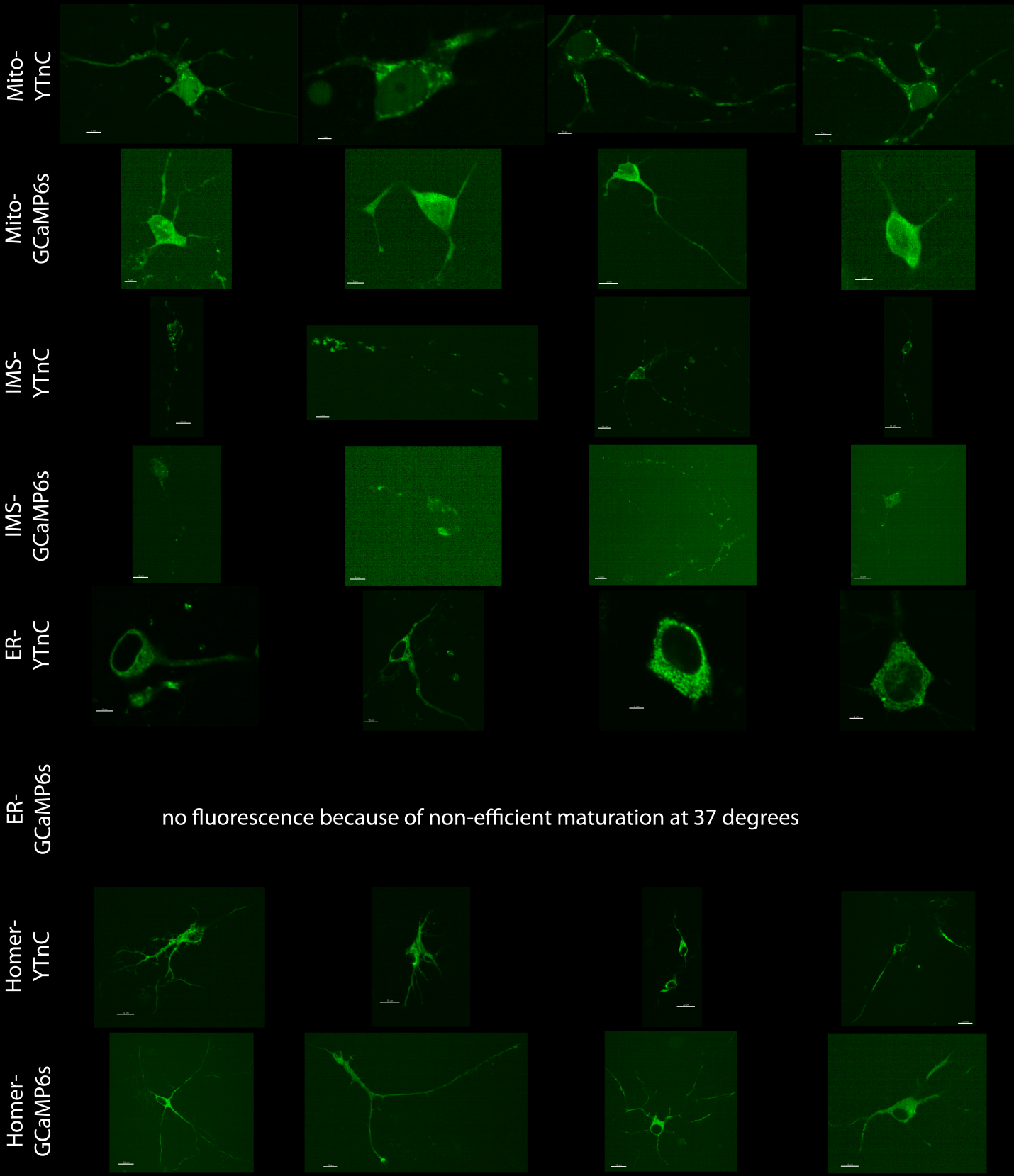


**Supplementary Figure 18. Localization of the YTnC and GCaMP6s calcium indicators targeted through their N-terminus to the different compartments of the neuronal cells.** Confocal images of the cells transiently expressing listed N-terminal fusions. The DNAs coding YTnC and GCaMP6s sensors were delivered to the neuronal cells using transient transfection with calcium-phosphate.


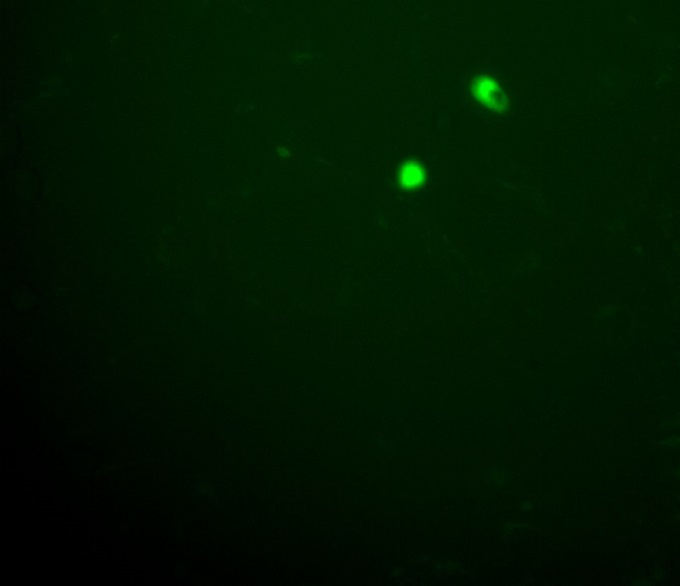

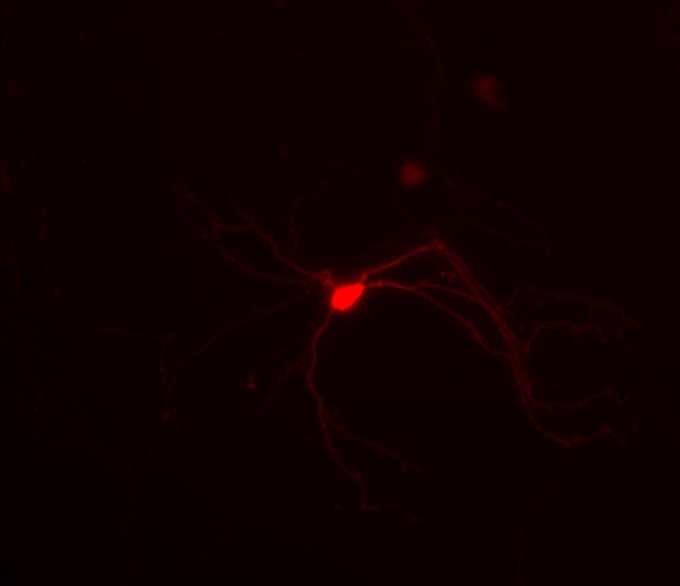

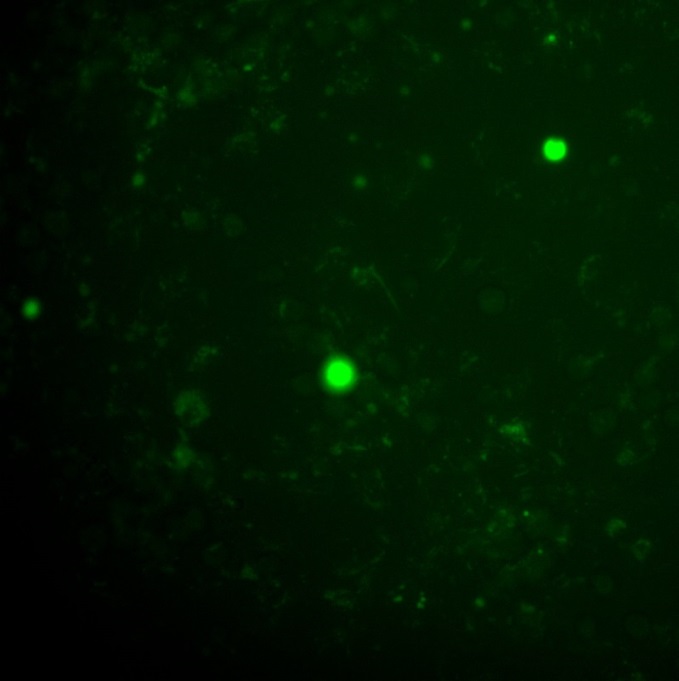

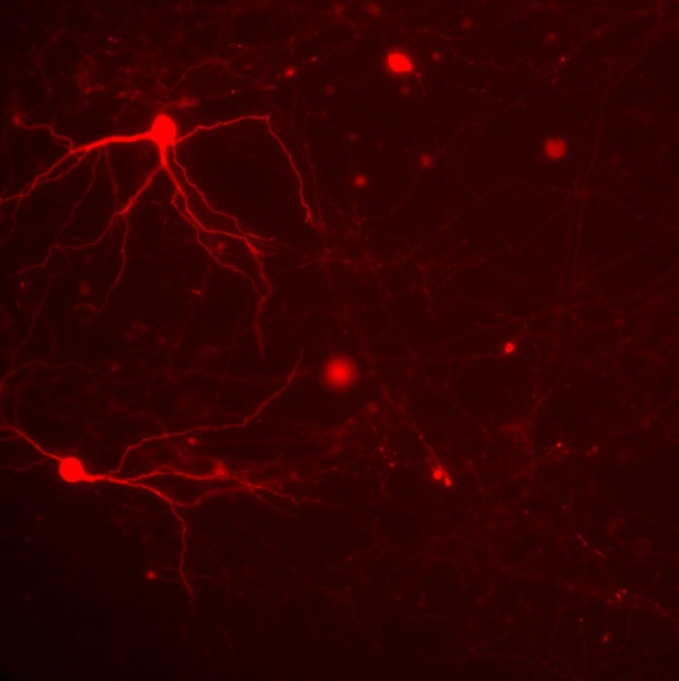


Green channel

Near-infrared channel

PSD95.FingR-GCaMP6s

PSD95.FingR-YTnC

iRFP682

iRFP682

**Supplementary Figure 19. Localization of the YTnC and GCaMP6s calcium indicators targeted through their N-terminal fusion with PSD95.FingR intrabody to the spines of the neuronal cells.** Representative fluorescence images of primary hippocampal neuron culture co-transfected with the DNA encoding PSD95.FingR-GCaMP6s + iRFP682 and PSD95.FingR-YTnC + iRFP682 delivered using transient transfection with calcium-phosphate. Scale bars, 50 µm.

**
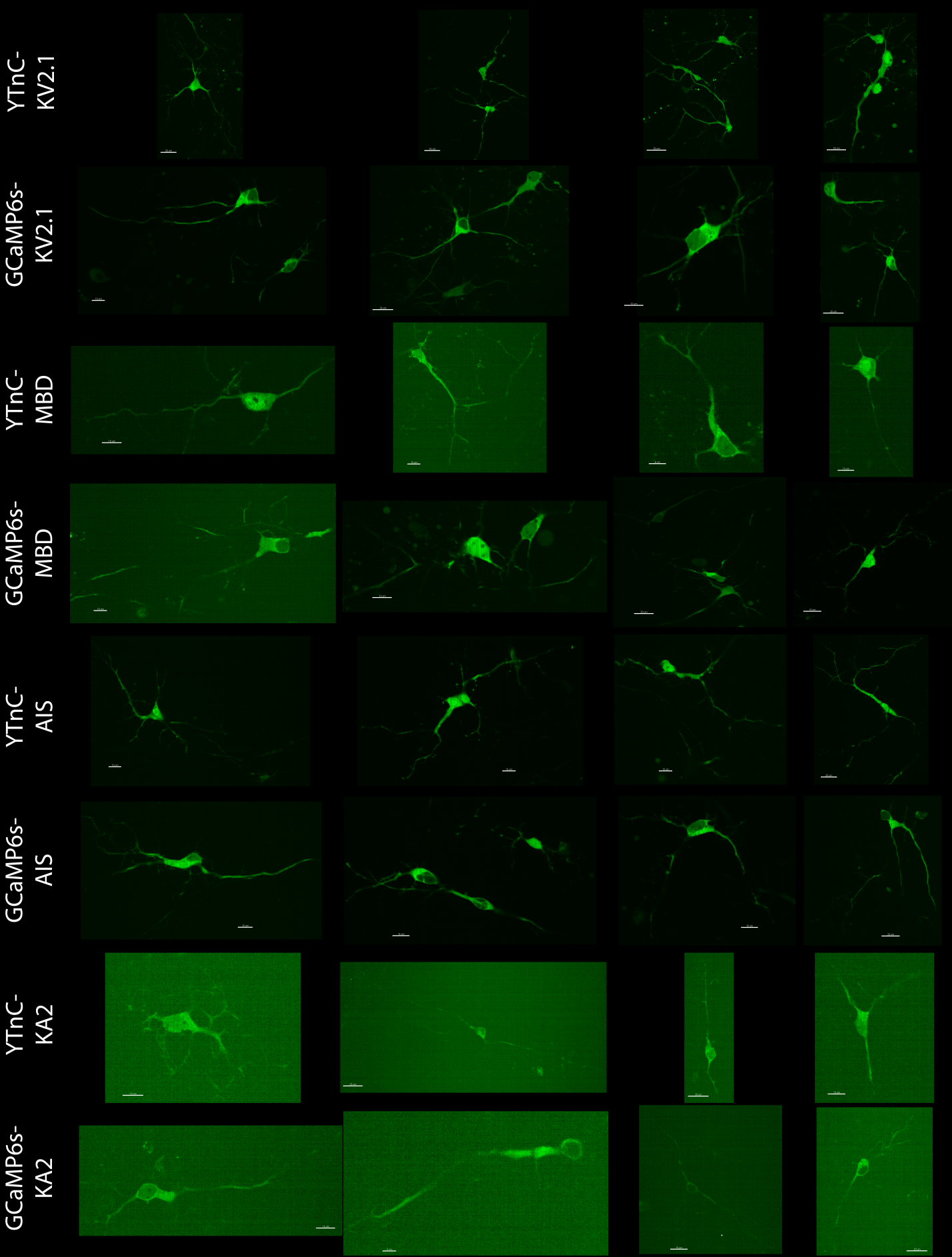
**

**Supplementary Figure 20. Localization of the YTnC and GCaMP6s calcium indicators targeted through their C-terminus to the different compartments of the neuronal cells.** Confocal images of the cells transiently expressing listed C-terminal fusions. The DNAs coding YTnC and GCaMP6s sensors were delivered in the neuronal cells using transient transfection with calcium-phosphate.


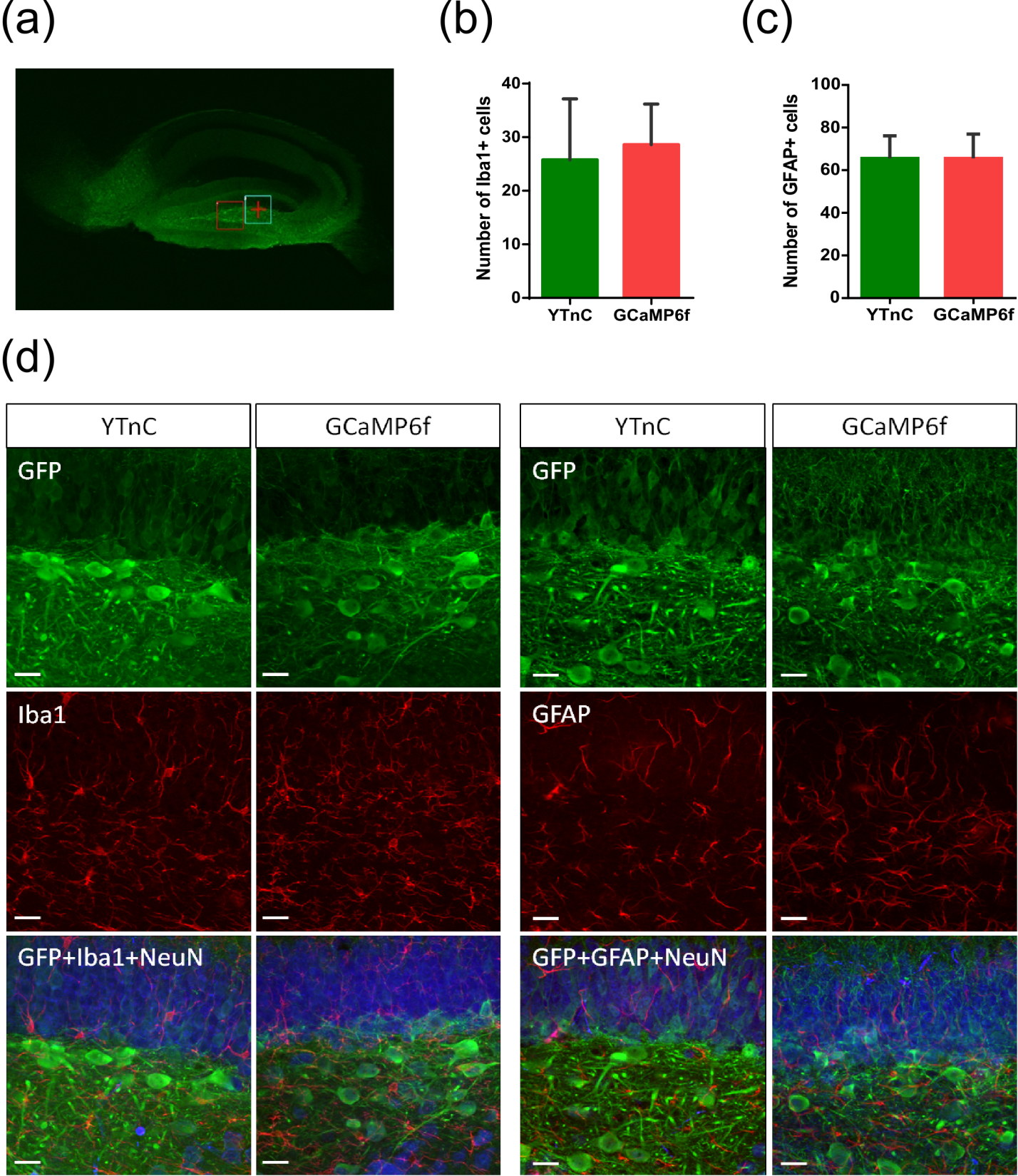


**Supplementary Figure 21. Comparison of the YTnC and GCaMP6f indicators cytotoxicity in dentate gyrus using immunohistochemistry to Iba1 and GFAP. (a)** Example of confocal image showing hippocampus with YTnC expression in dentate gyrus; two frames are shown where cell counting was performed. **(b, c)** Both YTnC and GCaMP6f expressing dentate gyri showed similar number of astrocytes and microglial cells estimated by the staining with GFAP and Iba1, respectively. Error bar corresponds to SD. **(d)** Representative images of Iba1, GFAP and NeuN expression in dentate gyri containing either YTnC or GCaMP6f indicators. Scalebar, 20 µm.


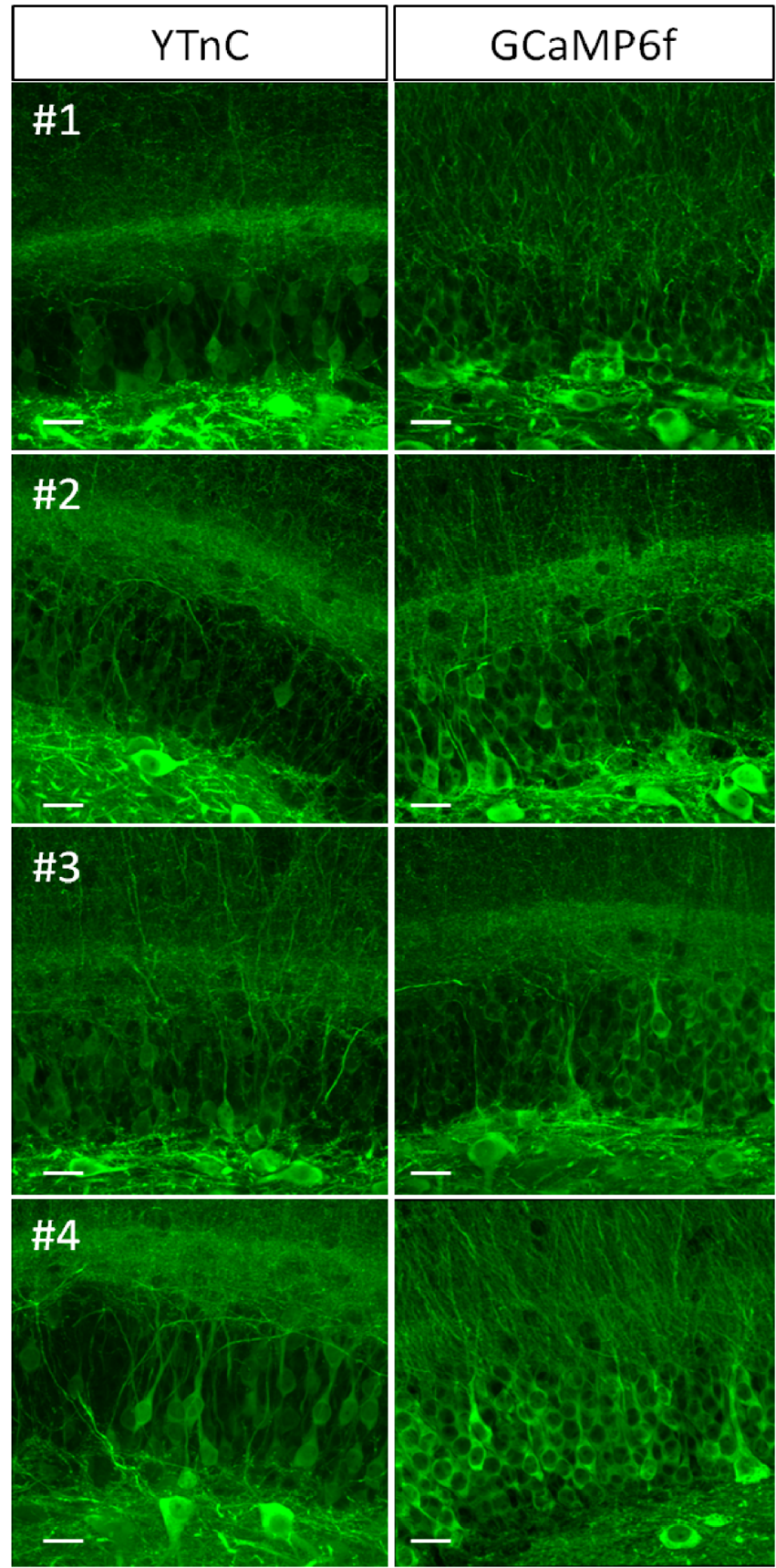


**Supplementary Figure 22. Comparison of the YTnC and GCaMP6f indicators distribution in the cell bodies, axons and spines in dentate gyrus of hippocampus across 4 mice.** YTnC and GCaMP6f indicators were stained immunohistochemically with antibodies against GFP. Scalebar, 20 µm


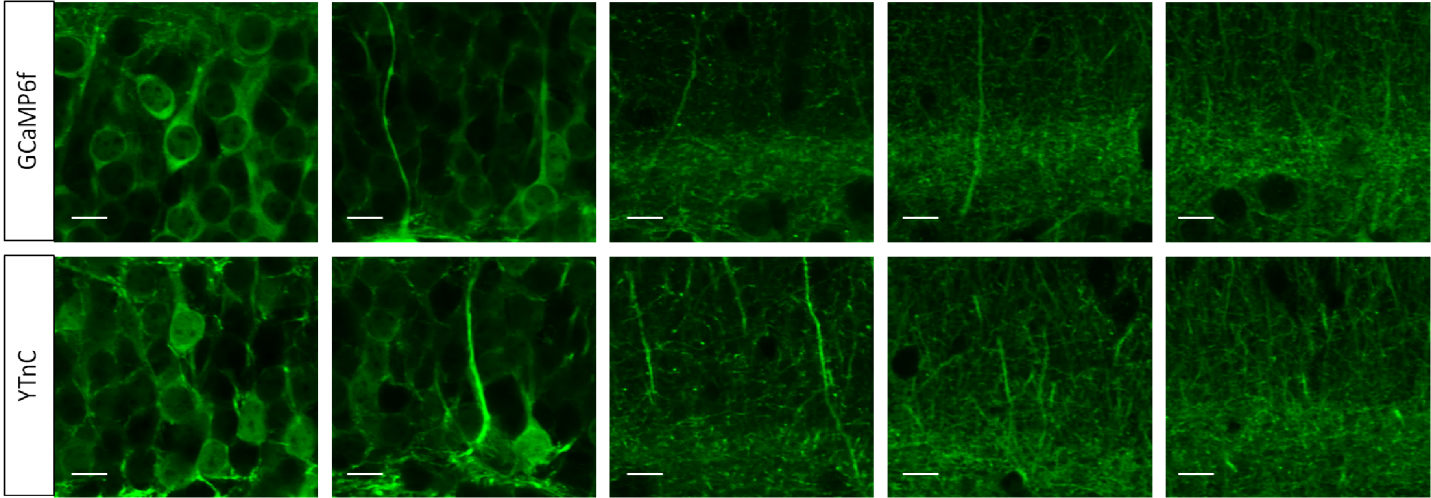


**Supplementary Figure 23. Zoomed comparison of the YTnC and GCaMP6f indicators distribution in the cell bodies, axons and spines in dentate gyrus of hippocampus across 1 mouse.** YTnC and GCaMP6f indicators were stained immunohistochemically with antibodies against GFP. Representative images from granular and molecular layers of dentate gyrus are shown. Scalebar, 10 µm


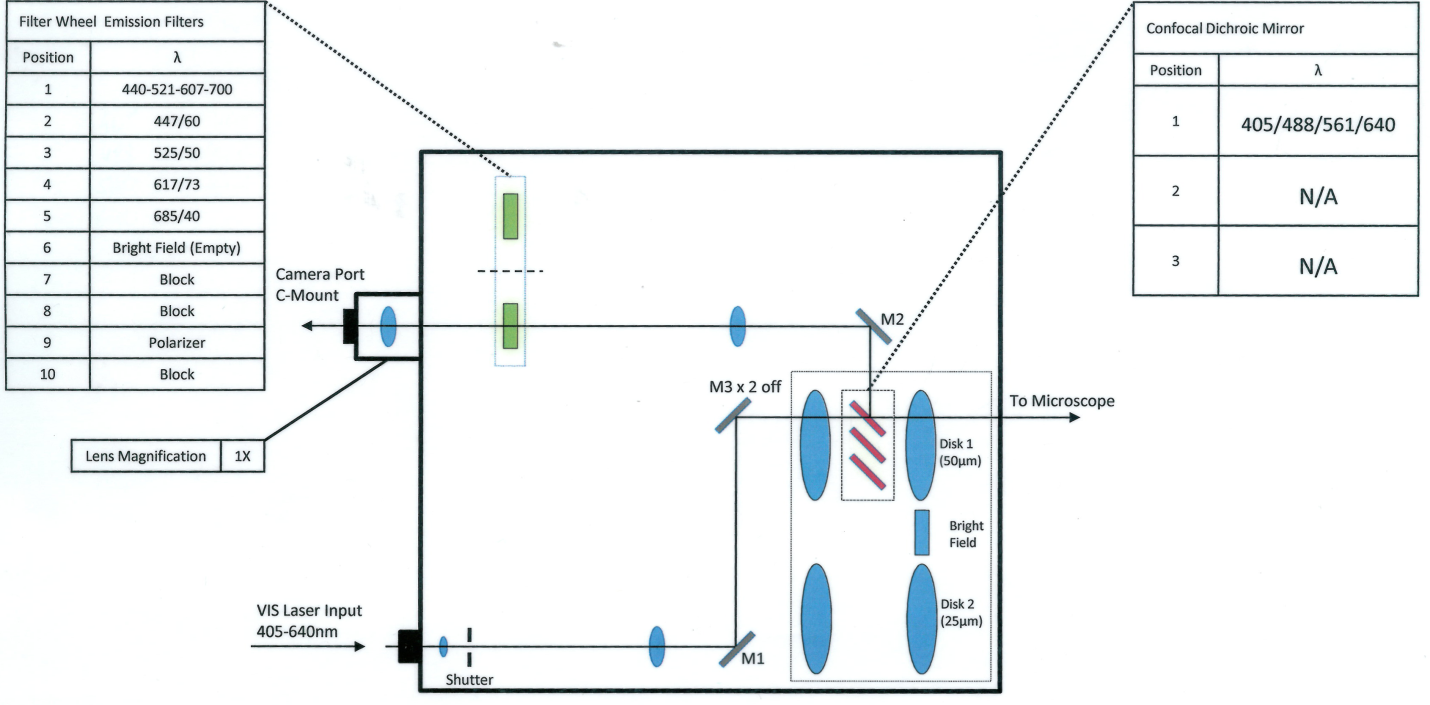


**Supplementary Figure 24. Optical configuration of spinning-disk module CSU-W1 from the Andor confocal imaging system.**

**
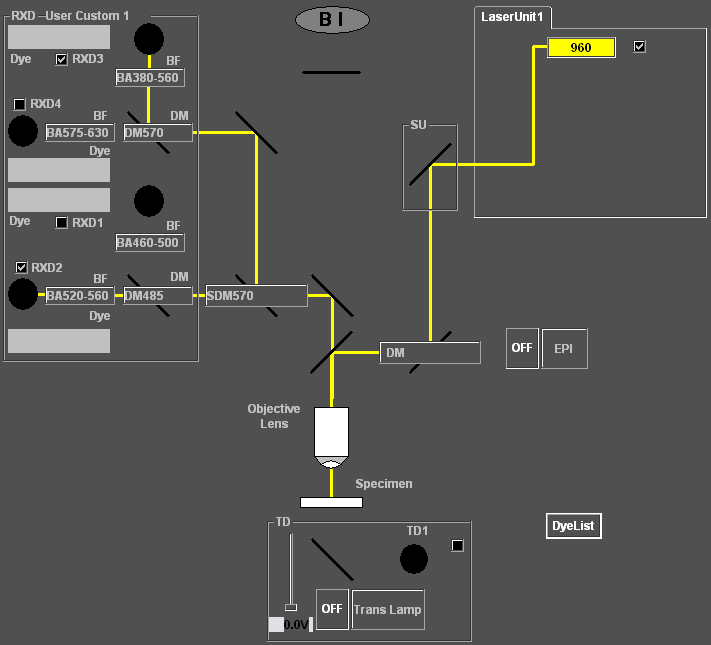
**

**Supplementary Figure 25. Optical configuration of the two-photon imaging system.** RXD2 and RXD3 detectors were used for the registration of green fluorescence and grating-specific signals, respectively.


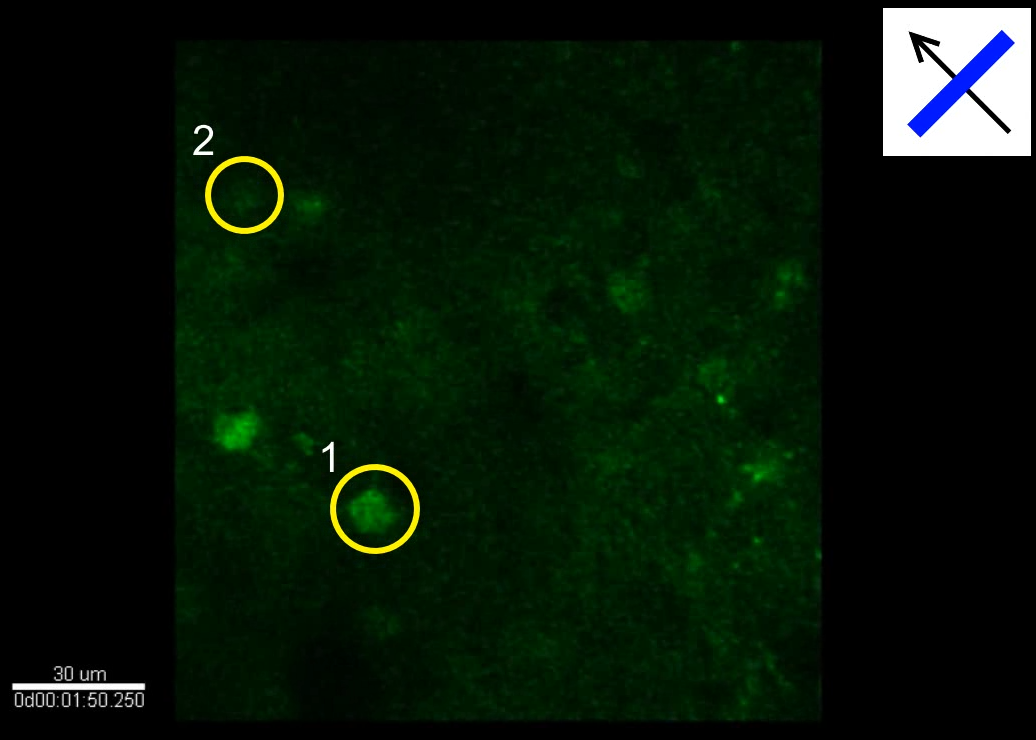


**Supplementary Video 1. *In vivo* visual stimuli evoked neuronal Ca^2+^ activity in the mouse cortex as visualized with the YTnC calcium indicator and two-photon microscopy.** Single frame for the time of 1m50s250ms is shown. Neurons specifically activated by the moving grating visual stimulus (shown as blue; the direction of grating is shown with dark arrow) are selected with circles. This field of view corresponds to the region 1 shown in the main text, Figure 5c.

**
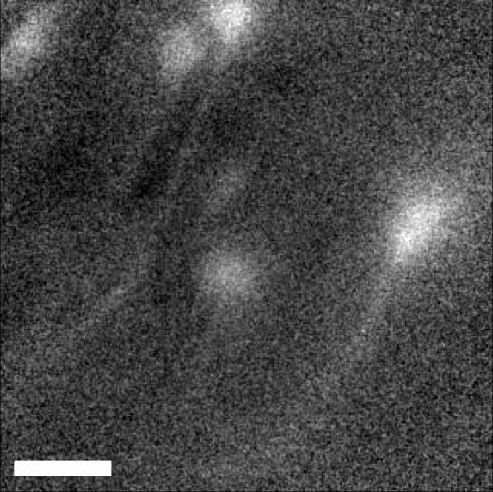
**

**Supplementary Video 2. *In vivo* neuronal calcium activity in hippocampus of anesthetized mouse visualized with the YTnC calcium indicator and nVista HD system**. A video of neuronal calcium activity in DG area of mouse hippocampus displayed as relative changes in fluorescence (ΔF/F) at 20 Hz frame rate. GLP 0561 lens probe was used. Frame is shown for the time of 16.4s. Scalebar, 50 µm.

**
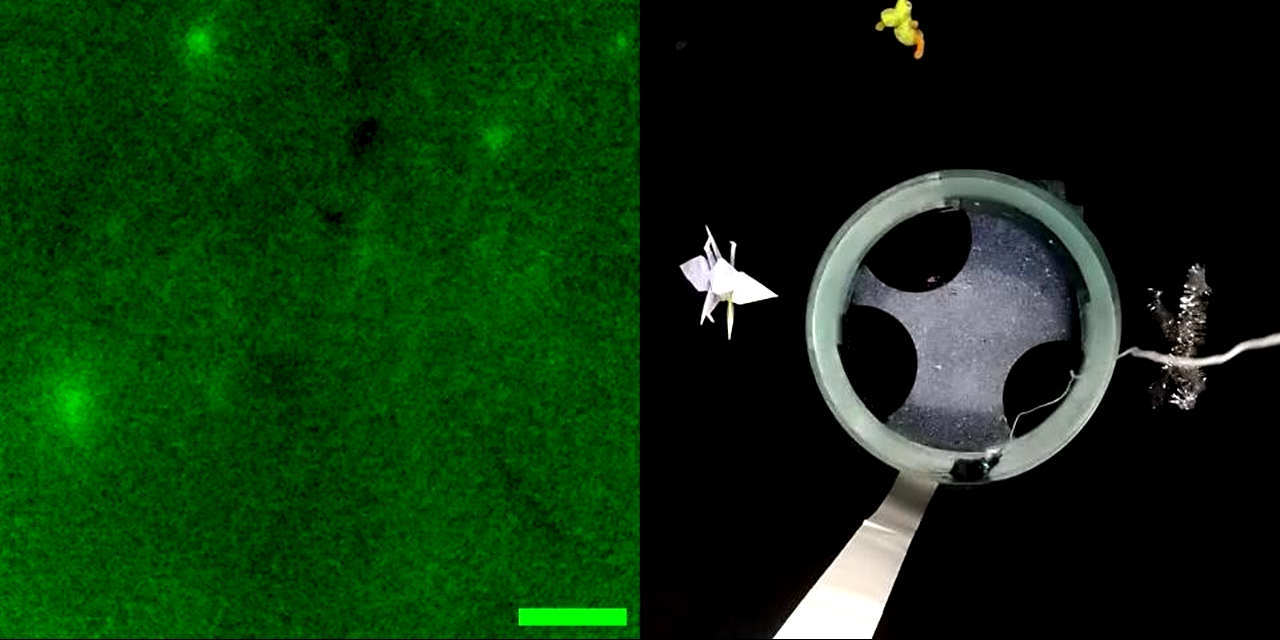
**

**Supplementary Video 3. *In vivo* neuronal calcium activity in hippocampus of mouse visualized with the YTnC calcium indicator and nVista HD system synchronized with mouse movement**. A video of neuronal calcium activity in CA1 area of mouse hippocampus displayed as relative changes in fluorescence (ΔF/F) at 20 Hz frame rate (left) synchronized with mouse movement in O-shaped track (right). GLP 1040 lens probe was used. Frame is shown for time of 19s. Scalebar, 100 µm.

**Supplementary Table 1.** **Characteristics of calcium responses to external field stimulation of neurons expressing the YTnC and GCaMP6s indicators in dissociated neuronal culture.**

| **Construct** | **ΔF/F_0_** | | **Normalized ΔF/F_0_^c^** | **N cells** |
| --- | --- | --- | --- | --- |
|  | **Green indicator** | **R-GECO1** |  |  |
| **NES-R-GECO1-P2A-NES-YTnC ^a^** | 0.21±0.03 | 1.07±0.57 | 0.25±0.16 | 3 |
| **NES-R-GECO1-P2A-NES-GCaMP6s ^a^** | 0.10±0.05 | 0.88±0.72 | 0.13±0.05 | 2 |
| **GCaMP6s and NES-R-GECO1 ^b^** | 1.51±0.94 | 1.13±0.66 | 1.57±0.87 | 14 |

^a^ Neurons expressing NES-R-GECO1-P2A-NES-YTnC or NES-R-GECO1-P2A-NES-GCaMP6s were imaged on DIV 8-9 using confocal microscope. Neuronal cultures were transfected on DIV 4 using calcium-phosphate approach. Errors correspond to standard deviations. Data were averaged across 2-14 cells. In the case of NES-R-GECO1-P2A-NES-GCaMP6s construct many neurons were red fluorescent only and did not show any green fluorescence; however HeLa cells transfected with the same plasmid appeared bright green and bright red upon addition of ionomycin (data not shown).

^b^ Neurons co-expressing the GCaMP6s green indicator and NES-R-GECO1 were imaged on DIV 20 using confocal microscope. Neuronal cultures were transduced on DIV 4 with the mixture of rAAVs viral particles carrying GCaMP6s and NES-R-GECO1. Errors correspond to standard deviations.

^c^ For calculation of normalized ΔF/F values, the maximal ΔF/F response of green indicator was normalized to the maximal ΔF/F response of red R-GECO1 indicator co-expressing in the same cell.

Electrical pulses of +-30V (460 us negative phase, 470 us interphase and 470 us positive phase) 87 Hz were generated using custom made stimulator (Supplementary Fig.15) and delivered to the neuronal culture via a pair of iridium electrodes (5mm gap). According to ΔF/F response of R-GECO1 (assuming that its ΔF/F values of ~0.14 corresponds to 1 AP) the stimulation induced ~3-12 APs.

**Supplementary Table 2.** **Characteristics of calcium responses to intracellular stimulation with 10 APs in neurons expressing the YTnC and GCaMP6s indicators in dissociated neuronal culture.**

| **Indicators** | **ΔF/F_0_** | **SNR** | **Rise time t_1/2_, ms** | **Decay time t_1/2_, s** |
| --- | --- | --- | --- | --- |
| **GCaMP6s (n=7)** | 0.74±0.08 | 310±50 | 304±11 | 9.7±1.2 |
| **YTnC (n=9)** | 0.28±0.03 | 108±20 | 192±6 | 5.2±0.7 |

Altogether 9 cells in 4 wells for YTnC and 7 cells in 3 wells for GCaMP6s were recorded.

**Supplementary Table 3. Characteristics of calcium responses in neurons expressing YTnC, GCaMP6f and GCaMP6s indicators in hippocampus of freely moving mice registered with nVista HD.**

| **Properties** | | **Indicators** | | |
| --- | --- | --- | --- | --- |
|  |  | **YTnC** | **GCaMP6f** | **GCaMP6s** |
| **Number of cells** | **assigned** | 562 | 306 | 1617 |
|  | **active ^a^** | 168 | 150 | 934 |
| **Average activity frequency, spikes/s** | | 0.0038 | 0.0051 | 0.0099 |
| **Rise half-time, ms ^b^** | | 680 | 390 | 550 |
| **Decay half-time, s ^b^** | | 2.4 | 1.2 | 2.0 |
| **Peak ΔF/F_0_ ^c^** | | 0.016±0.01 | 0.015±0.01 | 0.023±0.03 |
| **SNR ^d^** | | 9.2 | 10.2 | 6.1 |

^a^ Cell was considered as active if at least one spike exceeding 4MAD threshold was registered.

^b^ Rise and decay half-times were calculated as time intervals between the peak of the mean spike and half-peak at front and back slopes of the spike.

^c^ Peak ΔF/F_0_ was calculated as (F-F_0_)/F_0_, where F_0_ is the baseline fluorescence signal averaged over the whole period of imaging.

^d^ Signal-to-noise ratio (SNR) was quantified as peak ΔF/F_0_ response over median absolute deviation (MAD), calculated for each cell over the whole period of imaging.

^b-d^ For calculation of these characteristics we considered 476, 404 and 5610 single spikes for GCaMP6f (2 mice), YTnC (3 mice) and GCaMP6s (4 mice), respectively. Mean values ± standard error of mean are given.

**Supplementary Table 4.** **Sequences of the targeting motifs tested in this study.**

|  | **Motif abbreviation** | **Amino acid sequence (3'-5')** | **Suggested site of targeting in HeLa cells or neurons** |
| --- | --- | --- | --- |
| **N-terminal** | **NES** | MLQLPPLERLTLS | cytoplasm |
|  | **Mito** | MSVLTPLLLRGLTGSARRLPVPRAKIHSLGPA | lumen of mitochondria |
|  | **IMS** | MAALRSWVTRSVCSLFRYRQRFPVLANSKKRCFSELIKPWHKTVLTGFGMTLC | intermembrane space of mitochondria |
|  | **ER** | MSILLSPPSLLLLLAALVAPATS (+ KDEL on C-end of indicator) | endoplasmic reticulum |
|  | **H2B** | MPEPAKSAPAPKKGSKKAVTKAQKKGGKKRKRSRKESYSIYVYKVLKQVHPDTGISSKAMGIMNSFVNDIFERIAGEASRLAHYNKRSTITSREIQTAVRLLLPGELAKHAVSEGTKAITKYTSAK | nucleus |
|  | **Lyn** | MGCIKSKRKD | plasma membrane |
|  | **Homer** | MGEQPIFGTRAHVFQIDPNTKKNWVPTSKHAVTVSYFYDSTRNVYRIISLDGSKAIINSTITPNMTFTKTSQKFGQWADSRANTVYGLGFSSEHHLSKFAEKFQEFKEAARLAKEKSQEKMELTSTPSQESAGGDLQSPLTPESINGTDDERTPDVTQNSEPRAEPAQNALPFSHSAGDRTQGLSHASSAISKHWEAELATLKGNNAKLTAALLESTANVKQWKQQLAAYQEEAERLHKRVTELECVSSQANAVHSHKTELSQTVQELEETLKVKEEEIERLKQEIDNARELQEQRDSLTQKLQEVEIRNKDLEGQLSELEQRLEKSQSEQDAFRSNLKTLLEILDGKIFELTELRDNLAKLLECS | synapsis |
|  | **PSD95.FingR** | MLEVKEASPTSIQISWVLHLRHVRYYRITYGETGGNSPVQEFTVPGSKSTATISGLKPGVDYTITVYAVTIFSAYRSAWPPISINYRTGT |  |
| **C-terminal** | **Actin** | MDDDIAALVVDNGSGMCKAGFAGDDAPRAVFPSIVGRPRHQGVMVGMGQKDSYVGDEAQSKRGILTLKYPIEHGIVTNWDDMEKIWHHTFYNELRVAPEEHPVLLTEAPLNPKANREKMTQIMFETFNTPAMYVAIQAVLSLYASGRTTGIVMDSGDGVTHTVPIYEGYALPHAILRLDLAGRDLTDYLMKILTERGYSFTTTAEREIVRDIKEKLCYVALDFEQEMATAASSSSLEKSYELPDGQVITIGNERFRCPEALFQPSFLGMESCGIHETTFNSIMKCDVDIRKDLYANTVLSGGTTMYPGIADRMQKEITALAPSTMKIKIIAPPERKYSVWIGGSILASLSTFQQMWISKQEYDESGPSIVHRKCF | β-actin microtubules |
|  | **Tubulin** | VRECISIHVGQAGVQIGNACWELYCLEHGIQPDGQMPSDKTIGGGDDSFNTFFSETGAGKHVPRAVFVDLEPTVIDEVRTGTYRQLFHPEQLITGKEDAANNYARGHYTIGKEIIDLVLDRIRKLADQCTGLQGFLVFHSFGGGTGSGFTSLLMERLSVDYGKKSKLEFSIYPAPQVSTAVVEPYNSILTTHTTLEHSDCAFMVDNEAIYDICRRNLDIERPTYTNLNRLISQIVSSITASLRFDGALNVDLTEFQTNLVPYPRIHFPLATYAPVISAEKAYHEQLSVAEITNACFEPANQMVKCDPRHGKYMACCLLYRGDVVPKDVNAAIATIKTKRSIQFVDWCPTGFKVGINYQPPTVVPGGDLAKVQRAVCMLSNTTAIAEAWARLDHKFDLMYAKRAFVHWYVGEGMEEGEFSEAREDMAALEKDYEEVGVDSVEGEGEEEGEEY | α-tubulin microtubules |
|  | **KV2.1** | QSQPILNTKEMAPQSKPPEELEMSSMPSPVAPLPARTEGVIDMRSMSSIDSFISCATDFPEATRF | soma and proximal dendrites |
|  | **MBD** | RDQPLNSKKKKRLLSFRDVDFEEDSD | soma and dendrites |
|  | **AIS** | TVRVPIAVGESDFENLNTEDVSSESDP | axon initial segment |
|  | **KA2** | MPAELLLLLIVAFANPSCQVLSSLRMAAILDDQTVCGRGERLALALAREQINGIIEVPAKARVEVDIFELQRDSQYETTDTMCQILPKGVVSVLGPSSSPASASTVSHICGEKEIPHIKVGPEETPRLQYLRFASVSLYPSNEDVSLAVS | soma |

**Supplementary Table 5.** **Comparison of the YTnC and GCaMP6s indicators brightness and response to the addition of ionomycin in different compartments of HeLa cells.**

|  | **Indicator**  **in fusion** | **Relative brightness^a^** | **ΔF/F_0_** | **N**  **cells** |
| --- | --- | --- | --- | --- |
| **N-terminal fusions** | **Mito-YTnC** | 2.7±1.4 | 3.13±0.57 | 5 |
|  | **Mito-GCaMP6s** | 0.56±0.28 | 0.92±0.29 | 5 |
|  | **IMS-YTnC** | 2.9±1.1 | 2.26±0.30 | 5 |
|  | **IMS-GCaMP6s** | 1.06±0.77 | 0.92±0.22 | 6 |
|  | **ER-YTnC** | 2.2±1.1 | 0.30±0.20 | 5 |
|  | **ER-GCaMP6s** | 0.10±0.01 | no response | 5 |
|  | **H2B-YTnC** | 1.04±0.27 | 3.42±0.29 | 5 |
|  | **H2B-GCaMP6s** | 3.2±1.8 | 11.8±3.7 | 5 |
|  | **Lyn-YTnC** | 1.8±1.5 | 2.5±1.2 | 7 |
|  | **Lyn-GCaMP6s** | 0.43±1.45 | 3.7±1.2 | 6 |
| **C-terminal fusions** | **YTnC-Actin** | 0.20±0.11 | 1.49±0.35 | 5 |
|  | **GCaMP6s-Actin** | 1.21±0.52 | 13.91±0.35 | 5 |
|  | **YTnC-Tubulin** | 0.05±0.02 | 0.92±0.19 | 5 |
|  | **GCaMP6s-Tubulin** | 0.39±0.26 | 5.5±1.2 | 6 |
|  | **NES-YTnC** | 0.86±0.22 | 2.69±0.44 | 13 |
|  | **GCaMP6s** | 4.0±2.0 | 13.3±5.0 | 15 |

HeLa cells transiently co-expressing green indicators and NES-mCherry were imaged in DMEM medium supplemented with 20mM HEPES, pH 7.40, 10% FBS, Glutamine, 50 U/ml penicillin, and 50 U/ml streptomycin before and after addition of 2.5 µM ionomycin till indicator fluorescence reached plateau. Errors correspond to standard deviations. Data were averaged across 5-15 cells.

^a^ For estimation of relative brightness, the green fluorescence of calcium indicator was normalized to the red fluorescence of NES-mCherry RFP co-expressing in the same cell.

Note that we utilized DMEM medium instead of DPBS (Supplementary Fig. 6).

**Supplementary Table 6.** **Comparison of the YTnC and GCaMP6s indicators brightness and response to the addition of thapsigargin in different compartments of HeLa cells.**

| **Indicator**  **in fusion** | **Relative brightness^b^** | **ΔF/F_0_** | **Normalized**  **ΔF/F_0_^a^** | **N cells** |
| --- | --- | --- | --- | --- |
| **Mito-YTnC** | 12.2±4.4 | 3.37±0.46 | 1.8±0.9 | 12 |
| **Mito-GCaMP6s** | 2.9±1.8 | 1.47±0.65 | 0.83±0.25 | 11 |
| **IMS-YTnC** | 10.4±4.4 | 2.79±0.61 | 2.2±1.2 | 12 |
| **IMS-GCaMP6s** | 6.0±2.7 | 2.72±1.29 | 2.4±1.9 | 12 |
| **ER-YTnC** | 5.4±1.7 | no reaction^c^ | NA^d^ | 12 |

HeLa cells transiently co-expressing the respective green indicator and NES-R-GECO1 were imaged in DMEM medium supplemented with 20mM HEPES, pH 7.40, 10% FBS, Glutamine, 50 U/ml penicillin, and 50 U/ml streptomycin before and after addition of 2.5 µM ionomycin till indicator fluorescence passed maximum. Errors correspond to standard deviations. Data were averaged across 11-12 cells. Example of traces is shown on Supplementary Fig. 14.

^a^ For calculation of normalized ΔF/F values, the maximal ΔF/F response of green indicator was normalized to the maximal ΔF/F response of red R-GECO1 indicator co-expressing in the same cell.

^b^ For estimation of relative brightness, the green fluorescence of calcium indicators was normalized to the maximal red fluorescence of NES-R-GECO1 co-expressing in the same cell.

^c^ We did not see any reaction of the green ER-YTnC indicator upon addition of thapsigargin however R-GECO1 indicator co-expressing in the same cells showed ΔF/F response of 4.9±2.5.

^d^ NA, not applicable.

Note that we could not estimate response of ER-GCaMP6s in the same conditions because of its low brightness (Supplementary Fig. 10).

**Supplementary Table 7.** **Comparison of the YTnC and GCaMP6s indicators response during spontaneous activity of neuronal cultures.**

| **Indicator**  **in fusion** | **ΔF/F_0_** | **Normalized ΔF/F_0_^a^** | **N cells** |
| --- | --- | --- | --- |
| **Mito-YTnC** | 0.58±0.30 | 0.72±0.46 | 12 |
| **Mito-6s** | 1.10±0.67 | 0.86±0.46 | 10 |
| **IMS-YTnC** | 0.75±0.38 | 0.47±0.23 | 11 |
| **IMS-GCaMP6s** | 1.16±0.63 | 0.78±0.39 | 9 |
| **Homer-YTnC** | 1.22±0.80 | 0.64±0.27 | 9 |
| **Homer-GCaMP6s** | 1.14±0.52 | 0.97±0.61 | 9 |
| **YTnC-KV2.1** | 0.78±0.32 | 0.54±0.20 | 11 |
| **GCaMP6s-KV2.1** | 0.67±0.51 | 0.49±0.21 | 11 |
| **YTnC-MBD** | 1.13±0.55 | 0.63±0.29 | 13 |
| **GCaMP6s-MBD** | 1.34±0.72 | 0.70±0.28 | 12 |
| **YTnC-AIS** | 1.16±0.39 | 0.78±0.39 | 12 |
| **GCaMP6s-AIS** | 0.76±0.46 | 0.57±0.34 | 10 |
| **YTnC-KA2** | 0.94±0.47 | 0.82±0.55 | 9 |
| **GCaMP6s-KA2** | 0.83±0.67 | 0.91±0.76 | 16 |
| **NES-YTnC** | 0.47±0.26 | 0.82±0.11 | 7 |
| **NTnC** | 0.18±0.06 | 0.23±0.4^b^ | 8 |
| **GCaMP6s** | 1.28±1.83 | 0.91±1.15 | 32 |

Neurons co-expressing the respective green indicator and NES-R-GECO1 were imaged on DIV 13-17 using confocal microscope. Neuronal cultures were transduced on DIV 4 with the mixture of rAAVs viral particles carrying CAG-(indicator in fusion) and CAG-NES-RGECO1. Errors correspond to standard deviations. Data were averaged across 7-32 cells.

^a^ For calculation of normalized ΔF/F values, the maximal ΔF/F response of green indicator was normalized to the maximal ΔF/F response of red R-GECO1 indicator co-expressing in the same cell.

^b^ Data were averaged across 3 cells.

**Supplementary Table 8.** **List of primers.**

| **Primer** | **Primer sequence (5’-3’)** |
| --- | --- |
| **Fw-YFP-BglII** | gacAGATCTATGGTGAGCAAGGGCGAGGAG |
| **Rv-YFP-EcoRI** | GTCGAATTCttacttgtacagctcgtccatgcc |
| **YFP-144-TnC** | cacaagctggagtacaacNNSNNSNNSAGCGAAGAAGAGCTATC |
| **YFP-144-TnC-r** | GATAGCTCTTCTTCGCTSNNSNNSNNGTTGTACTCCAGCTTGTG |
| **TnC-146-YFP** | GATGGTGGAGAATGTCCAGNNSNNSaacagccacaacgtctatatc |
| **TnC-146-YFP-r** | GATATAGACGTTGTGGCTGTTSNNSNNCTGGACATTCTCCACCATC |
| **TnC-145-YFP** | GATGGTGGAGAATGTCCAGNNSNNStacaacagccacaacgtc |
| **TnC-145-YFP-r** | GACGTTGTGGCTGTTGTASNNSNNCTGGACATTCTCCACCATC |
| **YFP-147-TnC** | ggagtacaactacaacagcNNSNNSNNSAGCGAAGAAGAGCTATC |
| **YFP-147-TnC-r** | GATAGCTCTTCTTCGCTSNNSNNSNNGCTGTTGTAGTTGTACTCC |
| **TnC-149-YFP** | GATGGTGGAGAATGTCCAGNNSNNSaacgtctatatcatggcc |
| **TnC-149-YFP-r** | GGCCATGATATAGACGTTSNNSNNCTGGACATTCTCCACCATC |
| **BamHI-NES-mCher** | GGG GAT CCG ACA CCA TGC TTC AAC TTC CTC CTC TTG AAC GTC TTA CTC TTT CGA GAT CTG CTA GCC TCG AGA TGG TG |
| **mCher-BsrGI-r2** | ACTTGTACATTACTTATACAGCTCGTCCATGCCGCCGGTG |
| **Mito-Kpn I** | ataggatccggtaccgccaccATGTCCGTCCTGACGCC |
| **Mito-BamHI** | ataggatccaccggtgacaccATGTCCGTCCTGACGCC |
| **Mito-NheI-r** | GAGGCTAGCAGATCTTGCAGGCCCCAACGAATG |
| **YTnC-NheI** | cgagatctgctagcATGGTGAGCAAGGGCGAG |
| **GCaMP-NheI** | cgagatctgctagcATGGTCGACTCATCACGTC |
| **TnC-EcoRI-HindIII-r** | AGA AAG CTT GAA TTC TTA CTT ATA CAG CTC GTC CAT G |
| **GCaMP-EcoRI-HindIII-r** | GTC AAG CTT GAA TTC CTA CTT CGC TGT CAT CAT TTG |
| **TnC-EcoRI-HindIII-r** | GTC AAG CTT GAA TTC TTA CTT ATA CAG CTC GTC CAT G |
| **IMS-AgeI** | taaccggtgacaccAtggcggctctgagaagttgg |
| **IMS-BglII-r** | GCAGATCTtgatcctcctgatcCACACAGTGTCATGCCAAATCC |
| **ER-AgeI** | taaccggtgacaccATGAGCATCCTACTGTCG |
| **YTnC-KDEL-EcoRI-r** | cagGAATTCTCACAGCTCGTCCTTGTACAGCTCGTCCATG |
| **GCaMP-KDEL-EcoRI-r** | cagGAATTCTCACAGCTCGTCCTTCGCTGTCATCATTTG |
| **Lyn-YTnC-AgeI** | GCAaccggtaccAtgggctgcATCAAGtctAAGcgcAAAgatATGGTGAGCAAGGGCGAG |
| **Lyn-GCaMP-AgeI** | GCAaccggtaccAtgggctgcATCAAGtctAAGcgcAAAgatATGGTCGACTCATCACG |
| **Homer-AgeI** | GCAaccggtgacaccATGGGGGAACAACCTATC |
| **Homer-NheI-r** | GAGGCTAGCccctccactcccggagctagaaccgctggacccGCTGCATTCTAGTAGCTTG |
| **Neon-EcoRI-rv3** | TCG GAA TTC CTT ATA CAG CTC GTC CAT G |
| **YTnC-AgeI** | taaccggtgacaccATGGTGAGCAAGGGCGAG |
| **GCaMP-AgeI** | taaccggtgacaccATGGTCGACTCATCACGTC |
| **GCaMP-EcoRI-r2** | cagGAATTCCTTCGCTGTCATCATTTG |
| **KV2-EcoRI** | AGGAATTCgctagcagcggaggtactCAGTCCCAGCCCATCCTC |
| **KV2-HindIII-r** | gataagcttTTAGAATCTGGTGGCTTCAGG |
| **AIS-EcoRI** | AGGAATTCagcggaggtactACCGTGCGGGTGCCCATTGCTGTGGGCGAGTCTGATTTCGAGAACC |
| **AIS-HindIII-r** | gataagcttTTAGGGGTCTGACTCGCTGCTAACATCCTCTGTGTTGAGGTTCTCGAAATCAGACTC |
| **Actin-NheI** | aTCgctagcagcggaggtactATGGATGATGATATCGCC |
| **Actin-HindIII-r** | gataagcttCTAGAAGCATTTGCGGTGGAC |
| **Tub-NheI** | aTCgctagcagcggaggtactGTGCGTGAGTGCATCTCC |
| **Tub-HindIII-r** | gataagcttTTAGTATTCCTCTCCTTCTTC |
| **H2B-AgeI** | GCAaccggtgacaccATGCCAGAGCCAGCGAAG |
| **H2B-BglII-r** | TCAGATCTCTTAGCGCTGGTGTACTTG |
| **MBD-EcoRI** | AGGAATTCggaagcggtagcggtggatcaggctctAGAGACCAGCCCCTCAACAGCAAAAAGAAAAAGCGC |
| **MBD-HindIII-r** | gataagcttTTAGTCTGAGTCTTCCTCAAAGTCCACATCTCGGAAGGAGAGCAGGCGCTTTTTCTTTTTGCTG |
| **KA2-EcoRI2** | AGGAATTCGGAGGTTCAGGTGGAACC |
| **KA2-HindIII-r2** | gataagcttTTAGCTGACTGCCAGTGACAC |

**Supplementary Methods**

**Libraries screening**

Screening of bacterial libraries was sequentially performed on Petri dishes, bacterial suspensions in a 96-well plate format, and purified proteins.

Primary screening of approximately 10,000 – 20,000 colonies of bacterial library expressing calcium sensors variants was performed on Petri dishes under fluorescent stereomicroscope Leica M205FA (Leica, Germany) equipped with DFC310FX camera (Leica Microsystems, Germany) and mercury metal halide light source EL6000 (Leica Microsystems, Germany). Expression of the sensors in the colonies on Petri dishes was induced with 0.0002% arabinose for 16 h at 37°C and 24 h at room temperature (r.t.). Reaction of the sensors with calcium ions was further monitored under the fluorescent stereomicroscope Leica. Green fluorescence was registered by 480/40BP excitation (75 µW/cm^2^ on the sample) and 540/40BP emission filters, respectively. Fluorescence images of Petri dishes with bacterial colonies were taken with the same exposure time before and after spraying the plates with 100 mM EDTA, 20 mM Tris-HCl at pH 8.0. Images obtained were analyzed using ImageJ software and colonies having the highest brightness and contrast were picked up for further analysis.

Next, approximately 60-96 mutants selected through colonies analysis were analyzed on bacterial suspensions using 96-well Modulus^TM^ II Microplate Reader (Turner Biosystems, USA). For this purpose, the best clones picked up from Petri dishes were grown in 200 uL aliquots of LB medium containing 100 μg/ml ampicillin, 0.0002% arabinose, and 100 μM CaCl_2_ for 12-16 h at 220 rpm and 37°C and for 24 h at r.t. Bacterial suspensions containing 180 µl of 100 mM NaOAc pH 7.4 buffer supplemented with 100 mM CaCl_2_ and 20 µl of bacterial culture were aliquoted onto 96-well plates. These suspensions were incubated at r.t. for 1 h with measurement of the fluorescence signal. Afterwards, EDTA solution was added until a final concentration of 0.4 mM was reached, followed by fluorescence registration for 10 min. Next, a solution of CaCl_2_ was added until a final concentration of 5 mM, followed by fluorescence recording for 10 min. Data collected were analyzed using the Origin 6.0 software, as plots of dependence of fluorescence vs time. Clones having the highest brightness and contrast in response to addition of CaCl_2_ and EDTA were selected. During the last two rounds, the analysis of bacterial suspensions was replaced with analysis of protein extracts in BPer reagent.

The best clones found in 96-well format were subsequently grown for protein purification in LB supplemented with 0.0002% arabinose, 100 μg/ml ampicillin and 100 µM CaCl_2_ overnight at 37°C, 220 rpm. The cultures were centrifuged at 1,640 g for 15 min. The cell pellets were resuspended in B-Per solution (Thermo Scientific, USA) containing 1 mg/mL lysosyme, and 20 u/mL DNAse I (Invitrogen, USA). The recombinant proteins were purified using Ni-NTA resin (Qiagen, USA). Purified proteins were characterized for brightness (product of quantum yield and extinction coefficient) and contrast as described below. Clones exhibited increased brightness and contrast compared to clones from previous round of mutagenesis were subjected to the next round of random mutagenesis.

**Protein purification and characterization**

The bacterial cultures carrying pBAD/HisB-TorA-indicator plasmid were grown in LB medium supplemented with 0.002% arabinose and 100 μg/ml ampicillin overnight at 37°C and 220 rpm. The cultures were then centrifuged at 4648 g for 10 min, and the cell pellets were re-suspended in PBS at pH 7.4 with 300 mM NaCl and lysed by sonication on ice. The recombinant proteins were purified using Ni-NTA resin (Qiagen, USA), followed by dialysis for 12-16 h against buffer A (30 mM HEPES, 100 mM KCl, pH 7.2) or buffer A supplemented with either 10 mM EDTA or 10 mM CaCl_2_. The absorbance values and excitation and emission spectra were measured with a CM2203 spectrofluorometer (Solar, Belarus).

Chromophore extinction coefficient for purified YTnC_apo_ in Ca^2+^-free state was measured in buffer A supplemented with 10 mM EDTA by alkaline denaturation with 1 M NaOH and using extinction coefficient for GFP-like chromophore equal to 44,000 M^-1^ cm^-1^ in 1 M NaOH^[1](#_ENREF_1)^. Chromophore extinction coefficient for purified YTnC_sat_ in Ca^2+^-saturated state was calculated in buffer A supplemented with 10 mM CaCl_2_ by the comparison of its absorption spectrum with that of YTnC_apo_ having the same absorbance at 280 nm.

For quantum yield determination, the integrated fluorescence values of purified YTnC in the Ca^2+^-free and Ca^2+^ saturated states were measured in buffer A supplemented with either 10 mM EDTA or 10 mM CaCl_2_, respectively, as previously reported[^2^](#_ENREF_2).

For equilibrium K_d_ determination, the two stock buffers 30 mM HEPES, 100 mM KCl, pH 7.2 containing 10 mM EGTA (zero free Ca^2+^) or 10 mM Ca-EGTA (39 µM free Ca^2+^) were mixed in various ratios to give solutions with different free Ca^2+^ concentrations, as described previously[^3^](#_ENREF_3). The purified YTnC protein in buffer A was added (1:100, till final concentration of 2µg/ml) to prepared solutions with different free Ca^2+^ concentrations. After 20 min of equilibration at r.t. its green fluorescence was measured on Modulus^TM^ II Microplate Reader (TurnerBiosystems, USA). Three replicates were averaged for analysis. The K_d_ and Hill coefficient values were determined from the fitting of experimental data by the Hill’s equation. Titration of YTnC to Ca^2+^ ions in the presence of Mg^2+^ was performed in the same manner except two stock buffers 30 mM HEPES, 100 mM KCl, 1 mM MgCl_2_, pH 7.2 containing 10 mM EGTA (zero free Ca^2+^) or 10 mM Ca-EGTA (39 µM free Ca^2+^) were mixed.

For determination of the pH dependence, YTnC, GCaMP6f and NTnC proteins were dialyzed in buffer A (30 mM HEPES, 100 mM KCl, pH 7.2) supplemented with either 10 mM EDTA or 10 mM CaCl_2_. Next, they were diluted 1:100 into a series of pH adjusted buffers (30 mM citric acid, 30 mM borax, or 30 mM NaCl) with pH values ranging from 9 to 3 in 0.5 pH units interval in a 96-well black clear bottom plate (Thermo Scientific, USA), as described in the original paper[^3^](#_ENREF_3). Fluorescence was measured using a Modulus^TM^ II Microplate Reader (TurnerBiosystems, USA).

Photobleaching experiments were performed with suspensions of purified proteins in mineral oil, as previously described[^2^](#_ENREF_2). Briefly, the kinetics of photobleaching was measured using purified proteins dialyzed in buffer A (30 mM HEPES, 100 mM KCl, pH 7.2) supplemented with either 10 mM EDTA or 10 mM CaCl_2_, at a 1 mg/ml concentration, in aqueous microdroplets in mineral oil using Zeiss Axio Imager Z2 microscope (Zeiss, Germany) equipped with a 120 W mercury short-arc lamp (LEJ, Germany), a 63× 1.4 NA oil immersion objective lens (PlanApo, Zeiss, Germany), a 470/40BP excitation filter, a FT 495 beam splitter, and 525/50BP emission filters. Light power density (7.3 mW/cm^2^) was measured at a rear focal plane of the objective lens using PM100D power meter (ThorLabs, Germany) equipped with S120VS sensor (ThorLabs, Germany). The times to photobleach from 1000 down to 500 emitted photons per second were calculated according to standard procedures[^4^](#_ENREF_4). In brief, the averaged raw data were corrected for **a spectral output of the lamp, transmission profiles of the excitation filter and dichroic mirror, and absorbance spectra of the respective green fluorescent proteins** and their quantum yields. EGFP protein that has been characterized according to this procedure in previous work[^4^](#_ENREF_4) was used as a reference.

To study protein maturation, BW25113 bacteria transformed with the pBAD/HisB-TorA-YTnC, pBAD/HisB-TorA-NTnC or pBAD/HisB-EGFP plasmids were grown in 150 ml of LB medium supplemented with ampicillin at 37°C overnight. The next morning 0.2% arabinose was added to bacterial cells. Upon induction of protein expression, bacterial cultures were grown at 37°C in 50 ml tubes filled to the brim and tightly sealed to restrict oxygen supply. After 2 hours, the cultures were centrifuged in the same tightly closed tubes. After opening the tubes, the bacteria were sonicated in PBS buffer and the resulting proteins were purified using Ni-NTA resin within 10 min, with all procedures and buffers at or below 4°C. Protein maturation occurred in Ca^2+^-free or Ca^2+^-saturated buffers at 37°C. Green fluorescence signal of the proteins was monitored using a CM2203 spectrofluorometer (Solar, Belarus).

Size-exclusion chromatography was performed with a SuperdexTM 75 10/300 GL column using GE AKTA Explorer (Amersham Pharmacia, UK) FPLC System.

**Stopped-flow fluorimetry**

Ca^2+^-binding kinetics experiments were performed on a Chirascan Spectrofluorimeter (Applied Photophysics, UK) equipped with a stopped-flow module at 20°C. Fluorescence excitation was set to 493 nm, and fluorescence emission was collected using a 515 nm cut-off filter. Three replicates were averaged for analysis. Kinetic records were fitted to either a single or a double exponential using DataFit9 (Oakdale Engineering, USA).

To measure association kinetics, YTnC or control GCaMP6f (20 μg/ml) in buffer A supplemented with 1 mM EGTA was rapidly mixed (1:1) with buffer A supplemented with 10 mM EGTA and increasing Ca^2+^ concentrations. Exponential fitting of the fluorescence signal changes over time provided the observed association rate constants (k_obs_). Fitting the observed data to the equation k_obs_ = k_on_ × [Ca^2+^]^n^ + k_off_ provided the association rate constant (k_on_) and Hill coefficient (n). K_d kinetic_ = (k_off_/k_on_)^1/n^.

To measure dissociation kinetics, protein solution (20 μg/ml) in buffer A supplemented with 1 µM CaCl_2_ was rapidly mixed (1:1) with buffer A supplemented with 10 mM EGTA. Exponential fitting of the fluorescence signal changes over time provided dissociation rate constants (k_off_).

**Mammalian plasmid construction**

In order to construct the pAAV-*CAG*-NES-mCherry plasmid, the NES-mCherry gene was PCR amplified as the BamHI-BsrGI fragment and swapped with the iRFP-P2A-EGFP gene in the pAAV-*CAG*-iRFP-P2A-EGFP vector. In order to construct pAAV-*CAG*-NES-YTnC, pAAV-*CAG*-NES-GCaMP6f, and pAAV-*CAG*-NES-R-GECO1 plasmids, YTnC, GCaMP6f and R-GECO1 were PCR amplified as the BglII-EcoRI fragments and swapped with the mCherry gene in the pAAV-*CAG*-NES-mCherry vector.

In order to construct the pAAV-*CAG*-NES-YTnC-P2A-mCherry, pAAV-*CAG*-NES-NTnC-P2A-mCherry and pAAV-*CAG*-NES-EGFP-P2A-mCherry plasmids, the YTnC, NTnC, and EGFP genes were PCR amplified as BglII-EcoRI fragments and swapped with the GCaMP gene in the pAAV-*CAG-*NES-GCaMP-P2A-mCherry vector.

In order to construct the pAAV-*CAG*-NES-R-GECO1-P2A-NES-YTnC and pAAV-*CAG*-NES-R-GECO1-P2A-NES-GCaMP6s plasmids, the YTnC and GCaMP6s genes were PCR amplified as BglII-EcoRI fragments and swapped with the mCherry gene in the pAAV-*CAG-*NES-R-GECO1-P2A-NES-mCherry vector.

In order to construct the pAAV-*CAG*-Mito-YTnC, and pAAV-*CAG*-Mito-GCaMP6s plasmids, the YTnC, and GCaMP6s genes were PCR amplified as BglII-EcoRI fragments and swapped with the mCherry gene in the pAAV-*CAG-*Mito-mCherry vector.

In order to construct the pAAV-*CAG*-IMS-mCherry plasmid, the IMS gene was PCR amplified as AgeI-BglII fragment and swapped with the Mito gene in the pAAV-*CAG-*Mito-mCherry vector.

In order to construct the pAAV-*CAG*-IMS-YTnC, and pAAV-*CAG*-IMS-GCaMP6s plasmids, the YTnC, and GCaMP6s genes were PCR amplified as BglII-HindIII fragments and swapped with the mCherry gene in the pAAV-*CAG-*IMS-mCherry vector.

In order to construct the pAAV-*CAG*-Lyn-YTnC, and pAAV-*CAG*-Lyn-GCaMP6s plasmids, the Lyn-YTnC, and Lyn-GCaMP6s genes were PCR amplified as AgeI-EcoRI fragments and swapped with the Mito-mCherry gene in the pAAV-*CAG-*Mito-mCherry vector.

In order to construct the pAAV-*CAG*-ER-mCherry, pAAV-*CAG*-H2B-YTnC, and pAAV-*CAG*-H2B-GCaMP6s plasmids, the ER and H2B genes were PCR amplified as AgeI-BglII fragments and swapped with the Mito gene in the pAAV-*CAG-*Mito-mCherry, pAAV-*CAG-*Mito-YTnC and pAAV-*CAG-*Mito-GCaMP6s vectors, respectively.

In order to construct the pAAV-*CAG*-ER-YTnC-KDEL, and pAAV-*CAG*-ER-GCaMP6s-KDEL plasmids, the YTnC-KDEL, and GCaMP6s-KDEL genes were PCR amplified as NheI-EcoRI fragments and swapped with the mCherry-KDEL gene in the pAAV-*CAG-*ER-mCherry-KDEL vector.

In order to construct the pAAV-*CAG*-Homer-mCherry plasmid, the Homer gene was PCR amplified as AgeI-NheI fragment and swapped with the Mito gene in the pAAV-*CAG*-Mito-mCherry vector.

In order to construct the pAAV-*CAG*-Homer-YTnC and pAAV-*CAG*-Homer-GCaMP6s plasmids, the YTnC and GCaMP6s genes were PCR amplified as NheI-EcoRI fragments and swapped with the mCherry gene in the pAAV-*CAG*-Homer-mCherry vector.

In order to construct the pCAG-PSD95.FingR-GCaMP6s-CCR5TC and pCAG-PSD95.FingR-GCaMP6s-CCR5TC, the GCaMP6s and YTnC genes were PCR amplified and swapped with eGFP gene in the pCAG-PSD95.FingR-eGFP-CCR5TC plasmid (Addgene plasmids #46295).

In order to construct the pAAV-*CAG*-(YTnC no stop codon) and pAAV-*CAG*-(GCaMP6s no stop codon) plasmids, the (YTnC no stop codon), and (GCaMP6s no stop codon) genes were PCR amplified as AgeI-EcoRI fragments and swapped with the Mito-mCherry gene in the pAAV-*CAG-*Mito-mCherry vector.

In order to construct the pAAV-*CAG*-YTnC-KV2.1, pAAV-*CAG*-YTnC-AIS, pAAV-*CAG*-YTnC-MBD, pAAV-*CAG*-YTnC-KA2, pAAV-*CAG*-GCaMP6s-KV2.1, pAAV-*CAG*- GCaMP6s-AIS, pAAV-*CAG*-GCaMP6s-MBD, pAAV-*CAG*-GCaMP6s-KA2 plasmids, the KV2.1, AIS, MBD and KA2 genes were PCR amplified as EcoRI-HindIII fragments and inserted at EcoRI/HindIII restriction sites of the pAAV-*CAG*-(YTnC no stop codon) and pAAV-*CAG*-(GCaMP6s no stop codon) vectors, respectively.

In order to construct the pAAV-*CAG*-YTnC-β-actin, pAAV-*CAG*-YTnC-α-tubulin, pAAV-*CAG*-GCaMP6s-β-actin, and pAAV-*CAG*-GCaMP6s-α-tubulin plasmids, the β-actin and α-tubulin genes were PCR amplified as NheI-HindIII fragments and swapped with the KV2.1 gene in the pAAV-*CAG*-YTnC-KV2.1 and pAAV-*CAG*-GCaMP6s-KV2.1 vectors, respectively.

**rAAV particles production and isolation**

The rAAV particles were purified as described in original paper[^5^](#_ENREF_5), with some modifications. Briefly, HEK293T cells were grown in ten 15 cm diameter dishes (Greiner Bio-One, Austria) filled with 23 ml of standard DMEM medium supplemented with 10% FBS, Glutamine, 50 U/ml penicillin, and 50 U/ml streptomycin and incubated at 37°C to cell density of 60-80%. Cells were then transfected using the calcium phosphate method with pAAV-DJ (280 μg) and pHelper (280 μg) plasmids mixed with either 280 µg of pAAV-*CAG*-NES-YTnC, pAAV-*CAG*-NES-GCaMP6f, pAAV-*CAG*-GCaMP6s, or pAAV-*CAG*-NES-R-GECO1 plasmids. For transfection, we sequentially mixed 37 ml of aqueous plasmid solution in 250 mM CaCl_2_ with 2xHBS buffer and incubated for 20 min at r.t. The solution was then added drop-wise to the cell cultures in DMEM/Glutamine/p/s without FBS. Cells were incubated with the transfection mix for 16-20 h at 5% CO_2_ and 37°C. After the medium was exchanged for DMEM/10% FBS/Glutamine/p/s and cells were cultured for an additional 48 hours. Afterwards, the cells were washed with DPBS, treated with 0.25% trypsin-EDTA for 5 min at 37 °C, resuspended in DPBS, transferred into 50-mL tubes, and centrifuged at 1,640 g for 1 min. Pellets were then resuspended in 50 ml of 100 mM NaCl, 20 mM Tris-HCl, pH8.0. Next, we added 10% sodium deoxycholate until a final concentration of 0.5% and benzonase until a final concentration of 25 u/ml. After, the cells were incubated for 1 hour at 220 rpm and 37°C. Cellular debris was removed by centrifugation at 1,640 g for 15 min. Viral particles were bound with 1 ml of HiTrap Heparin (GE Healthcare, UK) in 50 mL tubes in batch experiments for 60 min, at r.t. with mixing. After the resin was pelleted down by centrifugation at 1,640 g for 15 min and washed with 3x12 ml of 100 mM NaCl, 20 mM Tris-HCl at pH 8.0. The resin was placed into the column and particles were eluted in 5 mL of 500 mM NaCl, 20 mM Tris-HCl, pH 8.0. Collected particles then were concentrated using centrifugal filter units Amico Ultra-4 (100K, Merk Millipore Ltd., IRL) until a final volume of ~100 μl.

For titration of viral particles HEK293T cells were seeded onto MatTek glass bottom dishes and grown till 60-70% confluency. Each dish was infected with a serial dilution of AAV vector; two dishes were used for each dilution. After 48-72 h, cellular cultures were imaged using an Andor XDi Technology Revolution multi-point confocal system (Andor, UK). The number of transduced cells in the dishes with the highest dilution factor, but still containing infected cells, were counted and titer was determined as the average number of transduced cells multiplied by the dilution factor.

**Mammalian live-cell imaging**

HeLa Kyoto cell cultures were imaged 24-48 h after transfection using a laser spinning-disk Andor XDi Technology Revolution multi-point confocal system (Andor Technology, UK) equipped with an inverted Nikon Eclipse Ti-E/B microscope (Nikon Instruments, Japan), a 75 W mercury-xenon lamp (Hamamatsu, Japan), a 60× oil immersion objective NA 1.4 (Nikon, Japan), a 16-bit Neo sCMOS camera (Andor Technology, UK), laser module Revolution 600 (Andor Technology, UK), spinning-disk module Yokogawa CSU-W1 (Andor Technology, UK), and a cage incubator (Okolab, Italy). The green and red fluorescence were acquired using 80% of the 488 nm (17.3 µW/cm^2^ before objective lens) and 80% of 561 nm (62.3 µW/cm^2^ before objective lens) laser powers, confocal dichroic mirror 405/488/561/640 and filter wheel emission filters 525/50 and 617/73, respectively (Supplementary Fig. 23). Before imaging, the culture medium was changed to Dulbecco’s Phosphate Buffered Saline (DPBS) buffered with 20 mM HEPES, pH 7.4 or DMEM medium supplemented with 10% FBS, Glutamine, 50 U/ml penicillin, and 50 U/ml streptomycin buffered with 20 mM HEPES, pH 7.4

For time-lapse imaging experiments with varying Ca^2+^ concentration, 1 mM EDTA and 2.5 μM ionomycin were added to cells for imaging calcium indicators in the Ca^2+^-free state. After imaging calcium indicators in the apo-state, cells were washed with DPBS buffered with 20 mM HEPES, pH 7.4. Next, 2 mM CaCl_2_ and 2.5 μM ionomycin were added to induce fluorescence signal for Ca^2+^-saturated calcium indicators.

**Isolation, transduction, and imaging of neuronal cultures**

Dissociated neuronal cultures were isolated from C57BL/6 mice at postnatal days 0-3 and were grown on 35-mm MatTek glass-bottom dishes in Neurobasal Medium A (GIBCO, UK) supplemented with 2% B27 Supplement (GIBCO, UK), 0.5 mM glutamine (GIBCO, UK), 50 U/ml penicillin, and 50 μg/ml streptomycin (GIBCO, UK). On the 4^th^ day *in vitro*, neuronal cells were transduced with 1-2 μl rAAV viral particles carrying AAV-*CAG*-NES-YTnC, AAV-*CAG*-NES-R-GECO1, or AAV-*CAG*-NTnC. Cells were imaged using an Andor XDi Technology Revolution multi-point confocal system as described above.

**Whole-cell electrophysiology and calcium imaging**

Whole-cell recordings with patch electrodes were made from cultured neurons, expressing GECIs. Cells were selected under visual control using standard filter sets for green and red fluorescence and DIC infrared video microscopy. The patch electrodes were filled with a potassium gluconate-based solution (130 mM potassium gluconate, 20 mM KCl, 4 mM Mg-ATP, 0.3 mM Na_2_-GTP, 10 mM sodium phosphocreatine, 10 mM HEPES at pH 7.3) and had a resistance of 6–8 MΩ. During recording, cells were bathed in modified Hank’s solution containing: 138 mM NaCl, 1.26 mM CaCl_2_, 0.5 mM MgCl_2_, 0.4 mM MgSO_4_, 5.3 mM KCl, 0.44 mM KH_2_PO_4_, 4.16 mM NaHCO_3_, 0.34 mM Na_2_HPO_4_, 10 mM Glucose, 10 mM HEPES at pH 7.4 and room temperature. Recordings were made with a MultiClamp 700B (Molecular Devices, USA) amplifier in the bridge mode. After amplification and low-pass filtering at 10 kHz, data were digitized at 20 kHz and fed into a computer using the Digidata 1500 interface and pCLAMP software (Molecular Devices, USA). Cells were stimulated with 50 Hz trains of short (5ms) intracellularly applied current pulses; the intensity of the pulses was adjusted to reliably induce action potentials for each cell.

Optical imaging was performed on an Olympus BX51WI microscope equipped with 40× water immersion objective, two camera ports, and collimated light emitting diodes (LED) with the peak emission wavelength of 470 nm (for YTnC and GCaMP6s, emitting power 350mW) and 530 nm (for R-GECO1, emitting power 170mW) (Thorlabs, USA) for epi-illumination. Imaging was performed with a NeuroCCD camera (80 × 80 pixels, no electron multiplication, RedShirtImaging, USA) using a frame rate of 40 Hz. Fluorescence changes were measured with single wavelength excitation and emission >510 nm for green and >610 nm for red fluorescence. Both light emitting diodes were used at 10% of their maximal power. Power density measured after 40X Olympus objective with S130C/PM16 Power Meter (Thorlabs, USA) was 0,32 mW/mm^2^ for blue light and 1 mW/mm^2^ for green light. Analysis of optical data, including spatial averaging, high-pass and low-pass filtering, was conducted with the Neuroplex 7 software (RedShirtImaging, USA). The time-courses of the responses were corrected for bleaching using a linear regression computed through the mean values 2 seconds before the stimulation and by subtracting the extrapolated values.

**Surgery for V1 *in vivo* two-photon imaging.**

*In vivo* imaging was performed on C57BL/6 mice (Jackson Laboratory, USA) infected with rAAV particles carrying YTnC indicator under the control of the CAG promoter (AAV-*CAG*-NES-YTnC). For cranial window implantation, mice were anesthetized using mixture of zoletil (40 mg/kg) and xylazine (5 mg/kg). As the local anesthetic novocaine (5mg/ml) solution was injected subcutaneously. The moisturizing gel Viscotears (Novartis Healthcare, Switzerland) was applied on the surface of eyes to protect of drying. During the operation the mouse's head was fixed in stereotaxic frame (Kopf Instruments, USA). The rAAV particles were injected into the layer 2/3 of the primary visual cortex (V1) in accordance with coordinates from anatomical brain atlas (centered 2.6 mm lateral, 2.9 mm posterior and 0.3-0.4 mm ventral to the bregma). The volume of 0.5 μl of rAAV particles was injected through a glass micropipette with an Ultra Micro Pump 3 (World Precision Instruments, USA). Next, craniotomy (3 mm diameter) was performed over V1 in accordance with a previously described protocol[^6^](#_ENREF_6). Then, a 5-mm round glass coverslip (Menzel, Thermo Fisher, Germany) was attached to the skull using cyanoacrylate glue for glass. A Neurotar (Neurotar Ltd., Finland) head post was cemented to the skull with dental cement (Stoelting, USA) and was later used for mouse head fixation under microscope objective.

**Two-photon *in vivo* mouse imaging in V1.**

Two-photon imaging of V1 neurons was performed 90–100 days after viral transduction using an Olympus MPE1000 two-photon microscope equipped with a Mai Tai DeepSee Ti:Sapphire femtosecond-pulse laser (Spectra-Physics, USA) and a water-immersion objective lens, 20× 1.05 NA (Olympus, USA) using optical configuration for registration of green fluorescence and grating specific signal (Supplementary Fig. 25). A wavelength of 960 nm was used for excitation (20 mW/cm^2^ at 5% power, measured before objective lens). Imaging was performed with the Olympus Fluoview Software Version 3.1. Mice were kept anaesthetized with urethane (1.5 mg/kg). Images of L2/3 neurons (approximately 80-100 μm deep from pia) were recorded at 0.82-1.25 frames per second continuously, with different fields of view from 140×150 to 230×250 μm with exposure time 12.5μs/pix. Imaging of spine dynamic was performed at 5-15 μm from pia at 1.25-1.34 frames per second continuously, with different fields of view from 30×44 to 63×80 μm with exposure time 10 μs/pix. As visual stimuli we used moving gratings generated using PsychoPy^[7](#_ENREF_7)^, which were presented using an LCD monitor (30 × 50 cm) placed 32 cm in front of the left eye of the mouse. Each stimulus trial consisted of a 10-s blank period (uniform dark display at mean) followed by 10-s drifting sinusoidal grating (0.05 cycles/degree, 2 Hz temporal frequency, 8 different directions).

**Analysis of V1 two-photon functional imaging**

Image analysis was performed in Olympus Fluoview Software Version 3.1 and custom Python scripts. Regions of interest (ROIs) corresponding to identifiable cell bodies were selected manually in the Olympus Fluoview Software Version 3.1. The ΔF/F_0_ of each trial was calculated as (F-F_0_)/F_0_, where F_0_ was the fluorescence averaged over a 2-s period immediately before the start of visual stimulation..^[8](#_ENREF_8)^

**Animals and surgery for imaging with an nVista HD miniature microscope**

Twelve adult male C57BL/6 mice, aged 20 weeks at the start of the experiments, were used for this study. Mice underwent two surgical procedures under zoletil-xylazine anesthesia (40 and 5 mg/kg, respectively). First, a circular 2-mm-diameter craniotomy was made, and 500 nl of rAAV viral particles (carrying AAV-*CAG*-NES-YTnC, AAV-*CAG*-NES-GCaMP6f, or AAV-*CAG*-GCaMP6s) was injected through a 50 µm tip diameter glass micropipette (Wiretrol I, 5-000-1001, Drummond, USA) into two areas of the hippocampus: CA1 (left hemisphere; stereotaxic coordinates:−1.9 mm A/P from bregma, −1.4 mm M/L, −1.3 mm D/V) or DG (left hemisphere: stereotaxic coordinates: −2.0 mm A/P from bregma, −1.6 mm M/L, −2.0 D/V). Microinjections were performed using UltraMicroPump with Micro4 Controller (WPI, USA) at a rate of 100 nl/min. All exposed surfaces of the brain tissue were sealed with KWIK-SIL silicone adhesive (WPI Inc., USA). Two weeks later, the silicone was removed, and the dura matter was extracted from the craniotomy site. Then, a GLP 1040 (for CA1) and GLP 0561 (for DG) lens probes (Inscopix Inc., USA) was lowered slowly to a depth of 1.1 mm (for CA1) or 1.8 mm (for DG) while constantly washing the craniotomy site with sterile cortex buffer. Next, all the exposed tissue was sealed with KWIK-SIL and white dental cement (Stoelting, USA).

**Ca^2+^ in vivo imaging with the nVista HD miniature microscope**

After at least two weeks recovery period, mice were anesthetized again, and baseplates for attaching the portable nVista HD miniature microscope (Inscopix Inc., USA) were mounted onto the dental acrylic caps. A few days after baseplate mounting, we sequentially attached the nVista HD microscope to awake mice that were then placed in a circular track. 10 min long Ca^2+^ activity movies of awake, freely moving mice were captured at a frame rate of 20 Hz and at constant LED power of 1mW. The optical scheme of nVista HD microscope consists of a blue LED with ~470 nm spectral peak, a drum lens as the collector, a 480/40 nm fluorescence excitation filter ,a dichroic mirror, a 0.245 pitch length, 0.45 NA 2-mm-diameter GRIN objective, a 535/50 nm emission filter, an achromatic lens (f = 15 mm) and the CMOS image sensor, as described in details in reference.[^9^](#_ENREF_9) To record mouse behavior, ASUS ZenFone 2 Z00ED camera was used; synchronization was achieved using microscope's LED blinking. Image analysis for the acquired calcium data was performed using Mosaic software (Inscopix Inc., USA) and custom MATLAB scripts (Supplementary Methods).

**Image analysis with a NVista HD miniature microscope**

All processing of calcium imaging data was made using the Mosaic software (Inscopix Inc.), and custom MATLAB scripts. First, all movies were spatially down-sampled by a factor of 2 in order to increase computation speed. Then, rigid body registration was made using a Mosaic routine based on TurboReg algorithm to correct lateral displacements of the focal plane[^6^](#_ENREF_6). After this, ΔF/F normalization was applied to the movies: ΔF/F = (F – F_0_)/F_0_, where F_0_ is intensity value for each pixel, averaged over time. For cell identification, spatial filters corresponding to individual cells were obtained using principal component analysis, followed by independent component analysis (PCA/ICA)[^10^](#_ENREF_10). After a threshold of 50% of the filter's maximum intensity was applied to each filter, all pixels with values lower than a threshold were set to zero. Filters with thresholds with low circularity, noisy appearance, and non-smooth borders were manually excluded from further analysis. After this, activity traces were extracted by applying threshold filters to ΔF/F movies. To correct neuropil contamination, for each trace the neuropil correction was made according to following equation: F_true_(t) = F(t) – F_neuropil_(t), where F(t) is an extracted trace, and F_neuropil_(t) is a mean trace of all pixels inside 50 μm circle, with center at the brightest point of threshold filter; all threshold filters were excluded from this circle.

Calcium events (spikes) detection was performed whenever difference between a trace amplitude and its median value crossed the threshold of 4 median absolute deviations (MADs, were calculated for each cell over the whole trace). The peak was approximated by function , where *t_0_* is spiking time, *τ_rise_*, *τ_off_* - typical rise and decay times (not to be confounded with half-rise and half-decay times!), *A* – scale factor, *B* – background level and *θ* — Heaviside step function. To allow the detection of succeeding spikes after the given event, its fit was considered as a background for the next spike. Rise and decay half-times were measured as the times from the peak to half-peak on the left and right sides of the mean spike, respectively (Supplementary Fig. 9). Finally, signal-to-noise ratio (SNR) was quantified as peak ΔF/F response over 1 MAD.

**Ethical approval and animal care**

All methods for animal care and all experimental protocols were *approved* by the National Research Center “Kurchatov Institute” Committee on Animal Care (protocol No. 1, 7 September 2015) and were *in accordance* with the Russian Federation Order Requirements N 267 МЗ and the National Institutes of Health Guide for the Care and Use of Laboratory Animals. Nineteen and two C57BL/6 mice were used in this study, ages ~2-4 months and P0-P2 old, respectively. Mice were used without regard to gender.

**Supplementary References**

1. Subach, F.V. et al. Photoactivatable mCherry for high-resolution two-color fluorescence microscopy. *Nat. Methods* **6**, 153-159 (2009).

2. Subach, O.M. et al. Conversion of red fluorescent protein into a bright blue probe. *Chem. Biol.* **15**, 1116-1124 (2008).

3. Zhao, Y. et al. An expanded palette of genetically encoded Ca(2) indicators. *Science* **333**, 1888-1891 (2011).

4. Shaner, N.C., Steinbach, P.A. & Tsien, R.Y. A guide to choosing fluorescent proteins. *Nat Methods* **2**, 905-909 (2005).

5. McClure, C., Cole, K.L., Wulff, P., Klugmann, M. & Murray, A.J. Production and titering of recombinant adeno-associated viral vectors. *J Vis Exp*, e3348 (2011).

6. Holtmaat, A. et al. Long-term, high-resolution imaging in the mouse neocortex through a chronic cranial window. *Nature protocols* **4**, 1128-1144 (2009).

7. Peirce, J.W. Generating Stimuli for Neuroscience Using PsychoPy. *Front Neuroinform* **2**, 10 (2008).

8. Chen, T.W. et al. Ultrasensitive fluorescent proteins for imaging neuronal activity. *Nature* **499**, 295-300 (2013).

9. Ghosh, K.K. et al. Miniaturized integration of a fluorescence microscope. *Nature methods* **8**, 871-878 (2011).

10. Mukamel, E.A., Nimmerjahn, A. & Schnitzer, M.J. Automated analysis of cellular signals from large-scale calcium imaging data. *Neuron* **63**, 747-760 (2009).
